# Fimbriae reprogram host gene expression – Divergent effects of P and type 1 fimbriae

**DOI:** 10.1371/journal.ppat.1007671

**Published:** 2019-06-10

**Authors:** Ines Ambite, Daniel S. C. Butler, Christoph Stork, Jenny Grönberg-Hernández, Bela Köves, Jaroslaw Zdziarski, Jerome Pinkner, Scott J. Hultgren, Ulrich Dobrindt, Björn Wullt, Catharina Svanborg

**Affiliations:** 1 Department of Microbiology, Immunology and Glycobiology, Institute of Laboratory Medicine, Lund University, Klinikgatan, Lund, Sweden; 2 Institute of Hygiene, University of Münster, Mendelstr, Münster, Germany; 3 Institute for Molecular Biology of Infectious Diseases, University of Würzburg, Würzburg, Germany; 4 Department of Molecular Microbiology, Washington University School of Medicine, St Louis, Missouri, United States of America; 5 Center for Women's Infectious Disease Research (CWIDR), Washington University School of Medicine, St Louis, Missouri, United States of America; University of California Davis School of Medicine, UNITED STATES

## Abstract

Pathogens rely on a complex virulence gene repertoire to successfully attack their hosts. We were therefore surprised to find that a single fimbrial gene reconstitution can return the virulence-attenuated commensal strain *Escherichia coli* 83972 to virulence, defined by a disease phenotype in human hosts. *E*. *coli* 83972*pap* stably reprogrammed host gene expression, by activating an acute pyelonephritis-associated, *IRF7-*dependent gene network. The PapG protein was internalized by human kidney cells and served as a transcriptional agonist of IRF-7, IFN-β and MYC, suggesting direct involvement of the fimbrial adhesin in this process. IRF-7 was further identified as a potent upstream regulator (-log (*p*-value) = 61), consistent with the effects in inoculated patients. In contrast, *E*. *coli* 83972*fim* transiently attenuated overall gene expression in human hosts, enhancing the effects of *E*. *coli* 83972. The inhibition of RNA processing and ribosomal assembly indicated a homeostatic rather than a pathogenic end-point. In parallel, the expression of specific ion channels and neuropeptide gene networks was transiently enhanced, in a FimH-dependent manner. The studies were performed to establish protective asymptomatic bacteriuria in human hosts and the reconstituted *E*. *coli* 83972 variants were developed to improve bacterial fitness for the human urinary tract. Unexpectedly, P fimbriae were able to drive a disease response, suggesting that like oncogene addiction in cancer, pathogens may be addicted to single super-virulence factors.

## Author summary

Urinary tract infections affect millions of individuals annually, and many patients suffer from recurring infections several times a year. Antibiotic resistance is increasing rapidly and new strategies are needed to treat even these common bacterial infections. One approach is to use the protective power of asymptomatic bacterial carriage, which has been shown to protect the host against symptomatic urinary tract infection. Instilling “nice” bacteria in the urinary bladder is therefore a promising alternative approach to antibiotic therapy. In an effort to increase the therapeutic use of asymptomatic bacteriuria, we reintroduced bacterial adhesion molecules into the therapeutic *Escherichia coli* strain 83972 and inoculated patients who are in need of alternative therapy. To our great surprise, the P fimbriated variant caused symptoms, despite lacking other virulence factors commonly thought to be necessary to cause disease. In contrast, type 1 fimbriae, did not provoke symptoms but enhanced the beneficial properties of the wild-type strain. This is explained by a divergent effect of these fimbrial types on host gene expression, where P fimbriae activate the IRF-7 transcription factor that regulates pathology in infected kidneys, suggesting that a single, potent virulence gene may be sufficient to create virulence in human hosts.

## Introduction

Mucosal surfaces provide ideal living conditions for the normal flora but paradoxically, they also serve as attack sites for numerous bacterial pathogens that cause extensive morbidity and mortality. Understanding this dichotomy is critical for efforts to selectively target and remove pathogens without disturbing the commensal flora or its protective effects. The complex nature of disease predicts that virulence is multifaceted and that pathogens need multiple virulence factors to initiate tissue attack, disrupt immune homeostasis and create symptoms and pathology [1–8]. It is also well established that commensals fail to cause disease, due to a lack of critical virulence genes [9, 10]. About 50% of asymptomatic bacteriuria (ABU) isolates have a smaller genome size than acute pyelonephritis strains due, in part, to inactivating virulence gene deletions or point mutations [11–13]. These strains continue to accumulate loss of function mutations *in vivo*, supporting the notion of a virtually irreversible reductive evolution process, where spontaneous recovery of a virulent phenotype is not likely to occur.

Surprisingly, these loss-of-function mutations also affect fimbrial subunits and adhesin genes [13], which are thought to be essential for bacterial persistence at mucosal sites [14–21]. Adhesive ligands arm bacteria with molecular tools to identify preferred tissue sites and attachment plays a decisive role in colonization and long-term adaptation. Certain fimbrial adhesins are ubiquitously expressed by commensals and pathogens alike, suggesting a homeostatic role. Others, in contrast, show a strong disease association in epidemiologic studies [14], suggesting more direct effects on pathobiology [22].

The urinary tract supports ABU; a commensal-like state [23], which has been shown to prevent super-infection with more virulent strains [24–27]. To reproduce this protective effect, we have established a protocol to create ABU, by inoculating patients with the ABU strain *E*. *coli* 83972 [28, 29]. The therapeutic efficacy and safety of this procedure has been documented in placebo-controlled studies in patients with incomplete bladder voiding [30]. Genome sequencing of *E*. *coli* 83972 has revealed a general “loss of virulence” phenotype, which includes fimbrial genes [13, 31–33]. *E*. *coli* 83972 lacks functional P or type 1 fimbriae, due to attenuating point mutations in the *papG* adhesin gene and a large, inactivating deletion in the *fim* gene cluster [13]. Both fimbrial types have been proposed to enhance bacterial persistence in the urinary tract.

## Results

### Reconstitution of the chromosomal *pap* and *fim* gene clusters in *E*. *coli* 83972

The aim of this study was to increase the efficiency of *E*. *coli* 83972 inoculation and extend its use to include UTI-prone patients with complete bladder voiding. To achieve this goal we equipped *E*. *coli* 83972 with functional adhesins, previously shown to enhance bacterial persistence in the murine or human urinary tract [34, 35]. This approach also made it possible to address how fimbriae affect clinical outcome in inoculated human hosts.

**Fig 1:**
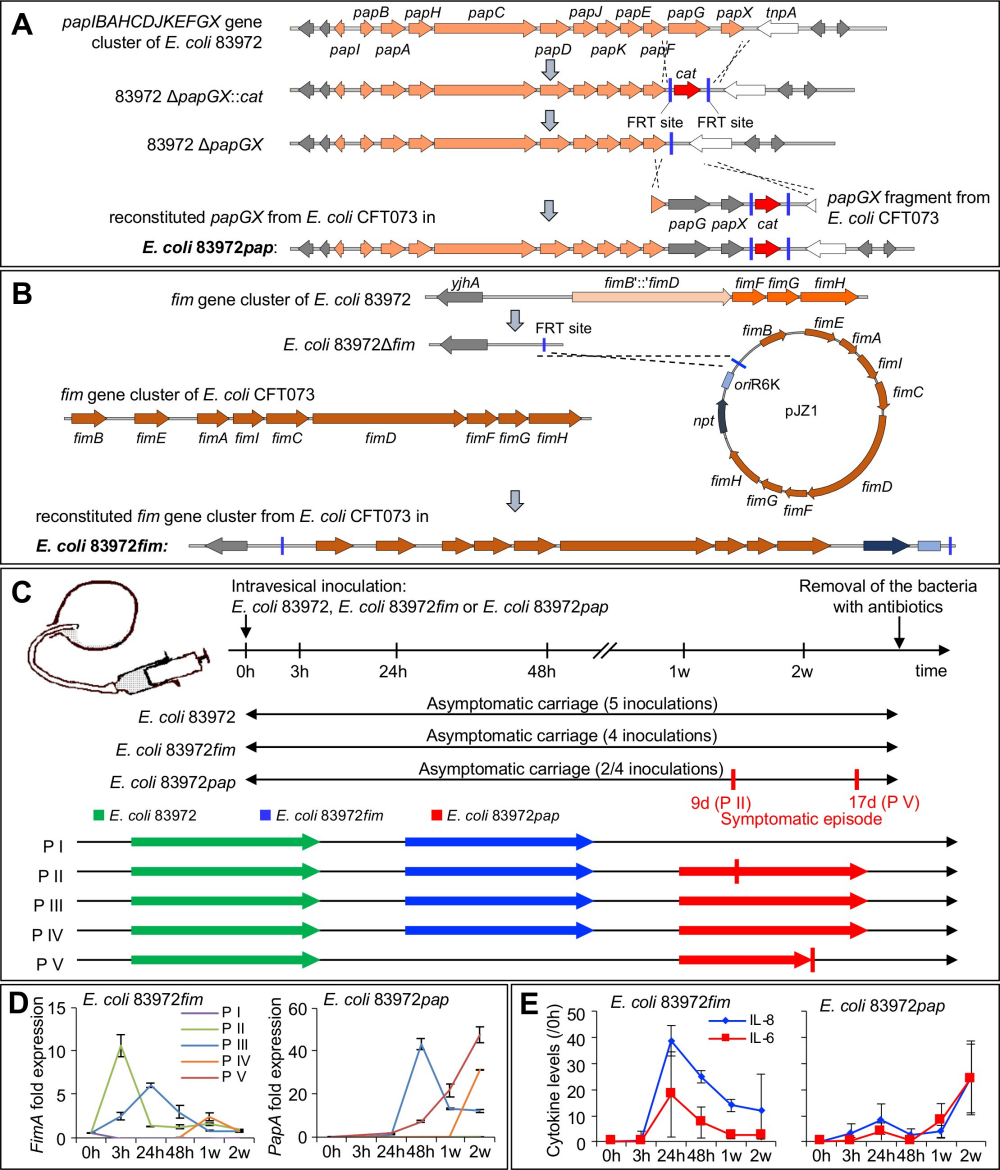
Fimbriated E. coli 83972 variants; construction and human inoculation. **A.** The ABU strain *E. coli* 83972, does not express functional P or type 1 fimbriae, due to chromosomal *PapG* point mutations and a *fimB-D* deletion. In this study, the *pap* or *fim* gene clusters were reconstituted in *E. coli* 83972*pap* and *E. coli* 83972*fim*, respectively. To replace the defective *papG* gene with a functional copy, a *papGX* deletion mutant (*E. coli* 83972Δ*papGX*) was generated using lambda red homologous recombination [59]. Briefly, the chloramphenicol acetyltransferase gene (*cat*) cassette of plasmid pKD3 was amplified with overhangs homologous to the 5´- and 3´-regions of the *E. coli* 83972 *papGX* gene fragment and cured upon transformation with plasmid pCP20 [60]. Meanwhile, the functional *papGX* genes from UPEC strain CFT073 was amplified with homologous overhangs to the *tnpA* and *papF* regions of *E. coli* 83972 and used for electroporation into *E. coli* 83972Δ*papGX* cells. Chromosomal reconstitution of the functional *papGX* genes in the *E. coli* 83972 chromosome was achieved via homologous recombination [59]. **B.** *E. coli* 83972 carries an internal 4,253-bp *fim* deletion, comprising the *fimEAIC* genes and truncated *fimB* and *fimD* genes. To reconstitute the *fim* operon, truncated genes were replaced via lambda red-mediated recombination by a *cat* cassette [59], flanked by two FRT sites and removed by FLP recombinase-mediated recombination [60]. The resulting 83972Δ*fim* strain was transformed with pCP20 and suicide vector pJZ1, carrying the entire *fim* operon from pPKL4 [36]. Chromosomal integration of the entire suicide vector including the functional *fim* operon in *E. coli* 83972*fim* resulted in a functional *fim* copy. **C.** The human therapeutic inoculation protocol, indicating individual patients (P I–P V) and strains (*E. coli* 83972, *E. coli* 83972*fim* or *E. coli* 83972*pap*). Five patients were inoculated with *E. coli* 83972, which established ABU for a period of at least two weeks [28, 30]. After clearance of the strain by a short course of antibiotics, three patients were re-inoculated with *E. coli* 83972*fim*, followed after termination of bacterial carriage by a third inoculation with *E. coli* 83972*pap* (P II, P III and P IV). P I received *E. coli* 83972 followed by *E. coli* 83972*fim* but not *E. coli* 83972*pap*, and P V received *E. coli* 83972 followed by *E. coli* 83972*pap* but not *E. coli* 83972*fim* (**S1 Table**). **D.** Kinetics of *papA* and *fimA* expression after human inoculation. Bacterial RNA was isolated directly from urine of each patient at the indicated time points and *papA* or *fimA* expression was quantified by qRT-PCR. Changes in gene expression were defined relative to *frr* (ribosome-recycling factor) expression. Values for 0h correspond to the bacterial *in vitro* culture used for inoculation. **E.** Kinetics of the urine cytokine response to *E. coli* 83972*fim* (P I–P IV, left) *P* < 0.01 (**) or *E. coli* 83972*pap* (P II–P V, right) *P* < 0.01 (**). Data was normalized by subtraction of the pre-inoculation values in each patient (0h). Mean ± s.e.m. of 4 samples, 2-way ANOVA.

The chromosomal *pap* and *fim* operons were reconstituted using lambda Red-mediated recombination (**Fig 1A** and **1B**). Briefly, *papGX* was deleted (ABU 83972Δ*papGX*) and replaced by functional *papGX* genes from uropathogenic *E*. *coli* (UPEC) strain *E*. *coli* CFT073, via homologous recombination, using pKD3 (*E*. *coli* 83972*pap*, **Fig 1A**) The *fim* operon was reconstituted by replacing an internal 4,253-bp *fim* deletion, comprising the *fimEAIC* genes and truncated *fimB* and *fimD* genes, with the entire *fim* operon from pPKL4 [36] (*E*. *coli* 83972*fim*, **Fig 1B**).

*E*. *coli* 83972*pap* expressed functional P fimbriae as shown by P blood group specific agglutination of human erythrocytes (**S1A Fig**) and attachment to human kidney cells (**S1B Fig**). *E*. *coli* 83972*fim* expressed functional type 1 fimbriae, as shown by α-D-methyl-mannose reversible agglutination of human and guinea pig erythrocytes and adherence to human kidney cells (**S1A–S1C Fig**). The *in vitro* growth rates of *E*. *coli* 83972*pap* and *E*. *coli* 83972*fim* were unchanged, compared to *E*. *coli* 83972 (**S1D Fig**).

### Clinical response and fimbrial expression *in vivo*

In this longitudinal study, five patients (P I to P V) were sequentially inoculated; first with the ABU strain *E*. *coli* 83972 and then with the fimbriated variants of this strain (**Fig 1C**). Each patient contributed a pre-inoculation sample as well as samples from five time points following each inoculation, resulting in 18 samples for patients undergoing three- and 12 samples for patients undergoing two inoculations. As a result of the study design, the response to inoculation was defined relative to the pre-inoculation sample in each patient and inoculation, and changes over time were evaluated intra-individually. Significant changes were evaluated intra-individually as well as between patient groups.

*E*. *coli* 83972 and the fimbriated strains established significant bacteriuria within 48 hours of inoculation and persisted for a period of at least 4 weeks or until the patients were treated to remove the strain (**S2 Fig**). Patients, who carried *E*. *coli* 83972 or *E*. *coli* 83972*fim* remained asymptomatic and P III and P IV carried *E*. *coli* 83972*pap* asymptomatically during the entire study period. Two patients, who carried *E*. *coli* 83972*pap*, developed symptoms, however (**Fig 1C**). In P V, symptoms were recorded 17 days after inoculation (fever, general malaise and loin pain, **S2A Fig**). The patient recovered fully after antibiotic treatment, with a drop in C-reactive protein levels from 245 μg/ml to 3.4 μg/ml after 7 days, normal kidney function on follow up and no evidence of focal tissue damage by intravenous excretory contrast tomography. P II reported a transient febrile reaction and local symptoms from the urinary tract on day 9 after *E*. *coli* 83972*pap* inoculation. Inoculations with *E*. *coli* 83972*pap* were therefore discontinued and the study outcome is evaluated here for P I–P V.

Bacterial *fimA* expression increased immediately after inoculation with *E*. *coli* 83972*fim*, followed by a rapid decline. *PapA* expression increased gradually, from 3 hours post inoculation with *E*. *coli* 83972*pap* until the time of symptoms in P V (**Fig 1D**, **S3 Fig**). This difference in activation kinetics may reflect the location of the *fim* and *pap* gene clusters in the bacterial chromosome, as the *fim* gene cluster is part of the core chromosome but *pap* resides in a chromosomal island that also includes other virulence genes, such as *hlyA* [13]. The urine IL-6 and IL-8 responses paralleled fimbrial expression, with an earlier peak for *E*. *coli* 83972*fim* and a later peak for *E*. *coli* 83972*pap* (**Fig 1E**).

Genome-wide microarray analysis was used to address how the patients respond to bacterial inoculation. RNA was harvested from peripheral blood leucocytes immediately before inoculation and at defined times post-inoculation and significantly regulated genes were identified relative to the pre-inoculation sample in each patient (cutoff FC ≥ 2.0). Fimbriae-related effects on transcription were further defined by intra-individual analysis, comparing the response to the *E*. *coli* 83972 and *E*. *coli* 83972*pap* or *E*. *coli 83972fim* inoculations in each individual.

### Rapid reprograming of host gene expression by *E*. *coli* 83972*pap*

*E*. *coli* 83972*pap* activated a rapid and sustained change in gene expression, which was detected after 3 hours (**S4 Fig**) and reached a maximum in P V during the symptomatic episode (1,020 regulated genes, **S5** and **S6A Figs**). A peak response in P II was also observed in connection with the symptomatic episode (2 weeks, **S6B Fig**) but not in P III and P IV, who remained asymptomatic (**S6C** and **S6D Fig**). Intra-individual comparisons of *E*. *coli* 83972*pap* and *E*. *coli* 83972 inoculations detected little overlap, suggesting that *E*. *coli* 83972*pap* activates a novel, strain background independent repertoire of host genes (P V, **S4** and **S5B Figs**). A similar discrepancy was observed between *E*. *coli* 83972*pap* in P V and *E*. *coli* 83972*fim* in P I (**S4B Fig**).

**Fig 2:**
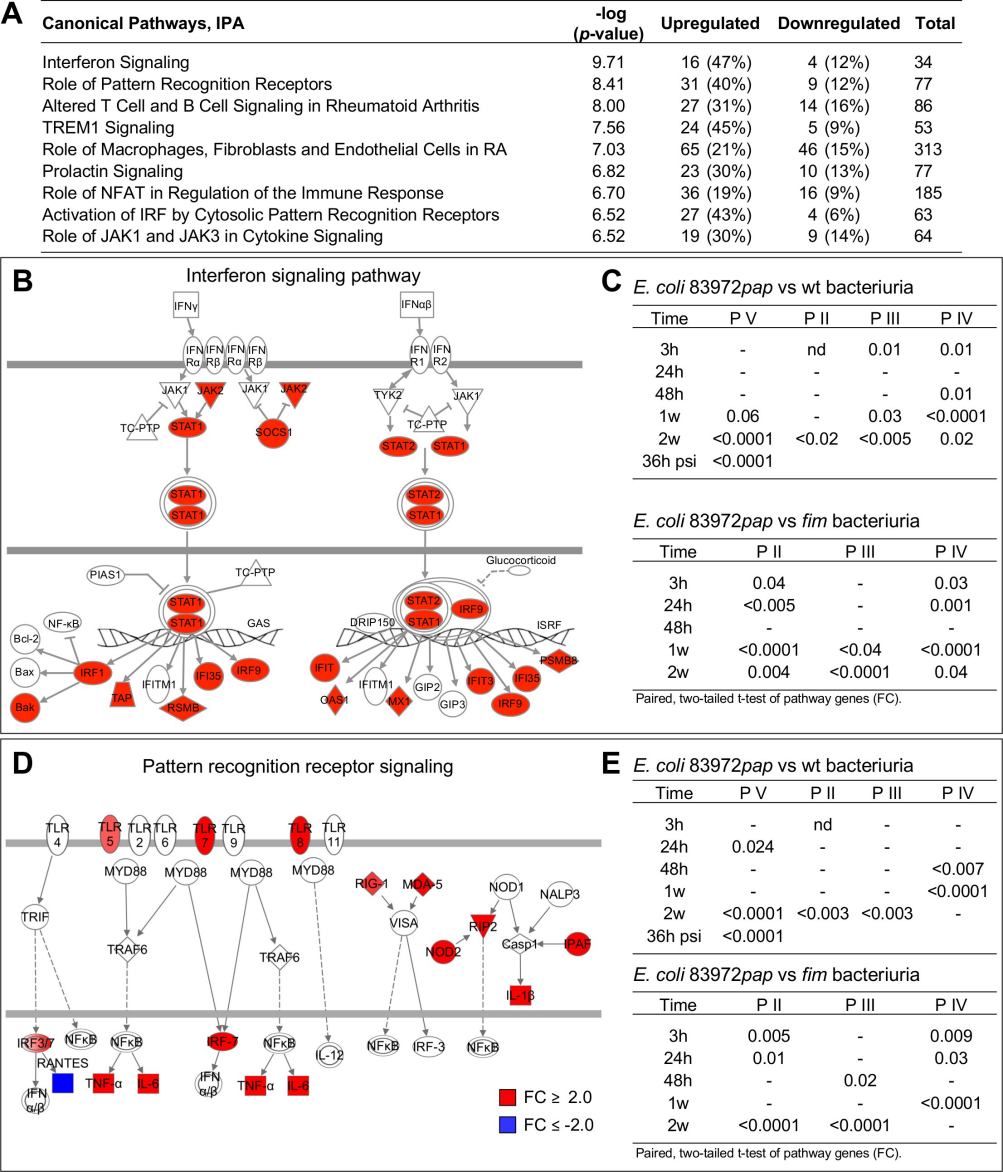
Signaling pathways activated by E. coli 83972pap. **A.** Transcriptomic analysis identifying top regulated canonical pathways in P V at the time of symptoms. **B.** Maximum activation of type I IFN signaling pathway genes in P V at the time of symptoms. **C.** Intra-individual comparison of IFN pathway genes expressed in response to *E. coli* 83972*pap*, *E. coli* 83972 or *E. coli* 83972*fim* (paired *t*-test for each patient and time point, two-tailed values). **D.** Pattern Recognition Receptor (PRR) signaling pathway genes. **E.** Intra-individual comparison of PRR pathway genes in response to *E. coli* 83972*pap*, *E. coli* 83972 or *E. coli* 83972*fim* (paired *t*-test for each patient and time point, two-tailed values). Red = FC ≥ 2.0 and blue = FC ≤ -2.0.

By Gene set enrichment analysis (GSEA) and canonical pathway analysis (**Fig 2A**, **S7 Fig**), type I interferon (IFN) signaling was identified as the top-scoring canonical pathway in P V at the onset of symptoms (**Fig 2A** and **2B**). Genes in this pathway were activated in at least one sample from each patient inoculated with *E*. *coli* 83972*pap* (**S8A Fig**). Significantly regulated genes included *STAT1*, *IFIT1*, *IFIT3*, *MX1* and *PSMB8*. The IFN pathway genes were not regulated in response to *E*. *coli* 83972 or *E*. *coli* 83972*fim*, except in P IV between 3 and 24 hours (**Fig 2C, S8B Fig**).

Furthermore, the pattern recognition receptor (PRR) pathway was activated in all patients inoculated with *E*. *coli* 83972*pap*, including TLRs 2, 4, 5, 7 and 8 (**Fig 2D**, **S8C** and **S9 Figs**) [7]. In P V, regulated genes also included *IRF7*, *OAS3*, *1*, *2*, complement components, *IFIH1* (MDA-5), *DDX58* (RIG-1), *IL1B*, *NLRC4* (IPAF), *TNF*, *RIPK2* (RIP2), *IL6* and *NOD2*. The wild type strain and *E*. *coli* 83972*fim*, in contrast, suppressed the PRR signaling pathway (**Fig 2E, S8D Fig**). The results suggest that P fimbriae “high-jack” the transcriptional machinery of the host, creating a fimbriae-specific gene expression profile.

To address to what extent *pap* reconstitution in an ABU strain creates a disease-like response [7, 8, 37–39], we compared the repertoire of regulated genes in P V at the time of symptoms to the *in vitro* response of human kidney cells to the genetically closely related UPEC strain CFT073 (both phylogroup B2 and same sequence type) [13, 40]. A total of 115 genes were commonly regulated, including *IRF7* and genes involved in interferon- and pattern recognition signaling (**S10 Fig**), suggesting that *E*. *coli* 83972*pap* activates similar facets of the innate immune response as a virulent strain.

### P fimbriae as *IRF7* agonists

**Fig 3:**
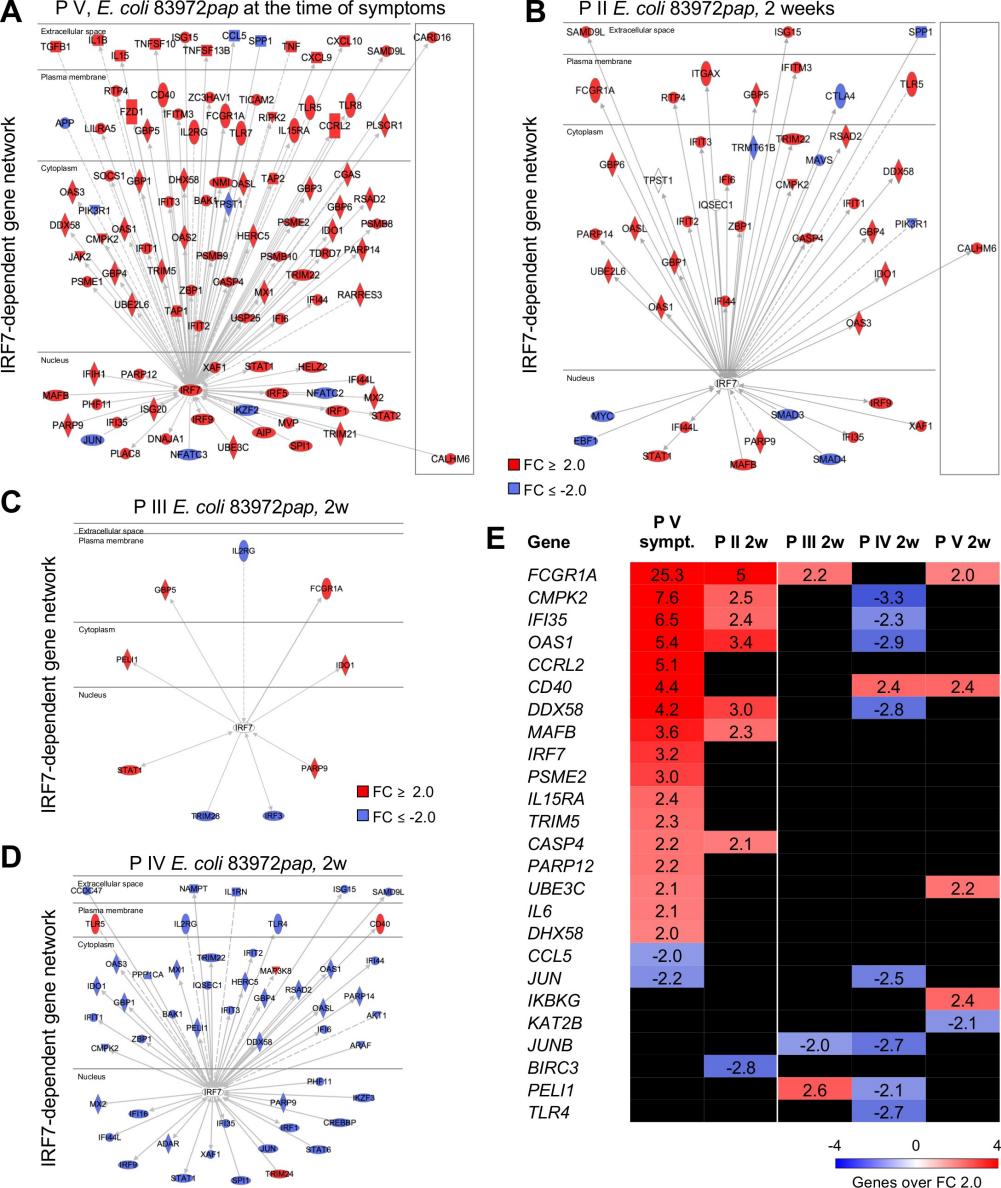
IRF7 activation by E. coli 83972pap. **A.** An IRF-7-centric gene network was activated in P V, at the time of symptoms (*n* = 103). **B.** A more moderate IRF-7 response was detected in P II, 5 days after transient symptoms (*n* = 45, 2 weeks). **C, D.** Patients II and IV, who did not experience symptoms, also did not show evidence of IRF-7 activation. Instead, IRF7-dependent genes were inhibited in P IV (*n* = 39, 2 weeks). **E.** Heatmap of disease associated, IRF-7-driven genes [8] 2 weeks after *E. coli* 83972*pap* inoculation (P II–P V). Genes in the network were activated in P V, at the time of symptoms and a partial response was seen in P II, 5 days after transient symptoms. Red = FC ≥ 2.0 and blue = FC ≤ -2.0.

IRF-7 controls inflammation and renal tissue damage in the murine acute pyelonephritis model, through a network of pathology-associated genes [8]. A potent *IRF7* response was detected in P V at the time of symptoms and a more restricted response in P II, five days after the transient symptoms (**Fig 3A** and **3B**). Furthermore, the *IRF7* response was exclusive for the time of symptoms and was suppressed or not activated in the patients, who did not develop symptoms (**Fig 3C–3E**). Downstream of IRF-7, type I interferon genes and RIG-I pathway genes, cytokines and transcription factors were specifically activated during the symptomatic episode as well as cell surface receptors involved in innate immunity (**Fig 3E** and **S9 Fig**).

**Fig 4:**
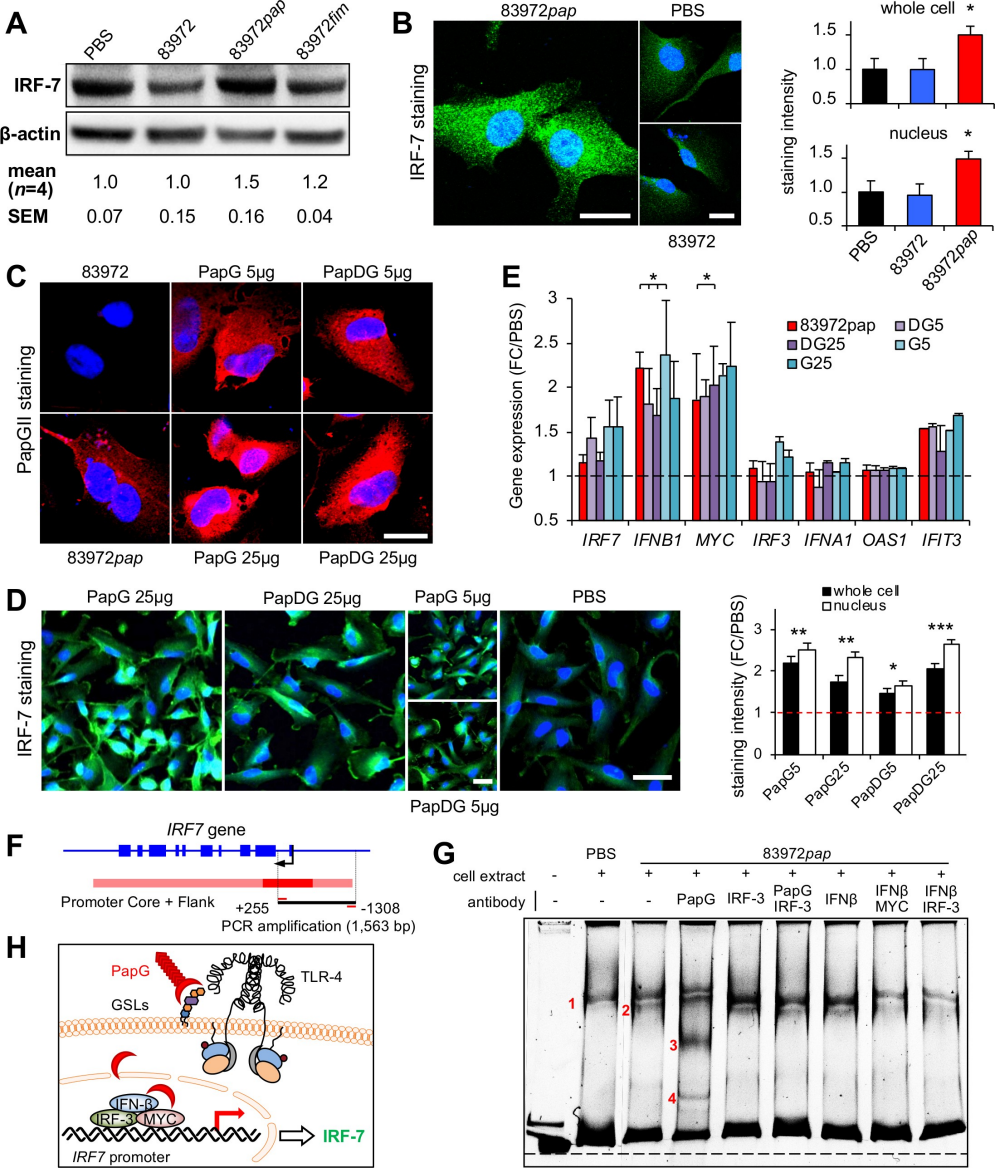
IRF-7 activation by the PapG adhesin. **A.** Increase in IRF-7 protein levels after infection of human kidney cells with *E. coli* 83972*pap* but not *E. coli* 83972*fim* (10^5^ cfu/ml, 4 hours). Western blot analysis. **B.** Increase in nuclear and total IRF-7 staining, quantified by confocal imaging. Mean + s.e.m. of two experiments, 50 cells/experiment. Two-tailed unpaired *t*-test compared to PBS. Scale bars = 20 μm. **C.** PapG internalization after infection or stimulation of cells with purified PapG protein (5–25 μg/ml), quantified by confocal imaging, using polyclonal anti-PapGII antibodies. Scale bar = 20 μm. **D.** The purified PapG adhesin or the PapDG protein complex stimulated an IRF-7 response, in treated cells (5–25 μg/ml). Scale bars = 50 μm. **E.** Increase in *IRF7*, *IFNB1*, *MYC* and *IFIT3* mRNA levels quantified by qRT-PCR. Cells were infected with *E. coli* 83972*pap* or stimulated with the PapG adhesin or the PapDG protein complex (5 or 25 ug/ml). Mean + s.e.m. of two experiments, multiple unpaired *t*-test compared to PBS. **F.** *IRF7* gene and promoter map. *IRF7* promoter DNA (1563bp, -1308 to +255) was used as a probe, in an electrophoretic mobility shift assay (EMSA). **G.** Extracts from uninfected or *E. coli* 83972*pap* infected cells were mixed with the indicated *IRF7* promoter fragment. By polyacrylamide gel electrophoresis, one band shift was detected in uninfected cells (band 1) and a second band in *E. coli* 83972*pap* infected cells (band 2). Specificity for PapG was supported by two super-shifted bands (bands 3 and 4), in the presence of anti-PapG antibody. Bands 3 and 4 were inhibited by using anti-IRF3 antibody. All bands were attenuated by combining anti-IFNβ with anti-IRF-3 or anti-MYC antibodies. **H.** Model of *IRF7* activation by PapG, including IRF-3, IFNβ and MYC. *P* < 0.05 (*), *P* < 0.01 (**), *P* < 0.001 (***).

Consistent with these effects, *E*. *coli* 83972*pap* infection also stimulated a strong IRF-7 response in human kidney cells. Cytoplasmic and nuclear IRF-7 protein levels were increased in *E*. *coli* 83972*pap-*infected cells (**Fig 4A** and **4B**) and internalization of the PapG adhesin [41] was detected, suggesting a P fimbriae-specific effect (**Fig 4C**). This was confirmed by exposing the cells to purified PapG II adhesin protein or the PapDG II protein complex (5 and 25 μg/ml, **Fig 4C, S11A** and **S11B Fig**). IRF-7 expression was activated (**Fig 4D**) as shown by an increase in IRF-7 protein- and *IRF7*, *IFNB1*, *MYC* and *IFIT3* mRNA levels (**Fig 4E**), suggesting that P fimbriae may act as *IRF7* agonists, in a PapG adhesin dependent manner.

Effects of PapG on the assembly of the *IRF7* promoter complex were examined in an electrophoretic mobility shift assay (EMSA), using *IRF7* promoter DNA as a probe (1563bp, -1308 to +255, **Fig 4F** and **4G**). Band shifts were detected when the probe was mixed with protein extracts from uninfected cells (band 1, **Fig 4G**, **S11C Fig**) or *E*. *coli* 83972*pap* infected cells (band 2, **Fig 4G**). Anti-PapG antibodies created a further super-shift (bands 3 and 4) and bands 1 and 2 were strongly attenuated by anti-IRF3-, anti-IFNβ and/or anti-MYC antibodies, consistent with the presence of these proteins in the promoter complex (**Fig 4G** and **4H**). IFN-β and MYC are known to regulate *IRF7* expression by binding to the *IRF7* promoter and IRF-3 forms heterodimers with IRF-7, after activation by phosphorylation [42, 43].

The results suggest that the PapG adhesin affects the assembly of *IRF7* promoter complexes in infected cells, together with IRF-3, IFN-β and/or MYC. Direct binding of purified PapG or PapDG to promoter DNA was not detected, however, suggesting that the other promoter constituents may be required for PapG to bind to the *IRF7* promoter complex.

### Effects of type 1 fimbriae on host gene expression resemble *E*. *coli* 83972

**Fig 5:**
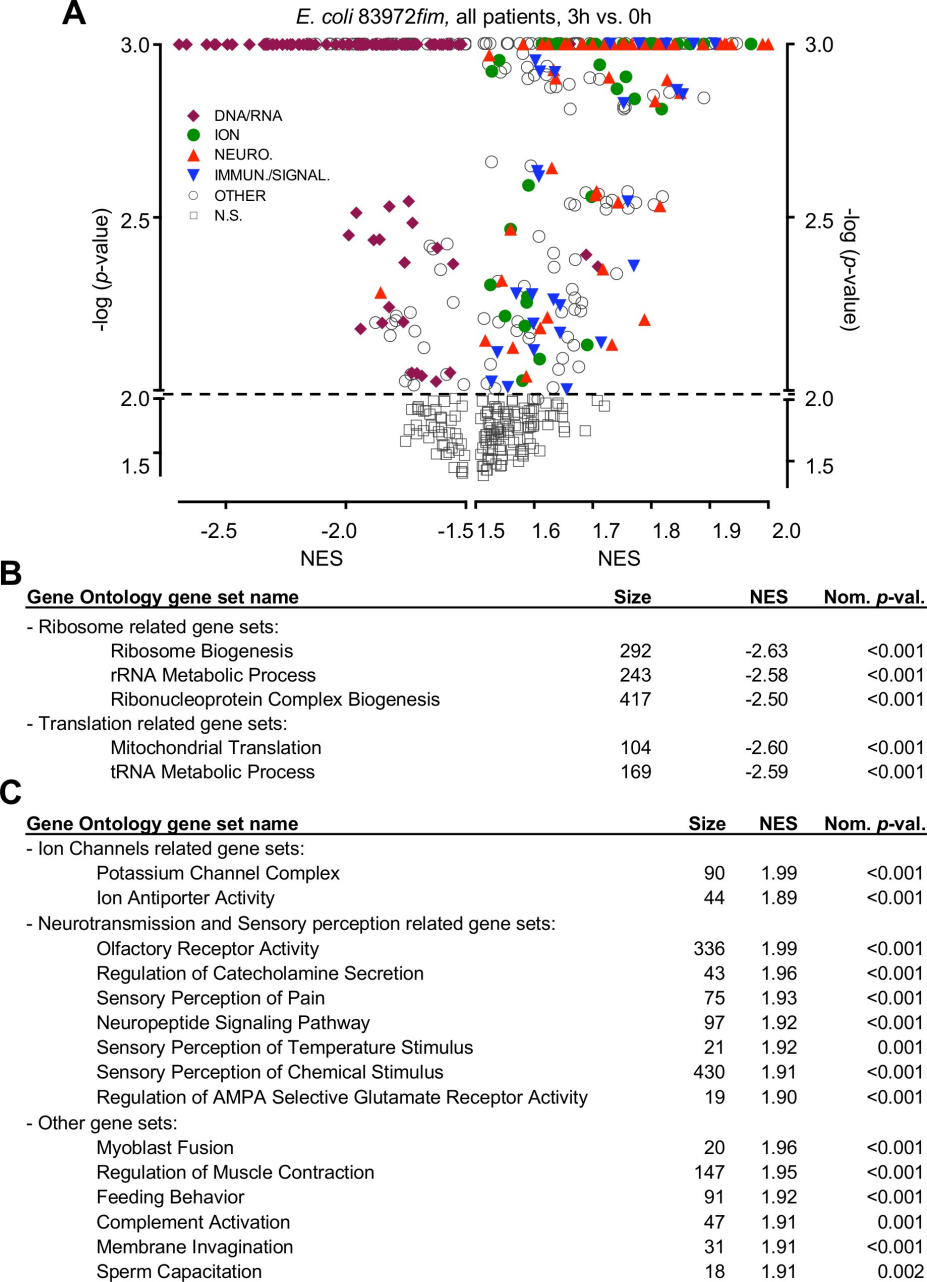
Functional analysis of the early response to E. coli 83972fim inoculation. **A.** Volcano plot of gene sets regulated by *E. coli* 83972*fim* after 3 hours compared to pre-inoculation samples in P I–P IV (Gene Ontology). Identified gene sets are plotted as the -log (*p*-value) against their Normalized Enrichment Score (NES) and functionally annotated (see **S2 Table**). Most inhibited gene sets were involved in RNA processing and translation (purple). Activated genes were mainly involved in ion channel (green)- and neuropeptide (red) regulation as well as immune signaling. **B.** Top five gene sets identified at the time of maximum response to *E. coli* 83972*fim* (3 hours). **C.** Top 15 activated gene sets at the time of maximum response to *E. coli* 83972*fim* (3 hours).

Type 1 fimbriae are ubiquitously expressed among gram-negative bacteria, suggesting a homeostatic role. This study provided a unique opportunity to identify such effects, in inoculated human hosts. We found no evidence that type 1 fimbriae create symptoms or pathology, when expressed in the background of *E*. *coli* 83972. Instead a rapid and profound inhibitory effect was identified in patients inoculated with *E*. *coli* 83972*fim*, by GSEA and analyzed using the gene ontology database (**Fig 5A**). Genes involved in RNA processing and post-transcriptional regulation were inhibited, suggesting effects on the post-transcriptional environment in infected host cells, including predicted inhibition of 5’ RNA capping and 3’ poly(A) tail elongation and translation as well as intron removal, exon splicing and ribosome biogenesis (**Fig 5B, S2 Table**). Pro-inflammatory gene sets were not significantly regulated and by Ingenuity pathway analysis (IPA, **S12 Fig**), a limited number of weakly regulated pathways were identified, including Natural Killer (NK) cell signaling, which was inhibited (**S12B–S12D Fig**) and Integrin signaling, which was moderately activated (**S12E–S12G Fig**).

In addition, a number of gene sets were moderately activated by *E*. *coli* 83972*fim* in inoculated hosts (P I–P IV, 3 hours, **Fig 5C**), including ion channels and genes involved neuropeptide signaling, sensory perception of pain and other stimuli. Gene network analysis further revealed strong similarities between *E*. *coli* 83972 and *E*. *coli* 83972*fim* (**S13** and **S14 Figs**). By aligning these responses, we detected a subset of overlapping genes that was inhibited by both strains, with effects on RNA processing and ribosome biogenesis (**S13A–S13C Fig**). Kinetic analysis suggested that the effects of *E*. *coli* 83972 were accelerated by *E*. *coli* 83972*fim*, suggesting that type 1 fimbriae act, in part, by enhancing the inhibitory effects of *E*. *coli* 83972 (**S13B–S13D Fig**). In contrast, the transcriptional response to *E*. *coli* 83972*fim* and *E*. *coli* 83972*pap* showed no similarity. A few genes were inversely regulated (*n* = 33); activated by *E*. *coli* 83972*pap* but inhibited by *E*. *coli* 83972*fim* (P I, **S14 Fig**).

### Type 1 fimbriae activate ion channel expression

**Fig 6:**
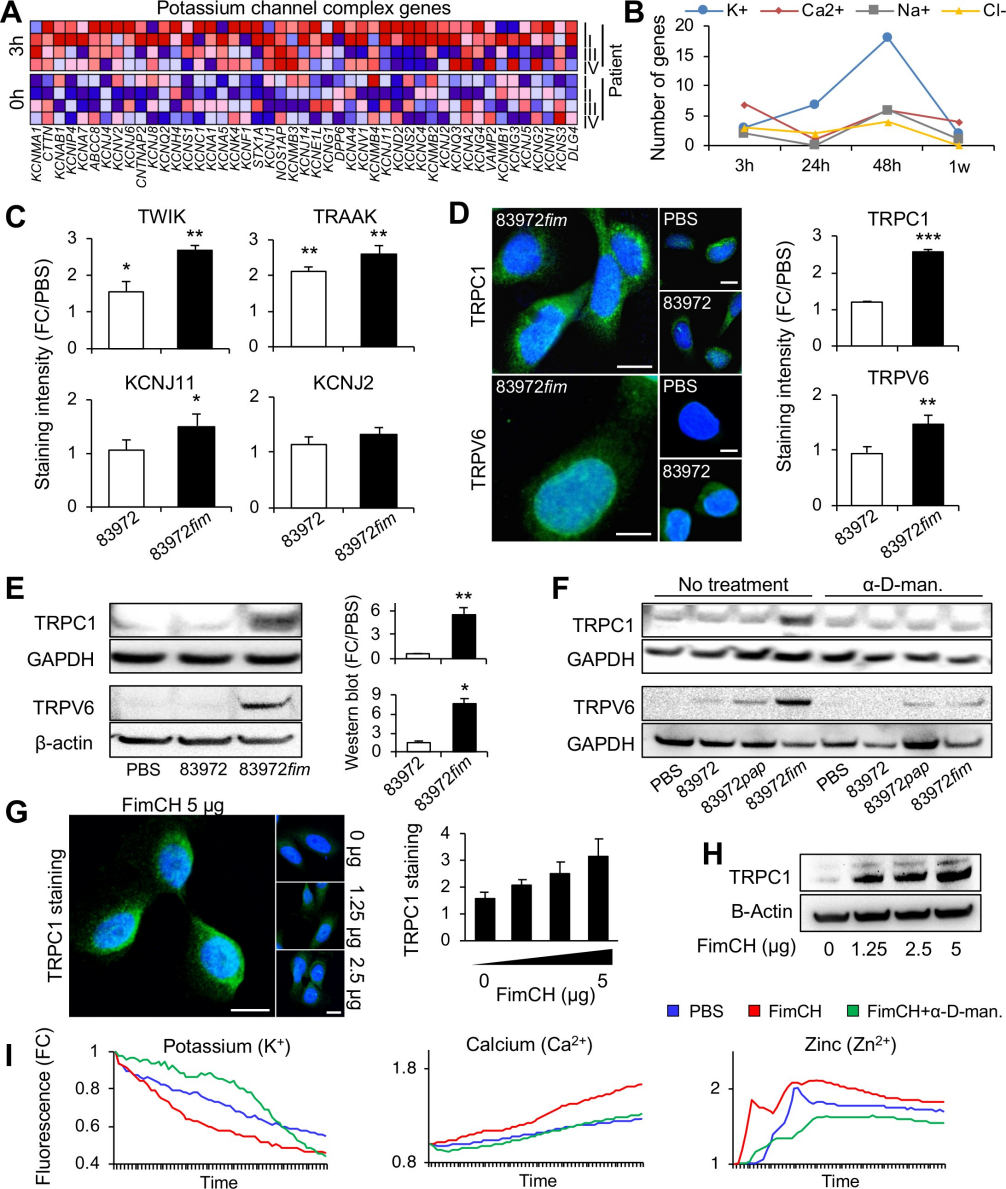
E. coli 83972fim and FimH regulate ion channel expression. **A.** Early activation of potassium channels in patients inoculated with *E. coli* 83972*fim* (*n* = 47). GSEA, 3-hour samples from P I–P IV. **B.** Kinetics of ion channel expression in patients inoculated with *E. coli* 83972*fim*, showing a rapid Ca^2+^ and K^+^ channels response at 3 hours and a sustained K^+^ channels response at 24- and 48 hours. **C-E.** Increased expression of K^+^ channels (TWIK, TRAAK and KCNJ11 but not KCNJ2, **C**) and cation channels (TRPC1 and TRPV6, **D** and **E**) in bladder epithelial cells infected with *E. coli* 83972 or *E. coli* 83972*fim* (10^5^ cfu/ml, 4 hours). Confocal imaging (**C,D**) and Western blot analysis (**E**). Scale bars = 20 μm. **F.** The increase in TRPC1 and TRPV6 expression was effectively blocked by addition of the soluble FimH antagonist α-D-methyl-mannopyranoside (α-D-man., 2.5%). **G,H.** Purified FimCH protein (1.25–5 μg/ml, 4 hours) increased TRPC1 expression in a dose dependent manner. Confocal imaging (**G**) and Western blot analysis (**H**). Scale bars = 20 μm. I. Activation of K^+^, Ca^2+^ and Zn^2+^ fluxes by purified FimCH (5 μg/ml) as determined by fluorescence spectrometry with repeated 20 second-measurements for 16-20 minutes (mean of three experiments). The responses were effectively blocked by addition of α-D-methyl-mannopyranoside (α-D-man., 2.5%). Mean + s.e.m. of three experiments, 50 cells/experiment. Two-tailed unpaired *t*-test compared to PBS. *P* < 0.05 (*), *P* < 0.01 (**), *P* < 0.001 (***).

In depth analysis of the regulated gene sets revealed that potassium channels, ion anti-porters, voltage gated cation channels and substrate specific ion channels were up-regulated by *E*. *coli* 83972*fim* (**Fig 6A**). By kinetic analysis of consecutive samples from P I–P IV, we detected an increase in Ca^2+^ and K^+^ channel expression after 3 hours, and K^+^ channel expression was sustained, with a maximum after 48 hours, especially in P IV (**Fig 6B**). In addition, a moderate increase in Na^+^ and Cl^-^ channel expression was recorded. These gene sets were not unique for *E*. *coli* 83972*fim* but were regulated also by *E*. *coli* 83972 after 24 hours, further suggesting that type 1 fimbriae enhance the effects of *E*. *coli* 83972 (**S15 Fig**).

The activation of ion channel expression was confirmed *in vitro* in human bladder epithelial cells, representing the site of infection in human hosts. *E*. *coli* 83972*fim* and *E*. *coli* 83972 infection generated an increase in K^+^ channel protein levels, compared to uninfected control cells (TWIK, TRAAK and KCNJ11), with *E*. *coli* 83972*fim* showing the most pronounced effects (**Fig 6C**). In addition, we detected an increase in cation channel protein levels (TRPC1, TRPV6), exclusively in *E*. *coli* 83972*fim* infected cells (**Fig 6D** and **6E**). The soluble receptor analogue α-D-methyl-mannopyranoside (α-D-man., 2.5%) effectively blocked the TRPC1 and TRPV6 response, suggesting that the effects are type 1 fimbriae specific (**Fig 6F**).

This hypothesis was confirmed by treating human bladder epithelial cells with purified FimCH protein complexes (1.25–5 μg/ml), [44–47]. A dose-dependent increase in TRPC1 expression was detected (**Fig 6G** and **6H**) and the FimCH complex was shown to activate rapid Ca^2+^, K^+^ and Zn^2+^ fluxes, which were inhibited by α-D-methyl-mannopyranoside (**Fig 6I**), suggesting that type 1 fimbriae stimulate ion fluxes, in an adhesin-dependent manner. As ion fluxes regulate a variety of cellular responses, we suggest that these findings identify a general mechanism by which type 1 fimbriae may affect tissue homeostasis at different mucosal sites.

### Nerve cell related responses to type 1 fimbriae

**Fig 7:**
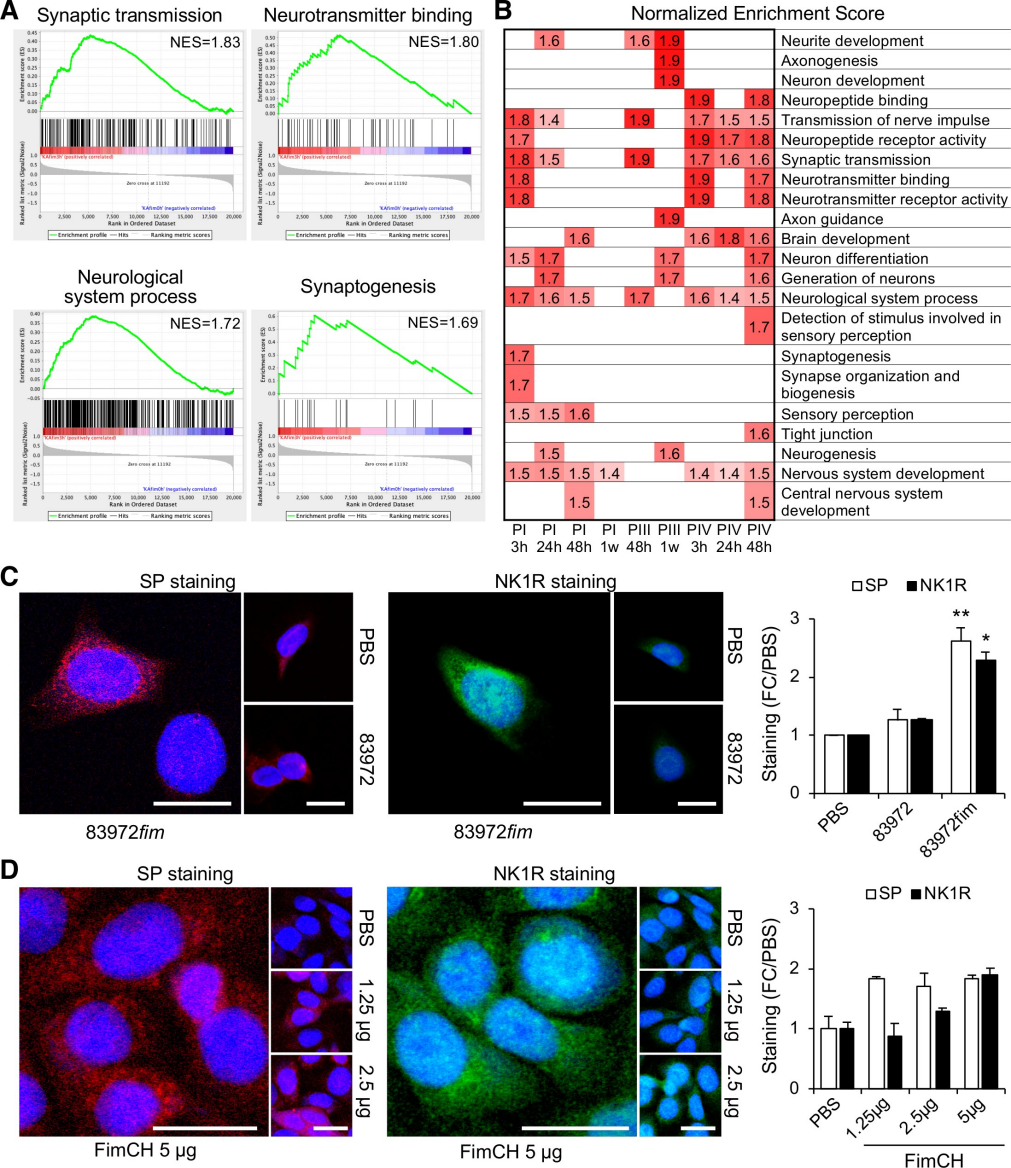
Neuronal sensing and nervous system development regulated by E. coli 83972fim. **A.** GSEA analysis identified 22 significantly regulated gene sets, involved in neuro-transmitter receptor expression and neuropeptide binding (nominal *P*-value < 0.01). These gene sets were identified compared to the pre-inoculation sample in each patient, as exemplified in P I after 3 hours. NES = normalized enrichment score. **B.** Significant NES data (*P* < 0.01) for gene-sets activated at different time points in patients inoculated with *E. coli* 83972*fim* (P I–P IV). Regulated categories included genes involved in neurotransmission, nervous system development and taste receptors. **C.** Increased levels of SP (red) and its receptor NK1R (green) in bladder epithelial cells infected with *E. coli* 83972*fim* or *E. coli* 83972 (10^5^ cfu/ml, 4 hours). **D.** The purified FimCH protein complex (1.25–5 ug/mL) stimulated SP (red) and NK1R (green) responses, as shown by confocal imaging. Mean + s.e.m. of three experiments, 50 cells/experiment. Two-tailed unpaired *t*-test compared to PBS. Scale bars = 20 μm. *P* < 0.05 (*), *P* < 0.01 (**).

Further analysis of patients inoculated with *E*. *coli* 83972*fim* identified 22 significantly activated gene sets (nominal *p*-value *≤* 0.01), involved in neuronal sensing, neurotransmitter receptor activity and nervous system development (**Fig 7A** and **7B**). Genes within these categories were regulated in all inoculated individuals but the time and amplitude of the maximum response varied between the patients (**Fig 7B**).

The response was reproduced in human bladder epithelial cells, where NK1R and SP protein levels were increased *in vitro* after *E*. *coli* 83972*fim* infection [48] (**Fig 7C**). Furthermore, the purified FimCH complex stimulated NK1R and SP expression in human bladder epithelial cells (**Fig 7D**), suggesting that type 1 fimbriae contribute to the activation of neuropeptides and neuropeptide receptors in an adhesin-dependent manner.

The results identify pronounced early effects of *E*. *coli* 83972*fim* on the host environment, with inhibition of RNA processing and translation and activation of ion channel- and neuropeptide responses. *E*. *coli* 83972*fim* enhanced both the inhibitory and activating effects of *E*. *coli* 83972, in a FimH adhesin-dependent manner, but did not significantly alter the profile of expressed genes, compared to *E*. *coli* 83972. In contrast, *E*. *coli* 83972*pap* activated host gene expression and changed the gene expression repertoire.

### Comparative analysis of upstream regulators in patients inoculated with *E*. *coli* 83972*pap* or *E*. *coli* 83972*fim*

**Fig 8:**
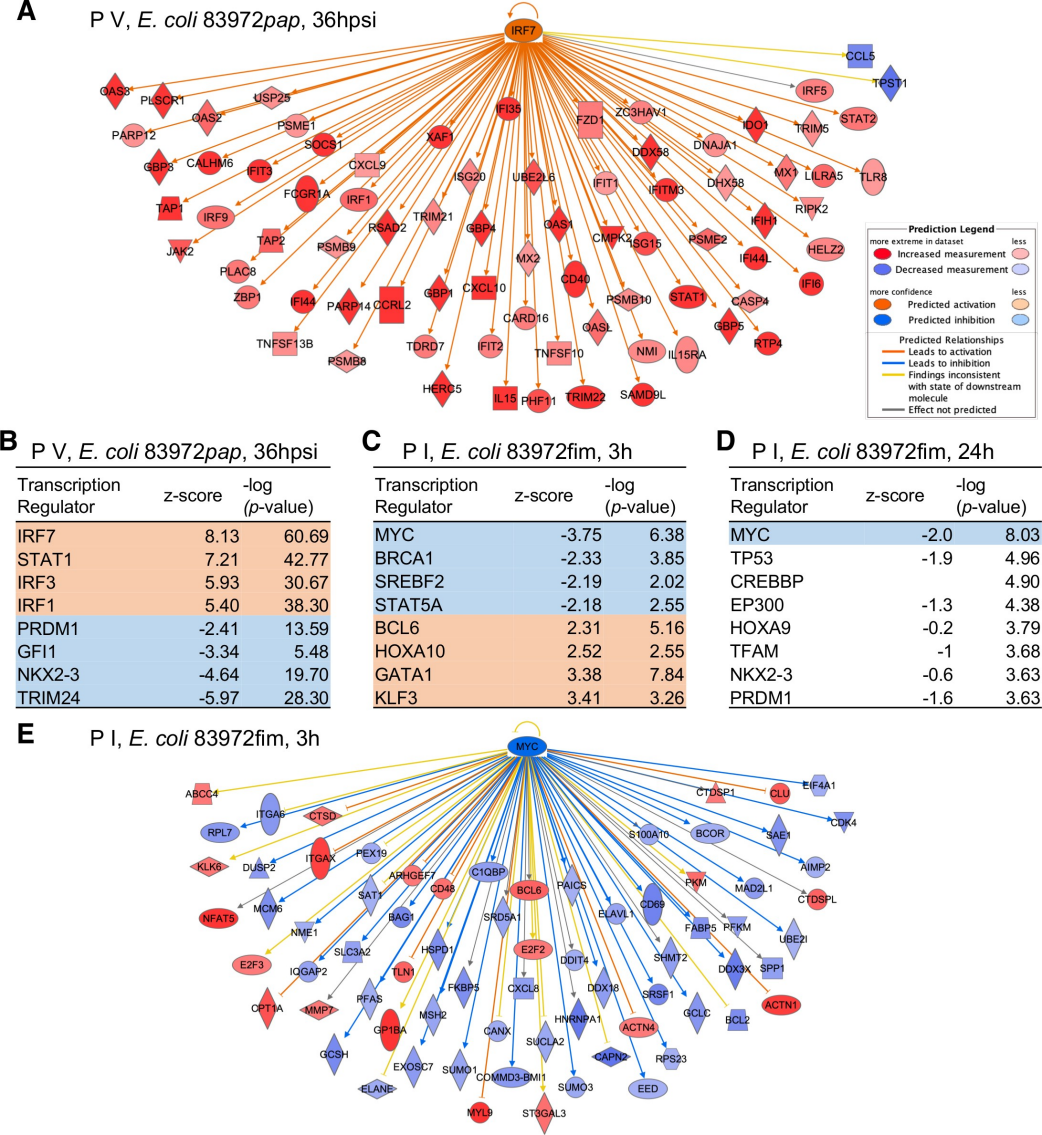
Predicted upstream regulators of transcription. Tentative upstream regulators of the transcriptional response to *E. coli* 83972*pap* and *E. coli* 83972*fim* were identified, using the IPA upstream regulator analysis. The -log (*p*-value) describes the prevalence of regulator-associated genes in data set and a positive z-score predicts activation while a negative score predicts inhibition. **A.** Prediction of IRF-7 as a transcriptional regulator of the response to *E. coli* 83972*pap* by activated downstream genes. **B.** Top predicted transcriptional regulators for the *E. coli* 83972*pap* dataset from P V at the time of symptoms red = activated, blue = inhibited). **C,D.** Predicted transcriptional regulators for *E. coli* 83972*fim* in the dataset from P I, 3 hours (**C**) or 24 hours (**D**) after inoculation. **E.** Prediction of MYC as a transcriptional regulator of the response to *E. coli* 83972*fim* by inhibited downstream genes.

Last, to provide a molecular context to these divergent effects, we identified upstream regulators of the responses to *E*. *coli* 83972*pap* or *E*. *coli* 83972*fim*, respectively. This analysis predicts key transcriptional regulators of the response, in this case to *E*. *coli* 83972*pap* or *E*. *coli* 83972*fim* (**Fig 8**). We selected the time of maximal response of each patient and fimbrial type and included all regulated genes in the respective data sets.

IRF-7 was identified as a potent upstream regulator of the response to *E*. *coli* 83972*pap*, consistent with the effects in patients and animal models of acute pyelonephritis (-log (*p*-value) = 61, **Fig 8A** and **8B**). Other identified transcriptional nodes included IRF-3, which has been shown to balance the IRF-7 response by forming heterodimeric complexes and STAT1, regulating the expression of interferon stimulated genes. *E*. *coli* 83972, in contrast, was predicted to inhibit IRF-7 and *E*. *coli* 83972*fim* had no predicted effect.

A weak, more pleiotropic pattern was detected for *E*. *coli* 83972*fim*, suggesting, that host gene expression is inhibited more broadly, by mechanisms unrelated to specific transcription factors. MYC was identified as a transcriptional regulator in P I, after 3 and 24 hours and was predicted to be inhibited, consistent with the overall inhibition of gene expression by *E*. *coli 83972fim* (-log (*p*-value) = 6.4 and 8, respectively, **Fig 8C–8E**). MYC was also predicted to be inhibited in P I by *E*. *coli* 83972 after 24 hours (-log (*p*-value) = 4) but was not regulated by *E*. *coli* 83972*pap*.

## Discussion

**Fig 9:**
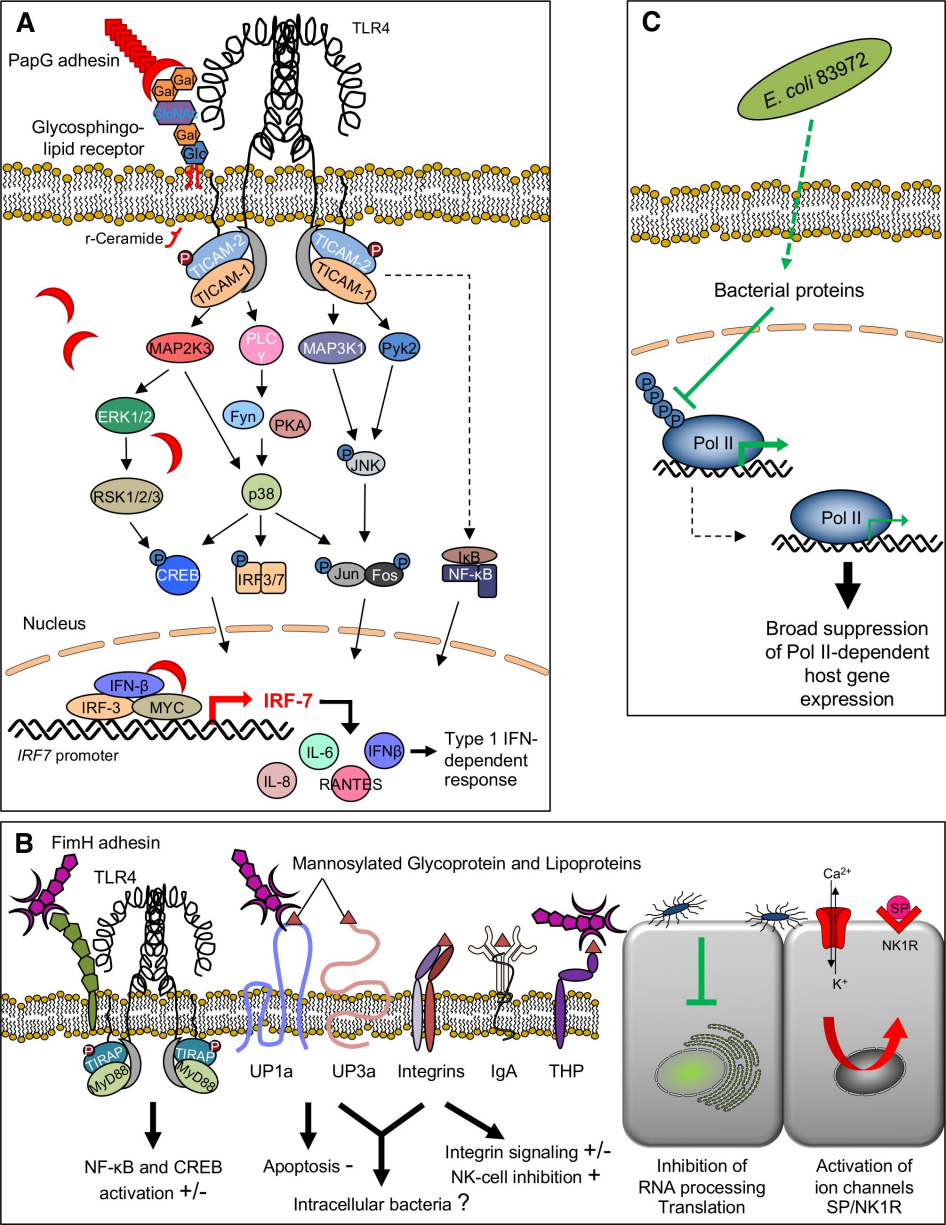
Bacterial adhesins, cell surface receptors and signaling pathways. **A.** Innate immune recognition of P-fimbriated *E. coli*. The PapG adhesin binds Galα1-4Galβ-oligosaccharide motifs in glycosphingolipid receptors. Release of ceramide, the membrane anchor of the glycolipids, activates TLR4 signaling [37, 61, 62], and phosphorylation of the TLR4 adaptor proteins TRAM (TIR domain-containing adapter molecule 2 or TICAM2) and TRIF (TIR domain-containing adapter molecule 1 or TICAM1) activates downstream signaling, involving the phosphorylation of mitogen-activated protein (MAP) kinases, phospholipase C, p38, activating JNK (c-Jun *N*-terminal kinases), CREB (cyclic AMP response element-binding) and FOS-JUN (AP1), leading to IRF3- and IRF-7-dependent and AP-1 dependent transcription of cytokine- and chemokine genes, as well as type I interferons (IFN) including IFN-β [7]. Activation results in inflammatory cell recruitment and symptoms depend on the genetic make up of the host. In this study, PapG is defined as an agonist of IRF-7, in hosts and cells inoculated with *E. coli* 83972*pap*. **B.** Type 1 fimbriae recognize several mannosylated host cell glycoconjugate receptors. The FimH adhesin binds to uroplakins [63], to integrins through *N*-oligosaccharides [64], to the Tamm-Horsfall protein (or uromodulin) [65] and to immunoglobulins [66] as well as CD48 on mucosal mast cells [67]. Downstream, type 1 fimbriae have been proposed to stimulate the innate immune response, trigger apoptosis, and stimulate mast cell degranulation as well as promoting actin rearrangement in bladder epithelial cells [2, 68, 69]. In this study, FimH is defined as a broad, mostly inhibitory regulator of RNA processing and translation and inducer of ion channel- and solute carrier expression as well as neurokinin ligand-receptor networks. **C.** ABU strains like *E. coli* 83972 have developed successful adaptation strategies, which include the deletion or inactivation of virulence genes, leading to a reduction in genome size [33]. In addition, we have shown that ABU strains actively create a calm, non-reactive environment in the host by inhibiting gene expression. An intriguing mechanism is the suppression of by RNA polymerase II (Pol II) phosphorylation, which results in a protected phenotype [23].

Bacterial pathogens have evolved sophisticated molecular strategies to colonize the appropriate host niche and adherence is an essential first step to enhance their virulence [39]. Like pathogens, commensals have evolved adhesive surface ligands to enhance their fitness, but the outcome is very different, suggesting that the quality of the adhesive interactions may distinguish commensals from pathogens (**Fig 9**). Here, we address this question by comparing P fimbriae, which are expressed by uropathogenic *E*. *coli* strains [49] to type 1 fimbriae, which are expressed among Gram-negative bacteria, with no apparent disease association. By reconstituting the *pap* or *fim* gene clusters in the non-virulent *E*. *coli* strain 83972 and inoculating human hosts with the fimbriated variants of this strain, we have had the unique opportunity to study fimbrial function and define molecular effects in human hosts.

We made the unexpected observation that the acquisition of functional P fimbriae made the ABU strain virulent in two susceptible hosts. This is explained mechanistically by bacterial reprogramming of host gene expression, including the activation of IRF-7; a transcription factor that defines tissue pathology in the murine pyelonephritis model [8]. The PapG adhesin is identified as a transcriptional *IRF7* agonist, in the context of IRF-3, IFN-β and MYC. We contrast this effect against type 1 fimbriae, which transiently inhibited genes involved in RNA processing and activated the expression of ion-channels and neuro-transmitters, with no evidence of symptoms in the host. Rather than reprogramming host gene expression, type 1 fimbriae broadly enhanced the inhibitory effects of the non-fimbriated wild type strain, suggesting a more homeostatic function. This does not exclude, however, a virulence-enhancing effect of the fimbriae, when expressed in the background of a fully virulent strain [50, 51]. The findings illustrate the remarkably divergent effects of fimbriae in the infected host.

The rationale for this study was clinical, as protective effects of *E*. *coli* 83972 inoculation have been documented, in placebo-controlled studies [29]. Fimbriae were introduced in an attempt to increase the fitness of *E*. *coli* 83972 for the urinary tract and extend the use of human inoculation therapy. There was no indication from earlier human studies that P fimbriae alone would cause a disease-like response in inoculated hosts [49, 52] and unlike fully virulent strains, *E*. *coli* 83972*pap* did not activate a disease response in the murine UTI model. UPEC-associated virulence genes are attenuated in *E*. *coli* 83972 and even after prolonged carriage further attenuation of virulence has been shown to occur, suggesting that the strains evolve towards commensalism [13]. It is important to emphasize that *E*. *coli* 83972*pap* is sensitive to antibiotics and that antibiotic therapy resulted in rapid resolution of symptoms and infection, without sequels.

These observations suggest, for the first time, that type 1 fimbriae may have potent inhibitory effects on the post-transcriptional machinery of the host, as *E*. *coli* 83972*fim* inhibited genes involved in RNA processing and translation. In addition we observed a rapid activation of K^+^ channels and solute carriers, as well as neuropeptides and their receptors, providing novel mechanistic insights into potential homeostatic effects and mechanism to broadly regulate cellular functions. Consistent with previous studies, NK cell function and integrin signaling was moderately affected by *E*. *coli* 83972*fim* (**Fig 9B**). A lack of distinct upstream transcriptional regulators suggested an entirely different level of control of the host response compared to P fimbriae, mainly executed at the post-transcriptional level. While type 1 fimbriae have been shown to increase mucosal inflammation in the murine UTI model and promote the formation of intracellular communities, type 1 fimbriae alone did not act as virulence factors in this study, when expressed in the background of a non-virulent strain.

The dual role of P fimbriae as bacterial sensors and transcriptional regulators is fascinating and challenges the dogma that virulence must rely on a complex set of virulence genes in every case. The difference between P and type 1 fimbriae further suggests that the repertoire of regulated host genes may distinguish disease-generating adhesins like P fimbriae from adhesive ligands that are involved in more homeostatic tissue functions. Based on these findings, we propose that bacteria may suffer from “virulence gene addiction”, in analogy with the “oncogene addiction” of cancer cells [53, 54]. While pathogens generally rely on multiple genes to survive in the host [55] this study suggests that a single, potent virulence determinant may be sufficient to enhance or attenuate virulence. It follows that a loss of P fimbria would represent a first step towards virulence attenuation and adaptation to long-term persistence in the urinary tract. This is supported by a high frequency of inactivating *papG* mutations in ABU isolates [13]. The findings raise the question whether therapeutic efforts should be focused on “super-virulence” gene attenuation rather than on functions that help the normal flora to maintain homeostasis in the host.

## Materials and methods

### Study participants

Five patients with recurrent lower UTI and incomplete bladder emptying were included. The patients had experienced a minimum of four symptomatic episodes/year prior to enrolment and conventional treatment, including clean intermittent catheterization (CIC), had been tried but failed (**S1 Table**). The patients had anatomically normal urinary tracts as defined by cystoscopy and CT scanning. Renal function tests were normal. All patients had incomplete voiding (residual urine between 50–300 ml; if > 100 ml treated with CIC), and had recurrent UTI. Inoculations were performed during a four-year period (October 2007—June 2011). Time between inoculations ranged between 4–13 months (median 6 months).

### Bacterial strains

*E*. *coli* 83972 (OR:K5:H–) [24] is a widely used ABU prototype strain. The genome sequence was solved in 2010, demonstrating virulence gene attenuation [13]. The *fim* gene cluster is dysfunctional due to a deletion of *fimB-fimD* and the PapG adhesin is inactivated by multiple point mutations in the *papG* coding sequence. Fimbrial expression by *E*. *coli* 83972 was re-established by cloning the intact *pap* and *fim* gene clusters from *E*. *coli* CFT073 (see details in **Supplementary Material and Methods**). *E*. *coli* 83972 reisolates from urine were identified by PCR amplification of a DNA fragment covering the deletion in the *fim* gene cluster and a fragment of the 1,565 bp cryptic plasmid specific for *E*. *coli* 83972. Urine samples were stored at -80°C. Growth characteristics of the wild type and P- or type 1 fimbriated strains were compared in LB (37°C) for 8 hours of growth. In regular 30-minute intervals, the optical density at 600 nm wavelength of the bacterial cultures was measured. For *in vitro* experiments, bacteria were cultured on tryptic soy agar plates (TSA, 16 h, 37°C), harvested in phosphate buffered saline (PBS, pH 7.2) and diluted to appropriate concentration (10^5^ cfu/ml, MOI 0.5–1) for infection.

### Human epithelial cells

The A498 human kidney carcinoma cell line from a female (A498, American Type Culture Collection #HTB-44) and the 5637 human bladder grade II carcinoma cells (5637, ATCC# HTB-9) are established models to study UTI pathogenesis [37]. Cells were cultured in RPMI-1640 supplemented with 1 mM sodium pyruvate, 1 mM non-essential amino acids, and 10% heat-inactivated fetal bovine serum (FBS) (PAA) at 37°C, 90% humidity and 5% CO_2_. For experiments, epithelial cells were cultured the previous day in six-well plates (4-6x10^5^ cells/well for Western blots and RNA extraction), or eight-well chamber slides (4-6x10^4^ cells/well for confocal imaging), (Thermo Fisher Scientific). Cells were washed and exposed to bacteria in fresh, serum-free supplemented RPMI. Cells were infected with appropriately diluted bacteria in PBS and incubated for 4 hours at 37°C with 5% CO_2_. To investigate FimH specificity, cells were treated with 2.5% α-D-methyl-mannopyranoside for 30 minutes prior to bacterial infection.

### Human therapeutic inoculation

The protocol for therapeutic bladder inoculation with *E*. *coli* 83972 has been described [30, 52]. In the present study, the protocol was modified to include only one inoculation, and this was enough for the patients to establish bacteriuria. Prior to inoculation, patients were treated with antibiotics to sterilize their urine. *E*. *coli* 83972 wild type or the fimbriated derivatives were cultured overnight (16 h) in lysogeny broth (LB), cells were harvested by centrifugation (10 min, 4,000 rpm) and re-suspended in PBS to a concentration of 10^5^ cfu/ml. Patients were inoculated with 30 ml of the solution through a catheter, which was then removed. Each patient was closely monitored. Blood and urine samples were collected prior to inoculation, three, 24 and 48 hours and at one, two, four and seven weeks after inoculation. Patients had access to a direct telephone number to the study physician at all times and were prescribed antibiotics to be used immediately in case of symptoms and upon instruction by the physician. After seven weeks the patients received antibiotic treatment, which eradicated bacteriuria in all cases.

### Monitoring of clinical parameters

To examine the effects of fimbriae on the establishment of bacteriuria and on the host response, intra-individual comparisons were performed. Three patients were first inoculated with *E*. *coli* 83972, subsequently with *E*. *coli* 83972*fim* and finally with *E*. *coli* 83972*pap* (**S1 Table**). Following inoculation, the establishment of bacteriuria was followed with repeated urine cultures and the host response was monitored by urine neutrophil counts quantified in uncentrifuged urine using a hemocytometer chamber. Interleukin-6 (IL-6) and IL-8 concentrations were quantified by Immulite (Siemens) in urine and blood. Urine samples obtained before inoculation and at each subsequent sampling point were diluted in PBS and semi-quantitatively cultured on TSA plates overnight (37°C). Prior to the inoculation the urine was sterile and neutrophil numbers, IL-6 and IL-8 concentrations were below reference values for infection.

### Fimbrial gene expression *in vivo* by qRT-PCR

Bacteria were harvested from urine samples immediately after delivery of urine samples by the patients, briefly centrifuged and resuspended immediately in RNAprotect Bacteria (Qiagen). Total RNA was extracted using the RNeasy mini kit (Qiagen) and reversely transcribed (SuperScript III, Invitrogen) in a two-step process with random hexamer primer. Prior to qPCR, the optimal annealing temperature and primer efficiency were determined. The *fimA* and *papA* transcripts were amplified using primers listed in **S3 Table**. Gene expression was quantified relative to *frr* (ribosome-recycling factor). For details, see **Supplementary methods**.

### Fimbrial function

The expression of P or type 1 fimbriae was quantified by hemagglutination. Briefly, erythrocytes were harvested from heparinized human A_1_P_1_ blood, resuspended in PBS or 2.5% α-D-methyl-mannopyranoside in PBS and mixed with bacteria on microscopy slides. Agglutination was recorded as +++, ++, + or -. Bacterial adherence to the A498 kidney epithelial cell line was assessed as previously described[9] and evaluated by differential interference contrast (DIC) microscopy (Carl Zeiss). For details, see **Supplementary methods**.

### Whole genome transcriptomic analysis

RNA was extracted from 1 ml of heparinized peripheral whole blood collected from the participants before inoculation and at seven time points after inoculation (3, 24 and 48 hours, and one, two, four and seven weeks). After purification with the QIAamp RNA Blood Mini Kit (Qiagen), 100 ng of RNA was amplified using GeneChip 3´IVT Express Kit, after fragmentation and labeling, aRNA was hybridized onto Human Genome U219 arrays (all Affymetrix) for 16 hours at 45°C, either by Aros Applied Biotechnology or in-house using the GeneAtlas system (Affymetrix). Transcriptomic data was normalized using Robust Multi Average (RMA) implemented in the Partek Express software. Fold change was calculated by comparing each sample to the pre-inoculation samples in each individual. Genes with absolute fold change >2.0 were considered differentially expressed. Heat-maps were constructed using the Gitools software. Differentially expressed genes and regulated pathways were analyzed using the Gene Set Enrichment Analysis (GSEA, Broad Institute) and the Ingenuity Pathway Analysis (IPA, Qiagen Bioinformatics) softwares. Fimbriae-specific effects on transcription were distinguished by comparing the response to *E*. *coli* 83972 inoculation at each time point and in each patient.

### Bacterial protein purification

PapDGII complexes [56] and PapGII truncated [57] were purified as previously described [41]. The PapDG protein complex was dissolved at 0.35 mg/ml in 20 mM Tris pH8.0, 100 mM NaCl. The PapGII truncate was dissolved at 0.5 mg/ml in PBS. FimCH complexes were purified as previously described [41, 58] and eluted in 65 mM NaCl. For details, see **Supplementary methods**.

### Polyclonal antibody pre-absorption

Polyclonal rabbit anti-PapGII antibody was made from native PapGII truncated protein at Sigma Biogenysis using standard protocol (Rabbit #127). An overnight culture of *E*. *coli* 83972 complemented with the plasmid pDD3 containing all *pap* genes from UPEC J96 except *papG* [31] was resuspended in PBS. 2 ml of the bacterial cells was lysed using an ultrasound sonicator (30 min at 4°C). After centrifugation, the pellet was resuspended in PBS and mixed with 1:100 of anti-PapG serum. The lysate-antibody mix was incubated for 2 h at room temperature and centrifuged. The resulting supernatant was used for experiments.

### Western blotting

After infection, cells were lysed with NP-40 lysis buffer, supplemented with protease and phosphatase inhibitors (both from Roche Diagnostics). Total cellular proteins were run on SDS–polyacrylamide gel electrophoresis (4 to 12% bis-tris gels; Invitrogen), blotted onto poly-vinylidene difluoride membranes (GE Healthcare), blocked with 5% non-fat dry milk (NFDM), and incubated with rabbit anti–IRF-7 (1:300, ab62505, Abcam, Cambridge, United Kingdom) rabbit anti-TRPC1 (1:500, #ACC-010, Alomone Labs) and rabbit anti-TRPV6 (1:500, #orb158655, Biorbyt) antibodies. The blots were washed with PBS Tween 0.1% (PBST) and incubated with HRP-linked secondary antibodies in 5% NFDM (1:4,000, goat anti-rabbit- horseradish peroxidase (HRP), #7074, Cell Signaling). The anti-β-actin (1:4,000 in 5% NFDM, #A1978, Sigma-Aldrich) followed by rabbit anti-mouse Immunoglobulins HRP-linked (1:4,000 in 5% NFDM, P0260, Dako) was used as loading control. The blots were washed with PBST and developed with ECL Plus detection reagent (GE Healthcare). Blots were imaged using the Bio-Rad ChemiDoc System (Bio-Rad) and quantification of densitometry of bands was done using the ImageJ software (NIH).

### Confocal microscopy

After infection, cells were fixed for 15 min with 3.7% formaldehyde, permeabilized with Triton X-100 (0.25% in 5% FBS/PBS) for 10 minutes and blocked with 5% FBS/PBS for 1 hour at room temperature. Primary rabbit antibodies: anti–IRF-7 antibody (1:200, ab62505, Abcam), anti-PapG pre-absorbed serum (1:1,000), anti-TWIK-1 (1:50 sc-28630, Santa Cruz biotechnologies), anti-TRAAK (1:50, sc-50413, Santa Cruz biotechnologies), anti-KCNJ2 (1:100, 3305–1, Epitomics), anti-KCNJ11 (1:100 APC-202, Alomone Labs), anti-TRPC1 (1:100, ACC-010, Alomone Labs), anti-TRPV6 (1:250 orb158655, Biorbyt) and secondary goat anti-rabbit Alexa Fluor 488–conjugated antibody (1:200, A-11034, Thermo Fisher Scientific) were used. Nuclei were stained with DRAQ-5 (ab108410, Abcam). Slides were mounted using Fluoromount and examined in a LSM 510 META laser-scanning confocal microscope (Carl Zeiss). Fluorescence was quantified using the ImageJ software.

### Host gene expression in vitro by qRT-PCR

Total RNA was extracted from cells using the RNeasy Mini Kit (Qiagen). Complementary DNA was reverse-transcribed using SuperScript III Reverse Transcriptase (Invitrogen) and oligo(dT)_20_ primers (Invitrogen). Transcripts were quantified using primer pairs against *IRF7*, *IRF3*, *OAS1*, *IFIT3*, *MYC*, *IFNB1* and *IFNA1* (all QuantiTect Primer Assay, Qiagen). Samples were run in technical and biological duplicates and *GAPDH* was used as housekeeping gene. For details, see **Supplementary methods**.

### Electrophoretic mobility shift assay

*IRF7* promoter fragment [8] was amplified from human genomic DNA (for primers see **S3 Table**) and used as probe. Each reaction contained 3–5 μL of DNA probe and 2–5 μg of cell extract from *E*. *coli* 83972*pap* infected or uninfected A498 cells in binding buffer. For the band shift/competition assay, 1–2 μg of anti–IRF-3, anti–MYC, anti-IFNβ, anti-PapGII or IgG_2A_ control were used. Binding reactions were incubated at 15°C for 30 min and loaded onto a 6% nondenaturing, nonreducing polyacrylamide gel. Alternatively, samples were loaded on a 2% agarose gel. Gels were imaged using the Bio-Rad ChemiDoc System. For details, see **Supplementary methods**.

### Cellular ion flux assays

Intracellular calcium was measured by Fluo4 NW (Molecular Probes), intracellular potassium was measured by FluxOR (Molecular Probes) according to manufacturer’s instructions in human bladder epithelial cells grown in 96-well plates (60,000 cells/well) after exposure to FimCH (5 μg). Extracellular Zn^2+^ was measured by FluoZin-3 (Thermo Fischer Scientific) by addition of 1 μg/ml of indicator salt for 60 minutes prior to FimCH treatment. Fluorescent intensity was measured by Infinite F200 (Tecan) microplate reader at 20 seconds intervals for indicated times.

### Statistical analysis

Data was examined in Prism version 6.02 (GraphPad). Normalized cytokine concentrations were compared using two-way ANOVA and Sidak’s multiple comparisons tests. Changes in pathway gene expression (fold change) after inoculation were compared intra-individually using paired t-test (two tailed p values). Staining quantifications was analyzed using unpaired t-test (two tailed *p*-values) and qRT-PCR data using multiple unpaired two-tailed Student’s *t*-test for homoscedastic variances. Results are presented as mean + s.e.m. and are representative of at least two independent experiments. Significance was accepted at *P* < 0.05 (*), *P* < 0.01 (**) or *P* < 0.001 (***). The analysis was not blinded to condition.

### Ethics statement

The study was approved by the Human Ethics Committee of the Medical Faculty, Lund University, Sweden (Dnr 298/2006; 463/2010) and informed consent forms were signed by all patients.

## Supporting information

S1 Fig Phenotyping of *E. coli* 83972*fim* and *E. coli* 83972*pap*. **A.** Functional type 1- or P fimbriae are expressed by *E*. *coli* 83972*fim* and *E*. *coli* 83972*pap* but not *E*. *coli* 83972. *E*. *coli* 83972*fim* agglutinated Guinea pig erythrocytes. *E*. *coli* 83972*pap* agglutinated human A_1_P_1_ erythrocytes. **B.** Adherence of *E*. *coli* 83972*pap* and *E*. *coli* 83972*fim* to human kidney epithelial cells (A498), *in vitro*. Light microscopy imaging, Zeiss, x100 magnification. **C.** The agglutination by *E*. *coli* 83972*fim to* human A_1_P_1_ erythrocytes was α-D-methyl-mannopyranoside (α-D-man., 2.5%) reversible. Hemagglutination of human A_1_P_1_ erythrocytes by *E*. *coli* 83972*pap* was insensitive to mannose. *E*. *coli* 83972 was hemagglutination negative in the presence or absence of α-D-methyl-mannopyranoside. **D**. *In vitro* growth rates of *E*. *coli* 83972, 83972*fim* and 83972*pap* strains. No difference was detected.
(TIF)

S2 Fig Overview of patient variables. Individual patients were inoculated on different occasions with *E*. *coli* 83972, *E*. *coli* 83972*pap* (**A**) or *E*. *coli* 83972*fim* (**B**, for details on patient characteristics see **S1 Table**). PBLs and urine samples were collected prior to inoculation and after 3, 24, 48 hours, 1, 2 and 4 weeks. Bacterial numbers (cfu/ml), PMNs (x 10^4^/ml), IL-8 (ng/l), IL-6 (ng/l) were quantified in urine at each sampling point. Fimbrial expression by reisolates was quantified by hemagglutination as +++, ++, + or–. Grey arrow = time of inoculation, red open arrow = minor symptoms, red filled arrow = symptoms requiring antibiotic treatment, psi = post symptomatic episode.
(PDF)

S3 Fig *PapA* and *fimA* expression in inoculated patients. **A.** Maps of *pap* and *fim* gene clusters defining the primers used to quantify *papA* or *fimA*. The *fimA* transcript was amplified using: forward primer (5'-taggacaggttcgtaccgcatcg-3') and reverse primer (5'-tgtccaggatctgcacaccaacg-3’). For the quantification of the *papA* transcript, forward primer (5'-tgaaacgcagtctgcaagacag-3') and reverse primer (5'-cgccaactgtttgcagcatatc-3') were used. **B.** Kinetics of *papA* fimbrial expression after human inoculation with *E*. *coli* 83972. Bacterial RNA was isolated directly from urine of each patient at the indicated time points and *papA* expression was quantified by qRT-PCR. Changes in gene expression were defined relative to *frr* (ribosome-recycling factor) expression. Value for 0h correspond to relative expression after *in vitro* growth.
(TIF)

S4 Fig Reprogramming of host gene expression by *E. coli* 83972*pap*. **A.** Rapid activation of gene expression, after inoculation with *E*. *coli* 83972*pap* (P V, 3 hours, 61% of regulated genes). A “mega-network” was generated by merging the five top-scoring expression networks detected by IPA. Major interaction nodes included MYC, NF-κB, MAPKs, IL-8 and histones. **B.** Heatmap illustrating the extent of expression reprogramming by *E*. *coli* 83972*pap*, compared to *E*. *coli* 83972 (P V, 3 hours post inoculation with either strain) and to *E*. *coli* 83972*fim* (P I, 3 hours post inoculation). **C.** Network of genes regulated 3 hours post inoculation with *E*. *coli* 83972. Significantly regulated genes were all down regulated. **D.** Heat map comparing the regulation of genes in the network at all time points tin all patients inoculated with *E*. *coli* 83972*pap*. Red = FC ≥ 2.0 and blue = FC ≤ -2.0.
(TIF)

S5 Fig P fimbriae reprogram host gene expression; *E. coli* 83972*pap* compared to *E. coli* 83972. Gene expression in P V, who developed symptoms in response to *E*. *coli* 83972*pap* after 17 days. Peripheral blood leukocytes (PBLs) were harvested before and at defined time points post inoculation. Changes in gene expression in P V after inoculation with *E*. *coli* 83972*pap* or *E*. *coli* 83972 strains. Heat maps show the patterns of upregulated (red) or downregulated (blue) genes at each time point, compared to the pre-inoculation sample in each patient (cut off FC ≥ 2.0). The corresponding Venn diagrams show the number of activated or suppressed genes in each sample and the number of genes overlapping between fimbriated strains and the wild type. Inversely regulated genes are in yellow circles. Arrows connect time-points and indicate the number of genes that remain regulated in the same patient. Black = *E*. *coli* 83972, Red = *E*. *coli* 83972*pap*.
(TIF)

S6 Fig Kinetics of gene expression in all patients after inoculation. **A-E.** Kinetics of gene regulation in five patients inoculated with *E*. *coli* 83972, *E*. *coli* 83972*pap* or *E*. *coli* 83972*fim*. Total RNA from peripheral blood leukocytes (PBLs) was used for whole genome transcriptomic analysis. Genes with an absolute Fold Change > 2.0 compared to the preinoculation sample in each patient were analyzed. Changes in gene expression in response to *E*. *coli* 83972*pap* or *E*. *coli* 83972*fim*, compared to *E*. *coli* 83972 are shown in heatmaps from each time point The corresponding numbers of activated and suppressed genes are shown in the circles. The arrows indicate the number of genes regulated throughout different time points.
(PDF)

S7 Fig Gene Set Enrichment Analysis, enrichment of regulated genes 36 hours post symptomatic episode after inoculation with *E. coli* 83972*pap* in P V. Immune response to *E*. *coli* 83972*pap* during the symptomatic episode in P V. GSEA analysis of cellular functions modified by P fimbriae expression. Significantly regulated gene sets are listed (NES = normalized enrichment score, *p*-values describe strength of enrichment compared to the pre-inoculation sample). Selected gene sets included adaptive immune genes and response to viral infection as well as antigen presentation and complement activation.
(TIF)

S8 Fig Regulation of interferon and pattern recognition pathways by *E. coli* 83972*pap*. Transcriptomic analysis of the response following *E*. *coli* 83972*pap* inoculation. **A.** Heatmaps of IFN pathway genes in P V, P II, P III and P IV, following *E*. *coli* 83972*pap* inoculation (red: ≥ 1.41 FC, blue: ≤ -1.41 FC). **B.** Regulation of the interferon signaling pathway, comparing the pathway p-value of each sample. **C.** Heatmaps of pattern recognition receptor pathway genes in P V, P II, P III and P IV (red ≥ 1.41 FC, blue ≤ -1.41 FC). **D.** Regulation of the pattern recognition receptor pathway, comparing the pathway *p*-value of each sample.
(TIF)

S9 Fig Regulation of the pathogen-specific signaling in P V at the time of symptoms. Regulation by *E*. *coli* 83972*pap* of genes in upstream and downstream of IRF-7 in P V, at the time of symptoms. Indicated genes were involved in TLR4 signaling, upstream of IRF7 and type 1 IFN responses, downstream of IRF3/IRF7. Color intensity reflects the fold change. of Red = activated; Blue = inhibited.
(TIF)

S10 Fig Similar response to *E. coli* 83972*pap* and an acute pyelonephritis strain CFT073. Gene expression in CFT073-infected human kidney epithelial cells (A498, microarray data GEO: GSE43790) was compared to the symptomatic episode in PV. A. Heatmap of significantly regulated genes in the two data sets. 115 genes were commonly regulated between PV at the time of symptoms and in vitro CFT073 (FC<2.0). In addition, 882 genes were specifically regulated in response to E. coli 83972pap and 768 genes were regulated in response to CFT073. B. The shared genes between the two data sets were used to construct the gene network shown connecting 89 genes. Gene network analysis revealed genes in the *IRF7*-dependent network, immune response and cytokine genes as well as type I interferon pathway genes. The most strongly regulated genes included *IRF7*, transcriptional regulators (*IRF1*, *RELB*, *JUN*, *ATF3*), cytokines/chemokines (*IL6*, *CXCL8*, *TNF*, *IL1B*, *CCL5*, *CXCL1*, *IL24* and *IL15*), acute phase response mediators (CRP), interferon-induced genes (*IFIH1*, *IFIT5*, *IFI44L*, *ISG20*, *IFIT2*, *IFIT3*) and cell migration and adhesion genes. The results suggest that the transcriptional reprogramming in *E*. *coli* 83972pap creates a transcriptional response that resembles the one in epithelial cells infected with fully virulent strains.
(TIF)

S11 Fig Internalization of PapG protein and Electrophoretic Mobility Shift Assay of *IRF7* promoter. **A.** PapG internalization after stimulation of cells with purified PapG and PapDG proteins (5 or 25 μg/ml). **B.** quantification of PapGII staining represented in A. Mean ± s.e.m. of at least two experiments. One-way ANOVA with Tukey’s correction compared to PBS. *P* < 0.05 (*). **C.** Electrophoretic Mobility Shift Assay (EMSA), using an amplified IRF7 promoter fragment (1563bp, -1308 to +255) mixed with nuclear proteins extract from uninfected cells and resolved by agarose gel electrophoresis. DNA-protein complex was detected as a single band shift in the gel.
(TIF)

S12 Fig Pathways regulated by *E. coli* 83972*fim*. **A**. Canonical pathway analysis at the time of maximum response in P I with *E*. *coli* 83972*fim* (3 hours). **B**. The natural killer- (NK-) cell signaling was inhibited. **C**. Heatmaps of regulated NK-cell pathway genes following *E*. *coli* 83972*fim* inoculation in all patients. **D.** Statistical analysis of NK cell pathway genes, comparing patients carrying *E*. *coli* 83972*fim* to *E*. *coli* 83972 or *E*. *coli* 83972*pap*. **E**. The Integrin signaling pathway was moderately activated. **F**. Heatmap of Integrin signaling genes, showing similar expression patterns in the two high responders (P I and P IV). **G.** Statistical analysis, comparing patients carrying *E*. *coli* 83972*fim* to *E*. *coli* 83972 or *E*. *coli* 83972*pap*. Paired t-test for each patient and time point, two-tailed values. Red: ≥ 1.41 FC, Blue: ≤ -1.41 FC.
(TIF)

S13 Fig Inhibition of host gene expression by *E. coli* 83972*fim*. **A.** Rapid inhibition of host gene expression after inoculation with *E*. *coli* 83972*fim* in P I after 3 hours (61% of regulated genes). **B.** Heat map comparing the 3 hours response to *E*. *coli* 83972*fim* in P I to *E*. *coli* 83972 from 3h to 1w. Inhibited genes were shared, with more rapid kinetics for *E*. *coli* 83972*fim* than *E*. *coli* 83972. In addition, a set of genes was specific for *E*. *coli 83972fim*. **C.** Biological processes regulated by genes in the *E*. *coli* 83972*fim* “mega network”. Top regulated functions included RNA processing, RNA translation and immune related functions. **D.** Inhibitory profile especially in P I and P IV, which lasted for at least 48 hours but was lost thereafter. Red = FC ≥ 2.0 and blue = FC ≤ -2.0.
(TIF)

S14 Fig Host gene expression response to *E. coli* 83972 or *E. coli* 83972*pap* inoculations. **A.** Rapid inhibition of gene expression, after inoculation with *E*. *coli* 83972 (P I, 3 hours). A “mega-network” was generated by merging the five top-scoring expression networks detected by IPA. **B.** Heatmap illustrating the differential effect of *E*. *coli* 83972*fim* and *E*. *coli* 83972*pap* on host gene expression. The regulation of genes in the P I, 3 hours, *E*. *coli* 83972*fim* network is shown (see **Fig 6A**). Red = FC ≥ 2.0 and blue = FC ≤ -2.0.
(TIF)

S15 Fig Gene Ontology Gene Set Enrichment Analysis (GSEA) of all patients and time points. Gene Set Enrichment Analysis was performed by analyzing gene expression compared to pre-inoculation samples of all patients and after inoculation with *E*. *coli* 83972 (**A-D**), *E*. *coli* 83972*fim* (**E-H**) or *E*. *coli* 83972*pap* (**I-L**). Enrichment plots of the 12 most strongly regulated gene sets are shown. Gene sets with False Discovery Rate <25% are considered significantly enriched.
(PDF)

S1 Table Patient characteristics and inoculation schedule. (PDF)

S2 Table Top 20 up- and down-regulated Gene ontology gene sets regulated by *E. coli* 83972*fim* after 3 hours. (PDF)

S3 Table Primers used in the study. (PDF)
