# Supplementary figures and images for: Fimbriae reprogram host gene expression – Divergent effects of P and type 1 fimbriae
Source: PLoS Pathog. 2019 Jun 10;15(6):e1007671. doi: 10.1371/journal.ppat.1007671 (PMC6557620; doi:10.1371/journal.ppat.1007671)

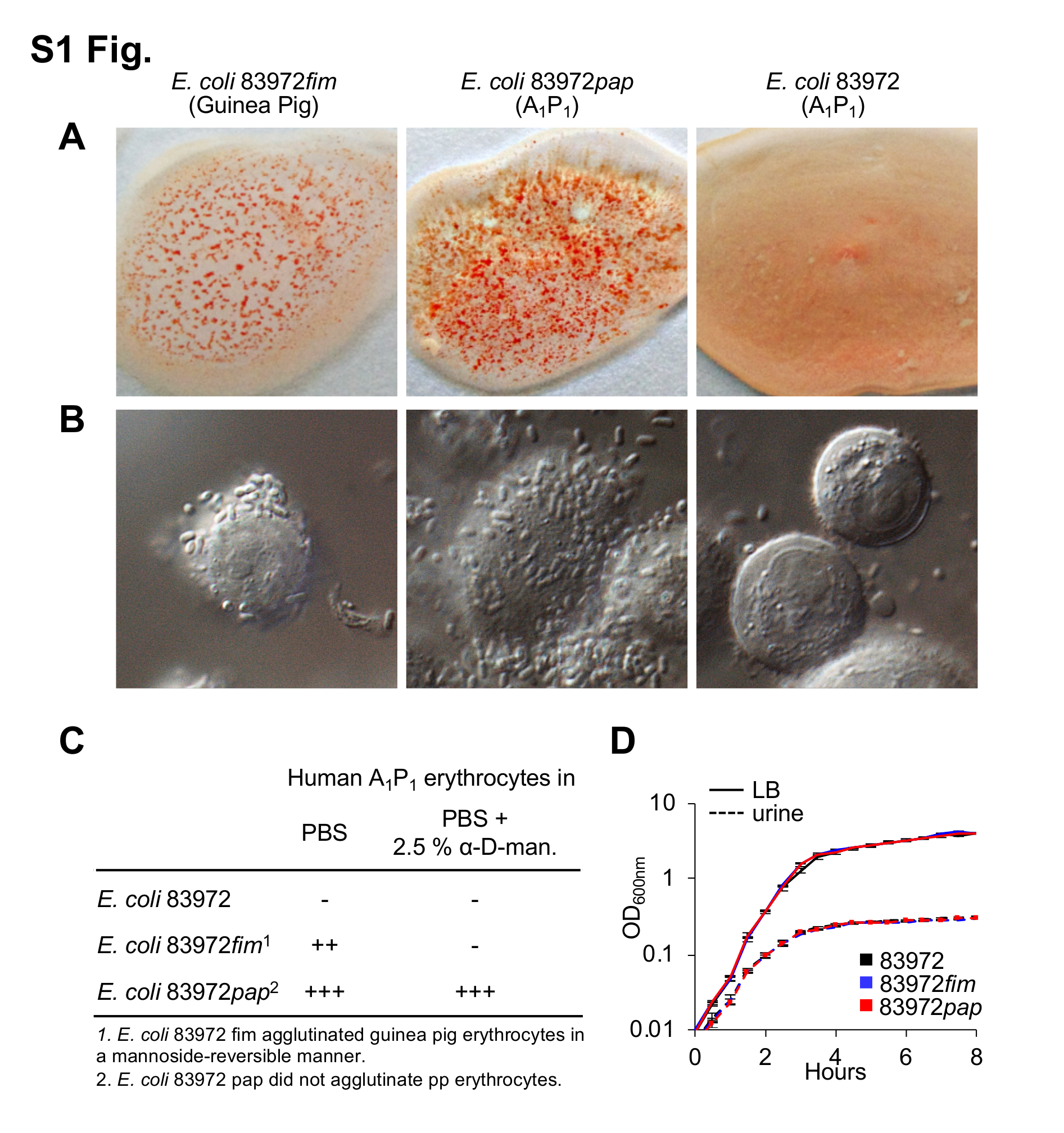

Supplement: S1 Fig — A. Functional type 1- or P fimbriae are expressed by E. coli 83972fim and E. coli 83972pap but not E. coli 83972. E. coli 83972fim agglutinated Guinea pig erythrocytes. E. coli 83972pap agglutinated human A1P1 erythrocytes. B. Adherence of E. coli 83972pap and E. coli 83972fim to human kidney epithelial cells (A498), in vitro. Light microscopy imaging, Zeiss, x100 magnification. C. The agglutination by E. coli 83972fim to human A1P1 erythrocytes was α-D-methyl-mannopyranoside (α-D-man., 2.5%) reversible. Hemagglutination of human A1P1 erythrocytes by E. coli 83972pap was insensitive to mannose. E. coli 83972 was hemagglutination negative in the presence or absence of α-D-methyl-mannopyranoside. D. In vitro growth rates of E. coli 83972, 83972fim and 83972pap strains. No difference was detected. (TIF) [file ppat.1007671.s001.tif]

**S2A Fig.**

P II

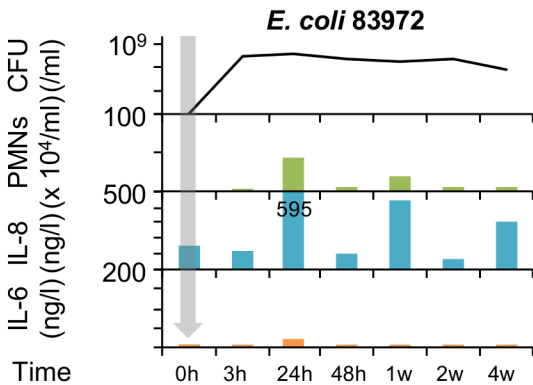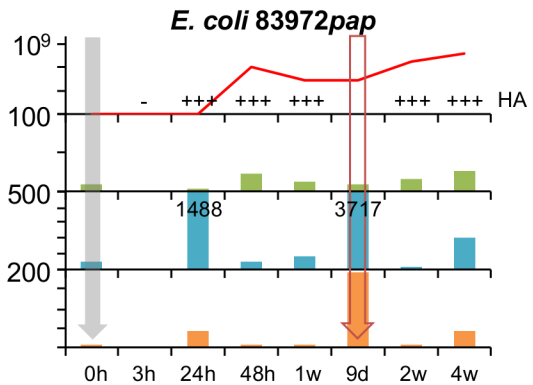

P III

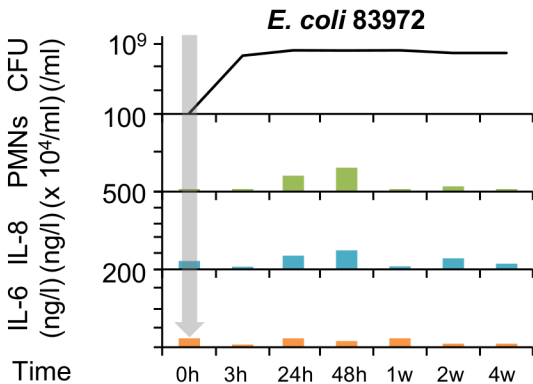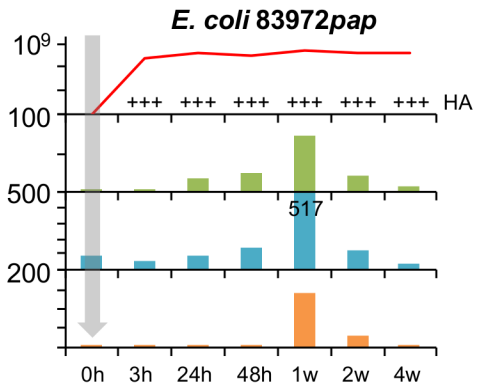

P IV

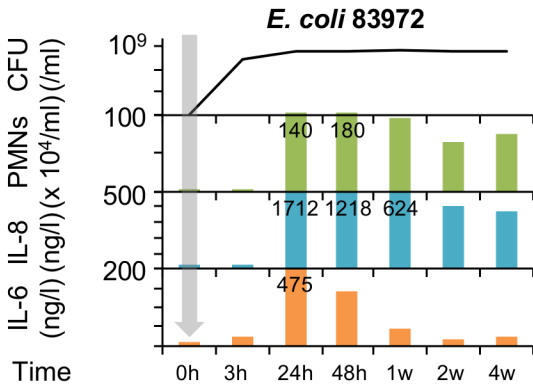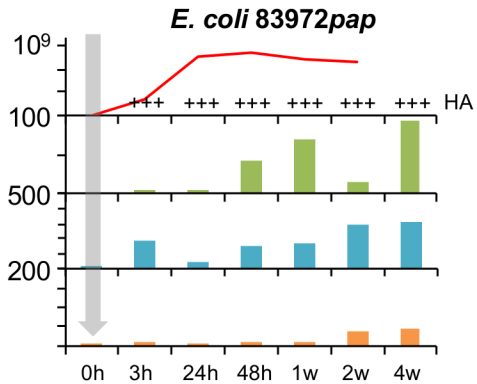

P V

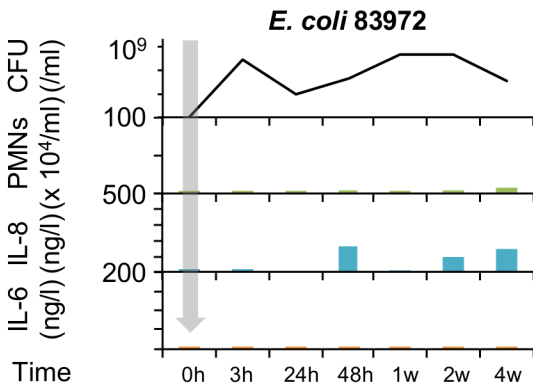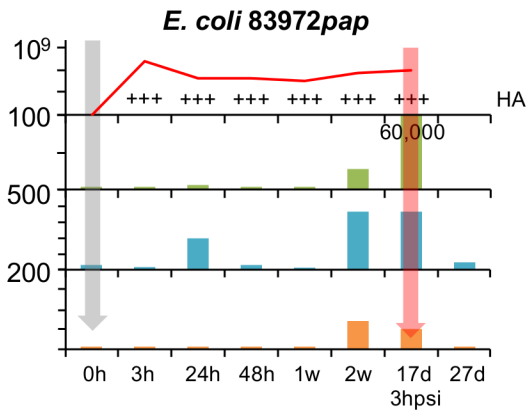

S2B Fig.

P I

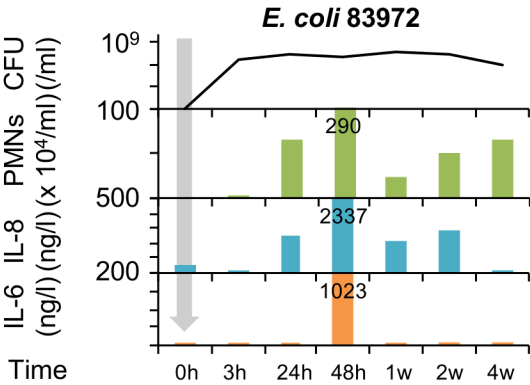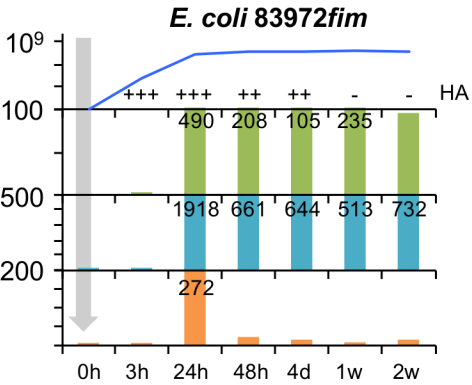

P II

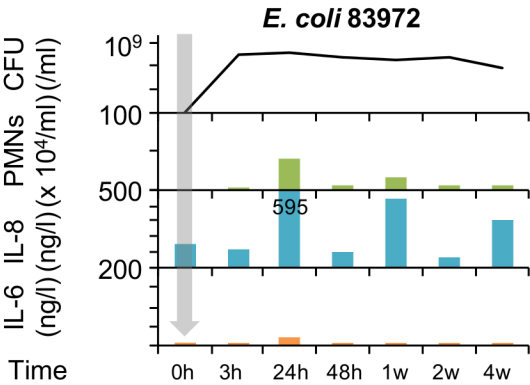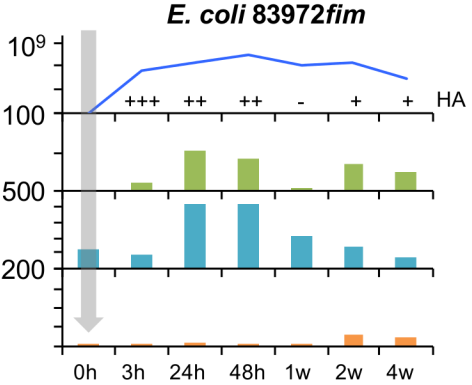

P III

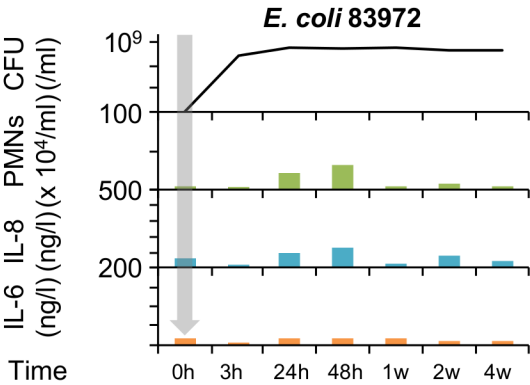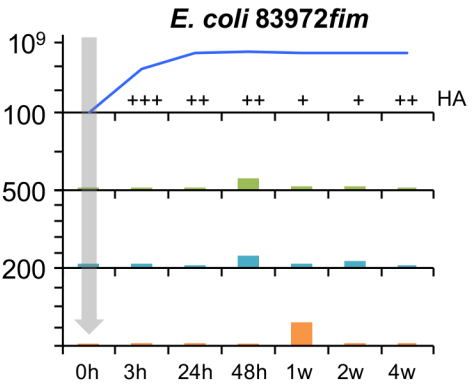

P IV

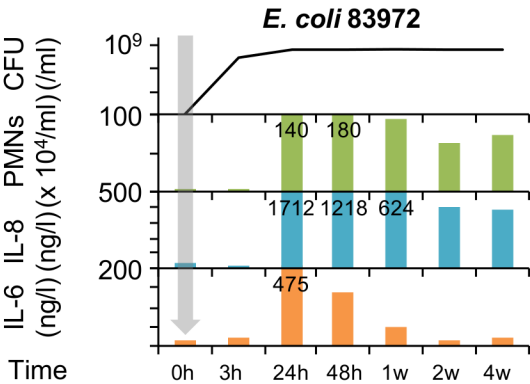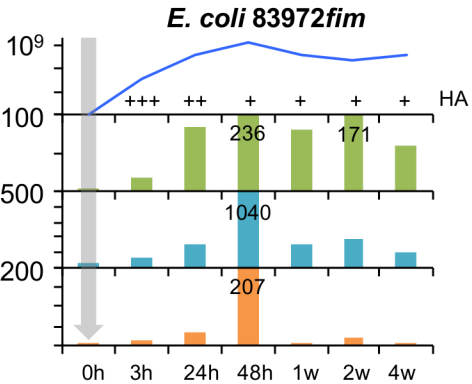

Supplement: S2 Fig — Individual patients were inoculated on different occasions with E. coli 83972, E. coli 83972pap (A) or E. coli 83972fim (B, for details on patient characteristics see S1 Table). PBLs and urine samples were collected prior to inoculation and after 3, 24, 48 hours, 1, 2 and 4 weeks. Bacterial numbers (cfu/ml), PMNs (x 104/ml), IL-8 (ng/l), IL-6 (ng/l) were quantified in urine at each sampling point. Fimbrial expression by reisolates was quantified by hemagglutination as +++, ++, + or–. Grey arrow = time of inoculation, red open arrow = minor symptoms, red filled arrow = symptoms requiring antibiotic treatment, psi = post symptomatic episode. (PDF) [file ppat.1007671.s002.pdf]

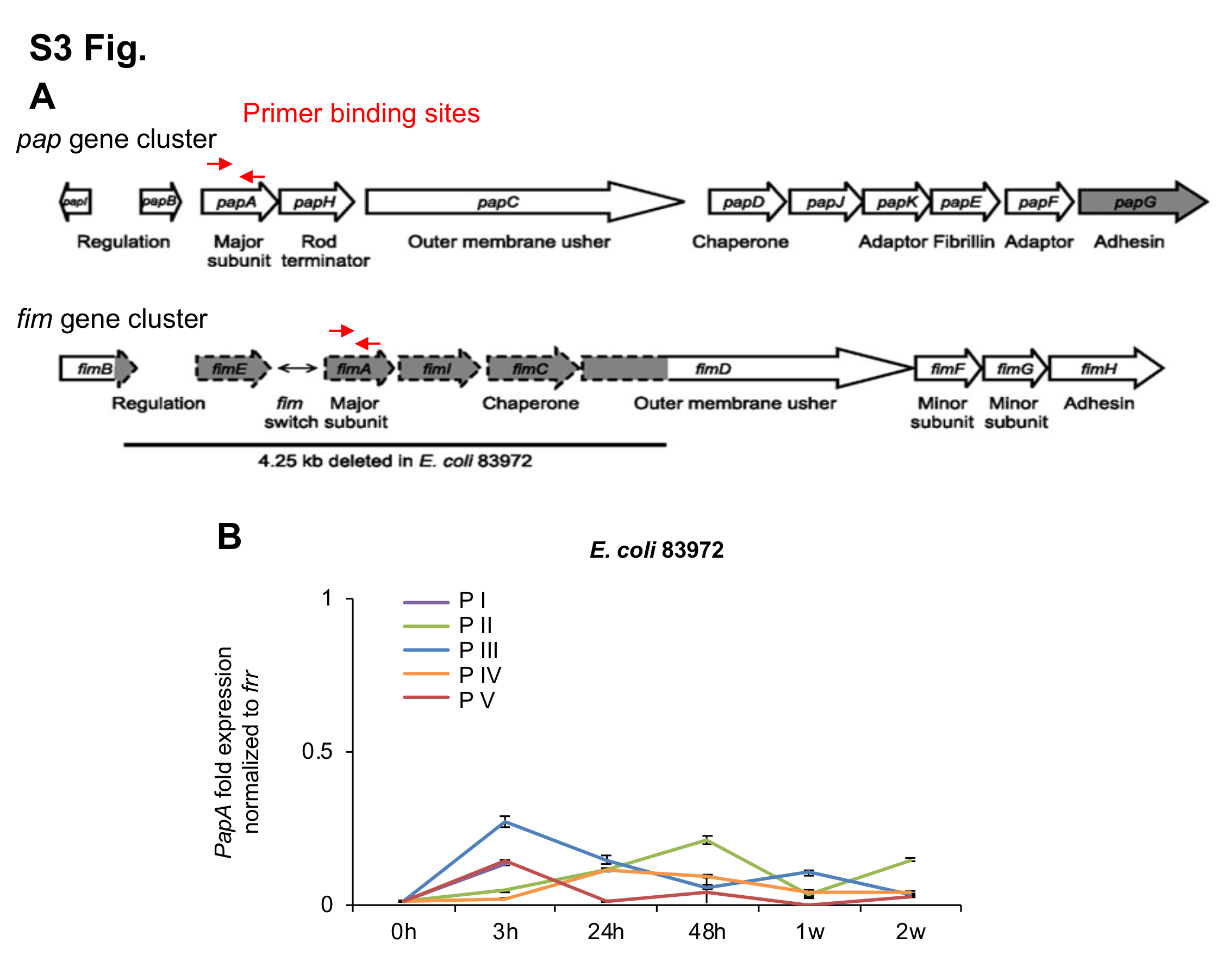

Supplement: S3 Fig — A. Maps of pap and fim gene clusters defining the primers used to quantify papA or fimA. The fimA transcript was amplified using: forward primer (5'-taggacaggttcgtaccgcatcg-3') and reverse primer (5'-tgtccaggatctgcacaccaacg-3’). For the quantification of the papA transcript, forward primer (5'-tgaaacgcagtctgcaagacag-3') and reverse primer (5'-cgccaactgtttgcagcatatc-3') were used. B. Kinetics of papA fimbrial expression after human inoculation with E. coli 83972. Bacterial RNA was isolated directly from urine of each patient at the indicated time points and papA expression was quantified by qRT-PCR. Changes in gene expression were defined relative to frr (ribosome-recycling factor) expression. Value for 0h correspond to relative expression after in vitro growth. (TIF) [file ppat.1007671.s003.tif]

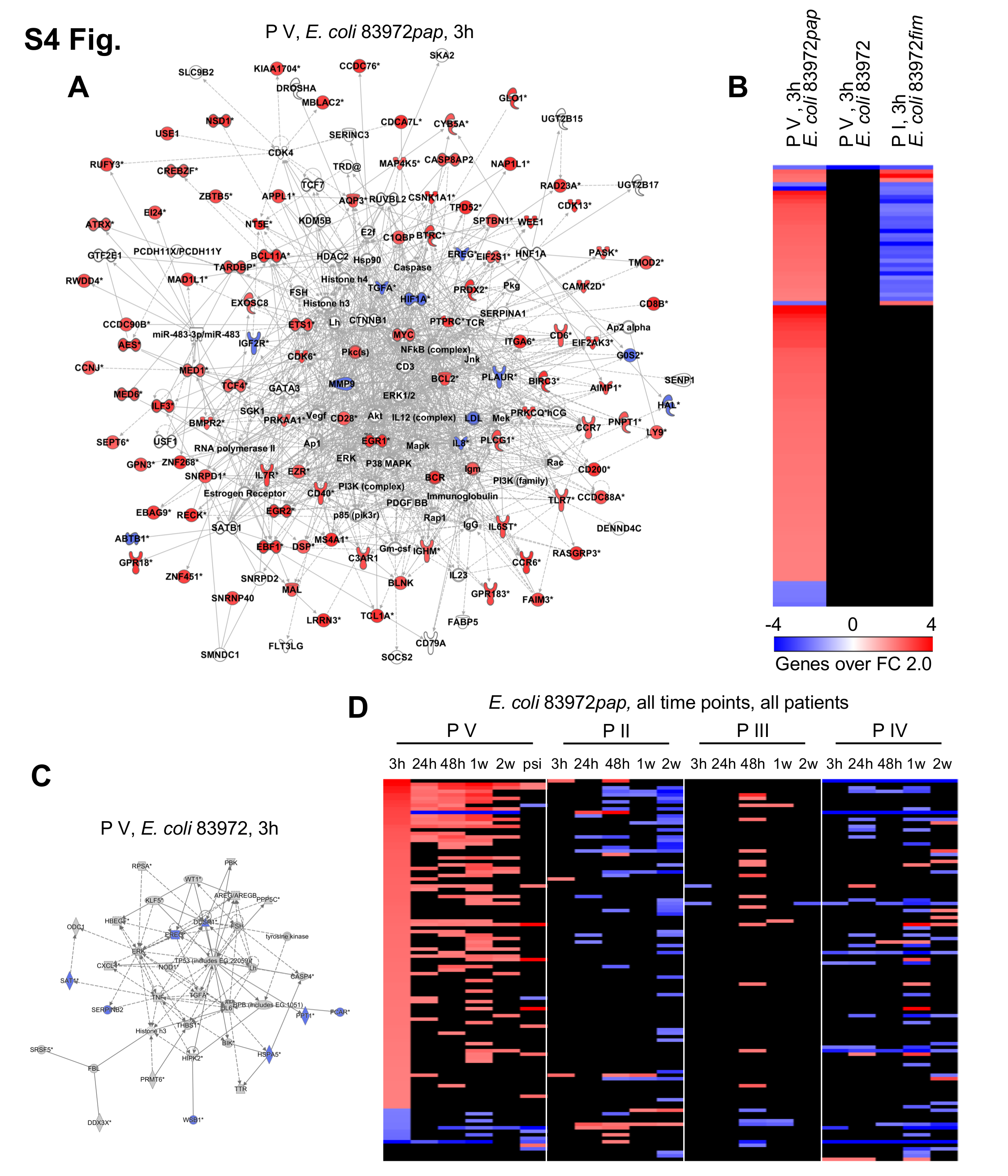

Supplement: S4 Fig — A. Rapid activation of gene expression, after inoculation with E. coli 83972pap (P V, 3 hours, 61% of regulated genes). A “mega-network” was generated by merging the five top-scoring expression networks detected by IPA. Major interaction nodes included MYC, NF-κB, MAPKs, IL-8 and histones. B. Heatmap illustrating the extent of expression reprogramming by E. coli 83972pap, compared to E. coli 83972 (P V, 3 hours post inoculation with either strain) and to E. coli 83972fim (P I, 3 hours post inoculation). C. Network of genes regulated 3 hours post inoculation with E. coli 83972. Significantly regulated genes were all down regulated. D. Heat map comparing the regulation of genes in the network at all time points tin all patients inoculated with E. coli 83972pap. Red = FC ≥ 2.0 and blue = FC ≤ -2.0. (TIF) [file ppat.1007671.s004.tif]

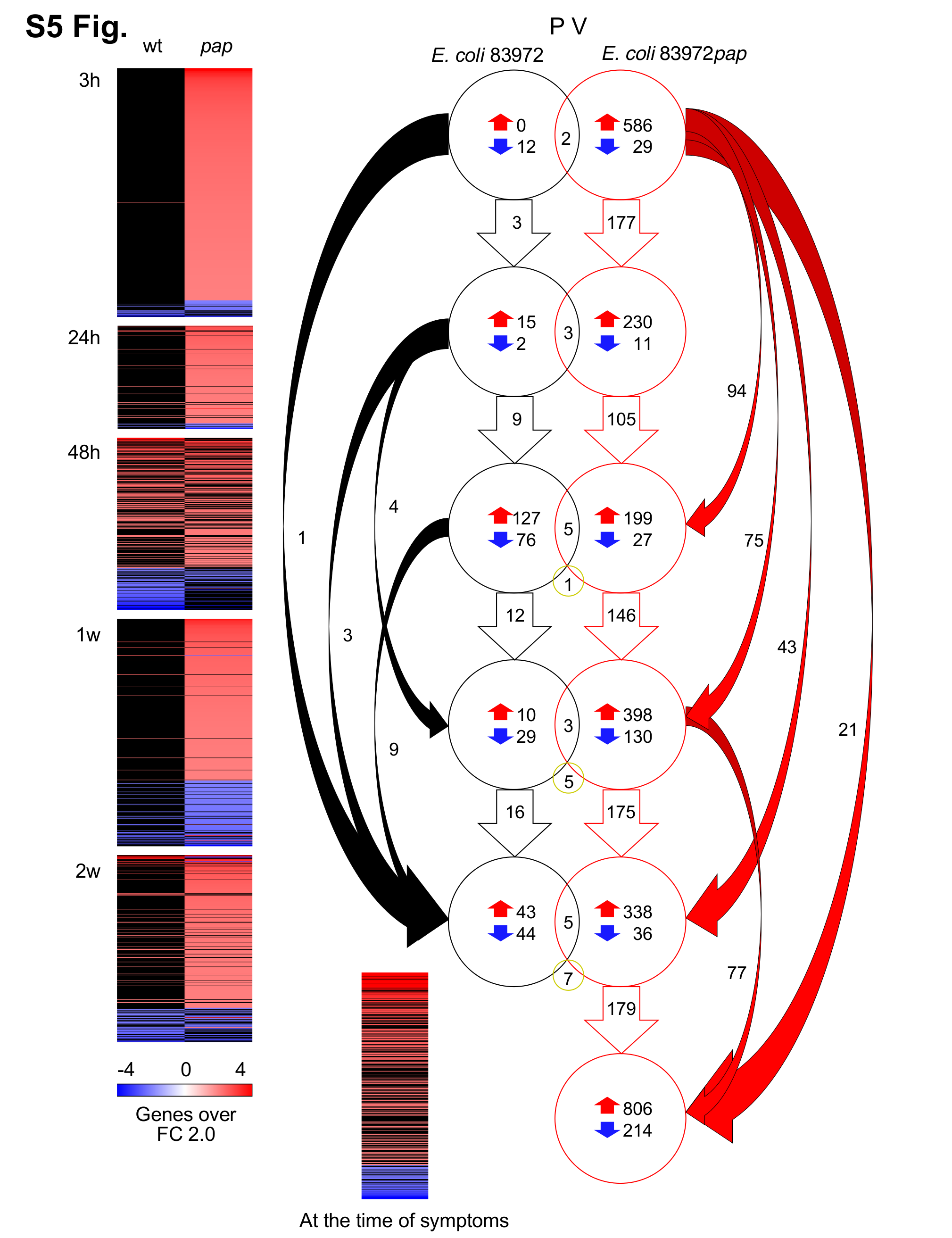

Supplement: S5 Fig — Gene expression in P V, who developed symptoms in response to E. coli 83972pap after 17 days. Peripheral blood leukocytes (PBLs) were harvested before and at defined time points post inoculation. Changes in gene expression in P V after inoculation with E. coli 83972pap or E. coli 83972 strains. Heat maps show the patterns of upregulated (red) or downregulated (blue) genes at each time point, compared to the pre-inoculation sample in each patient (cut off FC ≥ 2.0). The corresponding Venn diagrams show the number of activated or suppressed genes in each sample and the number of genes overlapping between fimbriated strains and the wild type. Inversely regulated genes are in yellow circles. Arrows connect time-points and indicate the number of genes that remain regulated in the same patient. Black = E. coli 83972, Red = E. coli 83972pap. (TIF) [file ppat.1007671.s005.tif]

**S6A Fig. 3h**

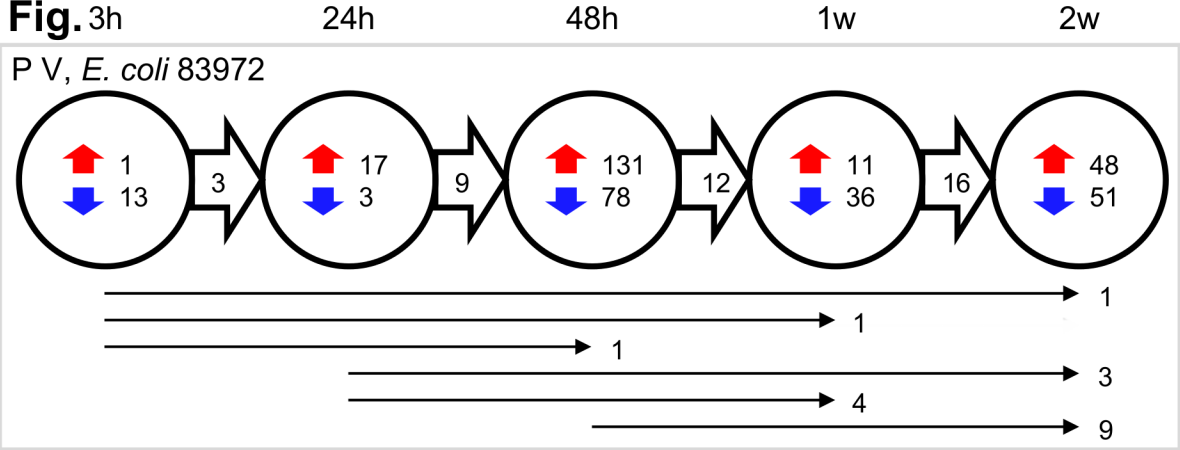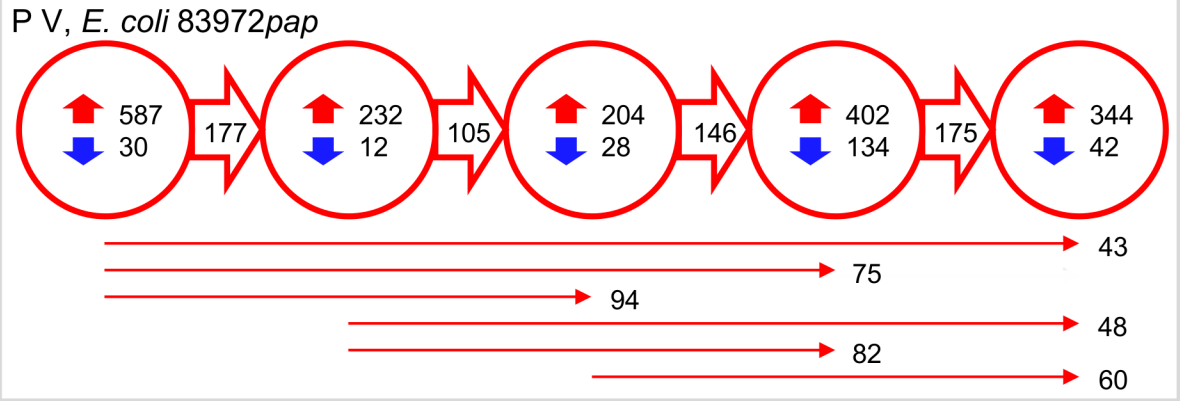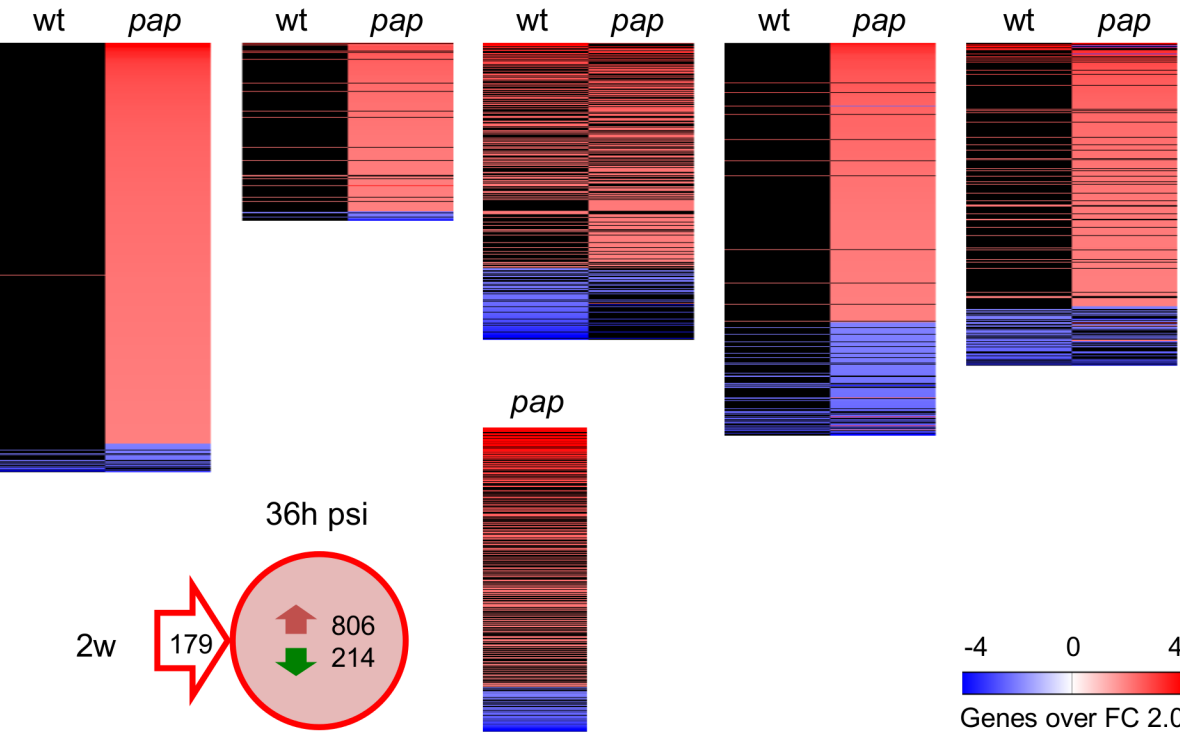

S6B Fig.3h

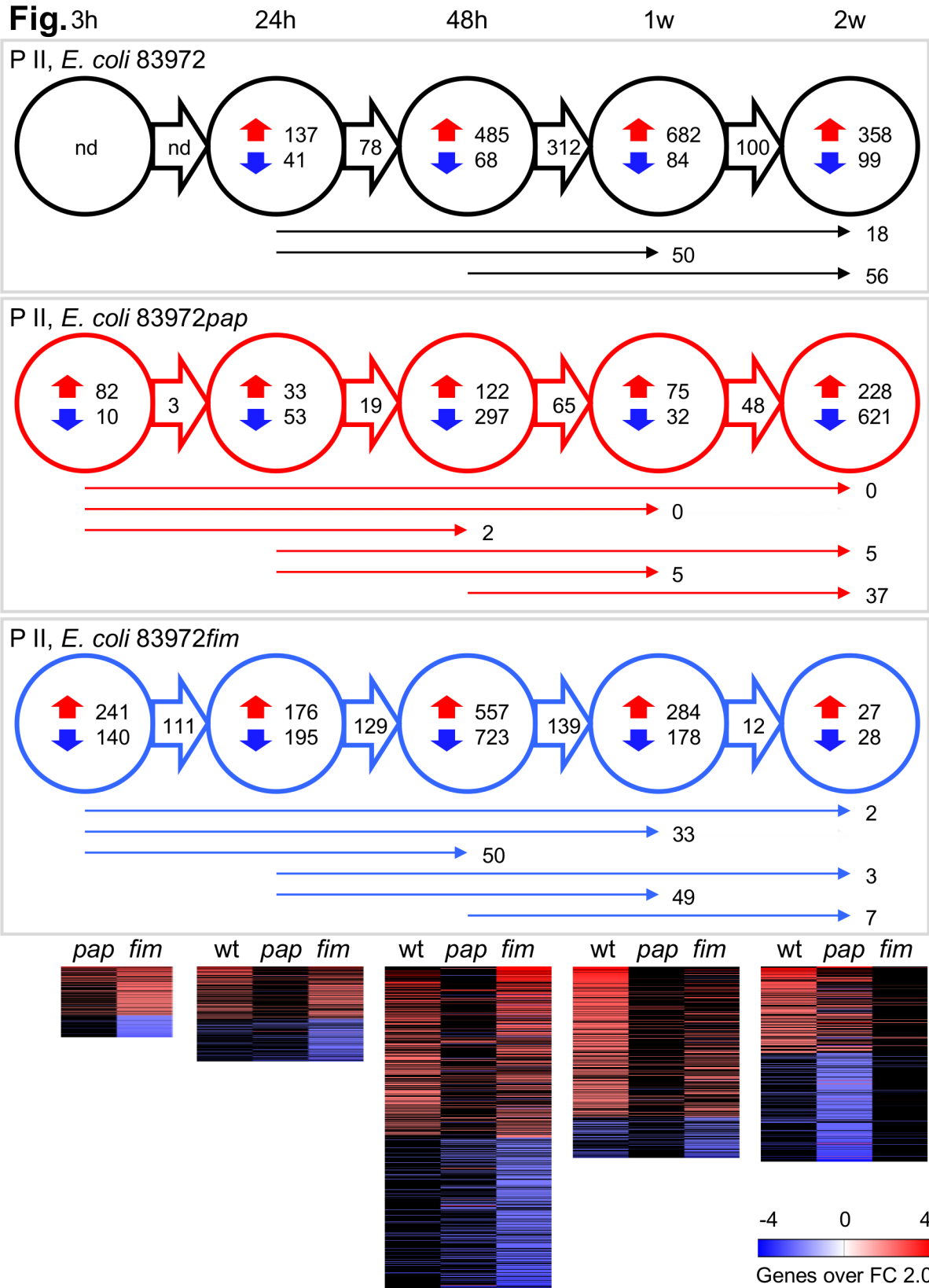

S6C Fig.3h

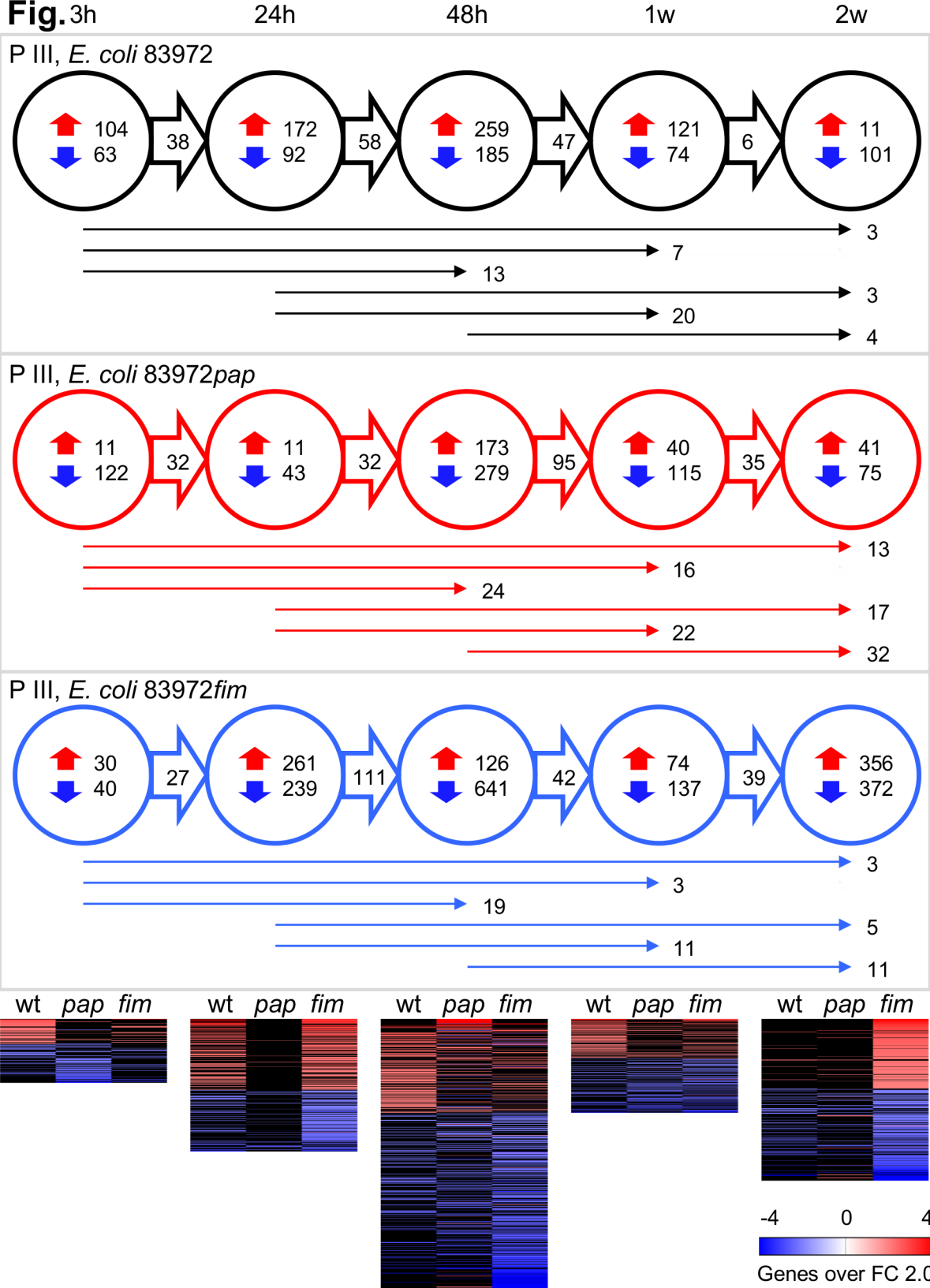

**S6D Fig.3h**

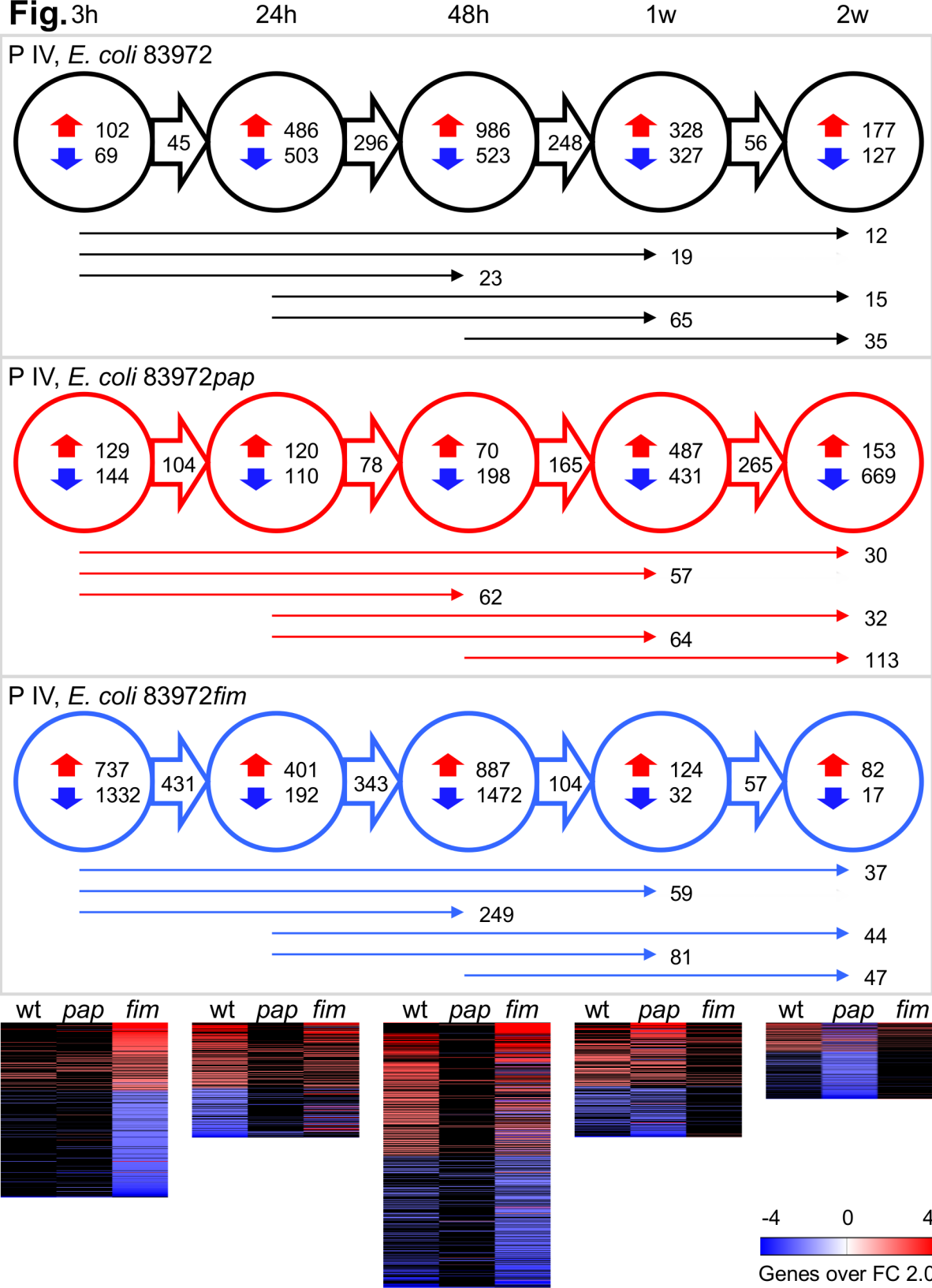

# S6E Fig. 3h

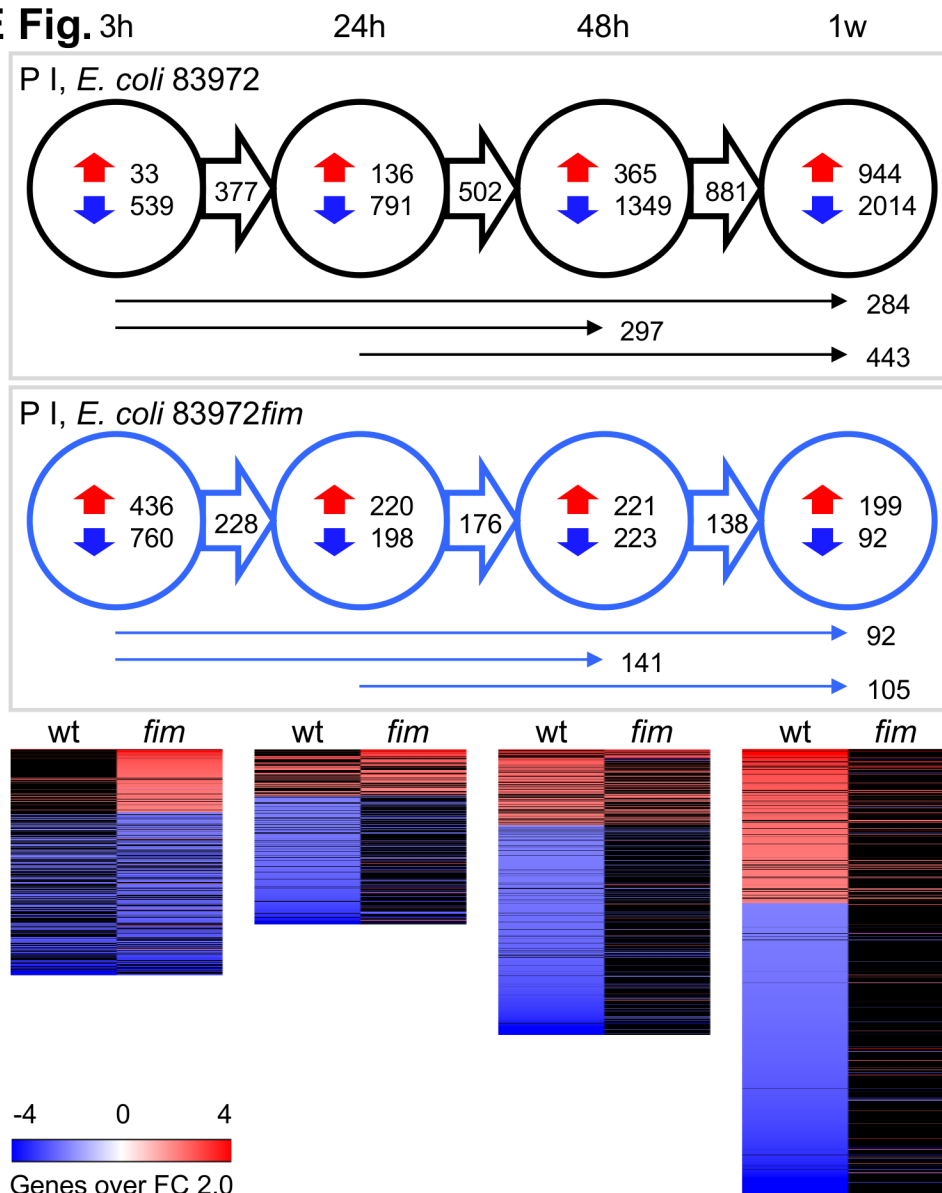

Supplement: S6 Fig — A-E. Kinetics of gene regulation in five patients inoculated with E. coli 83972, E. coli 83972pap or E. coli 83972fim. Total RNA from peripheral blood leukocytes (PBLs) was used for whole genome transcriptomic analysis. Genes with an absolute Fold Change > 2.0 compared to the preinoculation sample in each patient were analyzed. Changes in gene expression in response to E. coli 83972pap or E. coli 83972fim, compared to E. coli 83972 are shown in heatmaps from each time point The corresponding numbers of activated and suppressed genes are shown in the circles. The arrows indicate the number of genes regulated throughout different time points. (PDF) [file ppat.1007671.s006.pdf]

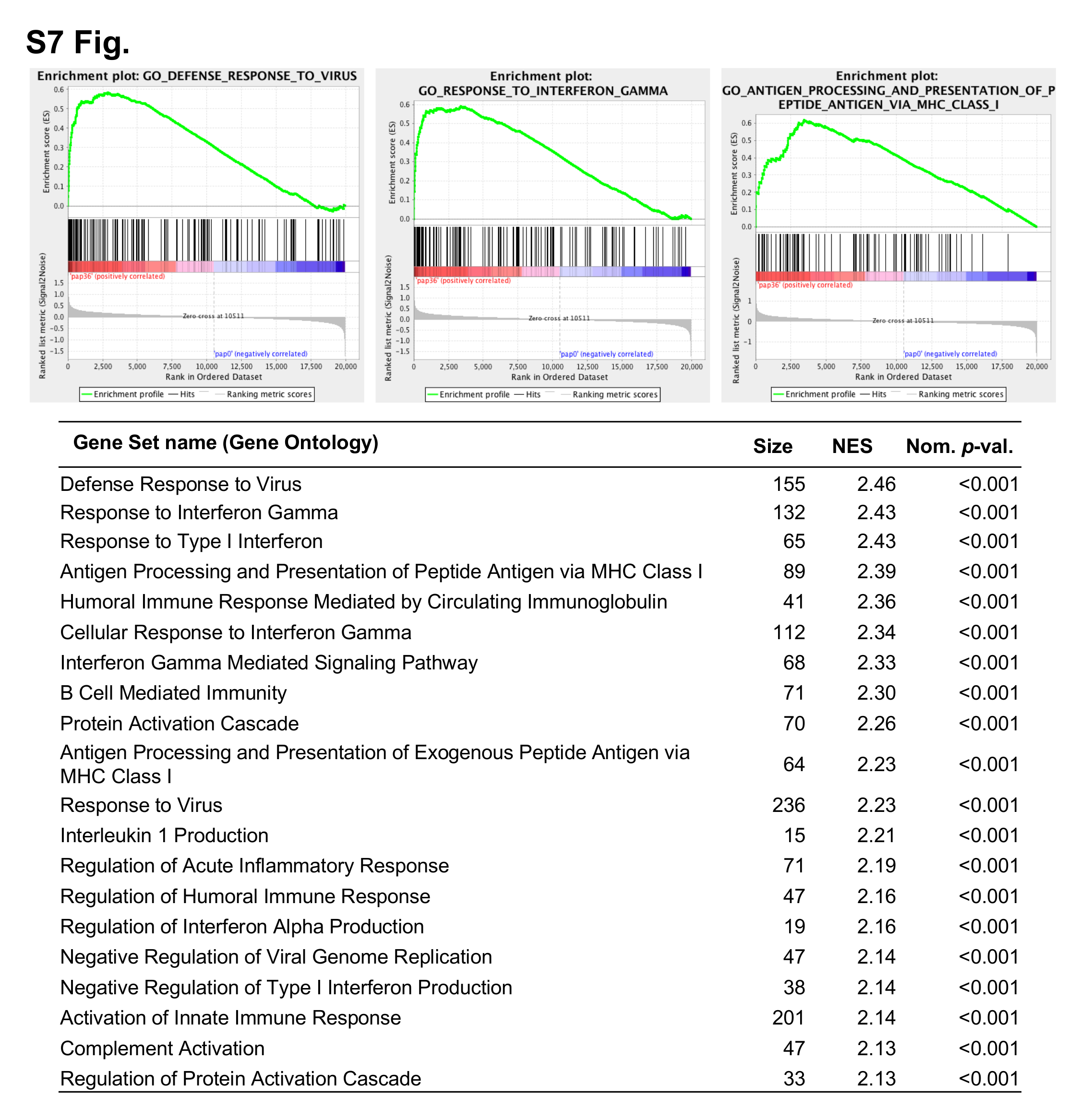

Supplement: S7 Fig — Immune response to E. coli 83972pap during the symptomatic episode in P V. GSEA analysis of cellular functions modified by P fimbriae expression. Significantly regulated gene sets are listed (NES = normalized enrichment score, p-values describe strength of enrichment compared to the pre-inoculation sample). Selected gene sets included adaptive immune genes and response to viral infection as well as antigen presentation and complement activation. (TIF) [file ppat.1007671.s007.tif]

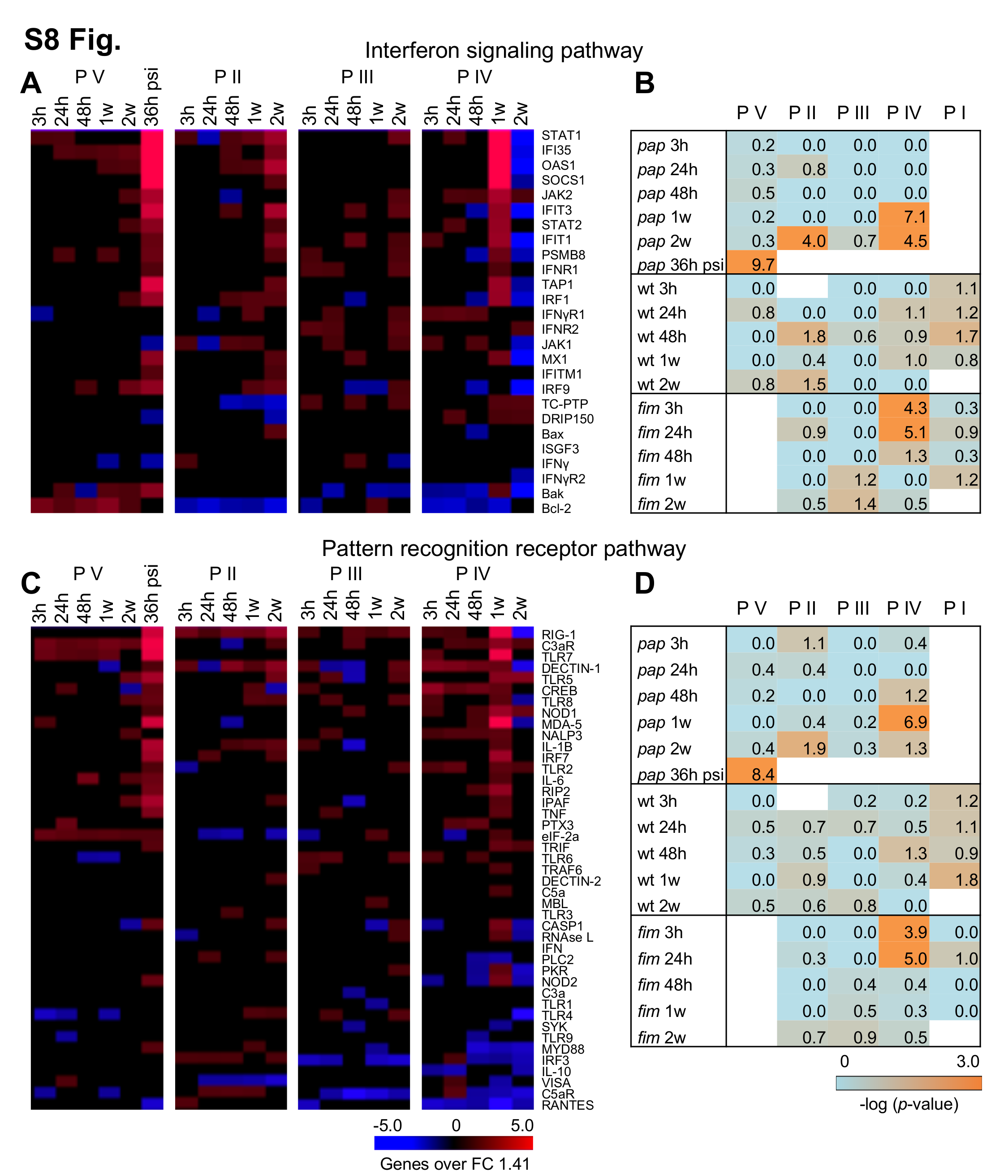

Supplement: S8 Fig — Transcriptomic analysis of the response following E. coli 83972pap inoculation. A. Heatmaps of IFN pathway genes in P V, P II, P III and P IV, following E. coli 83972pap inoculation (red: ≥ 1.41 FC, blue: ≤ -1.41 FC). B. Regulation of the interferon signaling pathway, comparing the pathway p-value of each sample. C. Heatmaps of pattern recognition receptor pathway genes in P V, P II, P III and P IV (red ≥ 1.41 FC, blue ≤ -1.41 FC). D. Regulation of the pattern recognition receptor pathway, comparing the pathway p-value of each sample. (TIF) [file ppat.1007671.s008.tif]

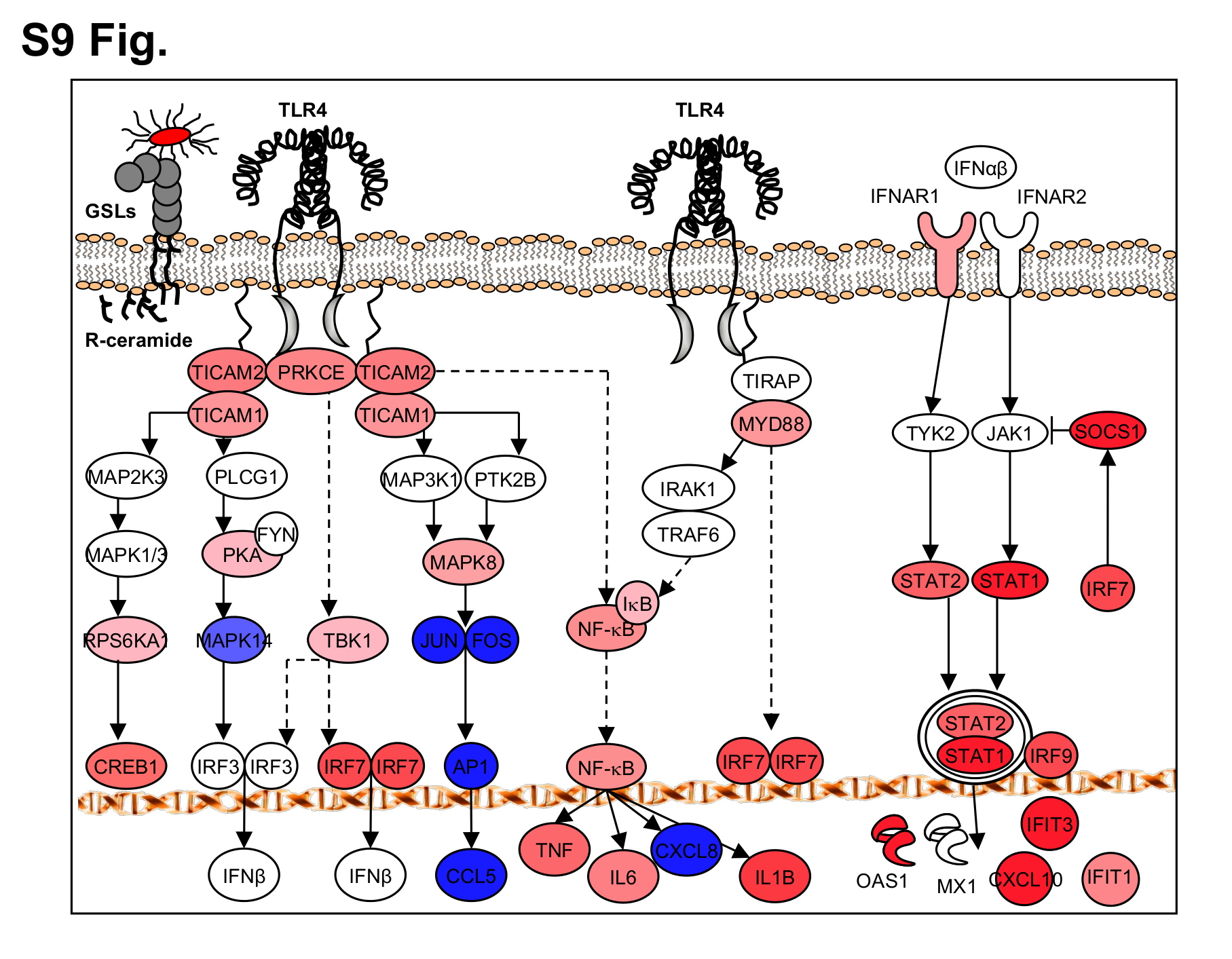

Supplement: S9 Fig — Regulation by E. coli 83972pap of genes in upstream and downstream of IRF-7 in P V, at the time of symptoms. Indicated genes were involved in TLR4 signaling, upstream of IRF7 and type 1 IFN responses, downstream of IRF3/IRF7. Color intensity reflects the fold change. of Red = activated; Blue = inhibited. (TIF) [file ppat.1007671.s009.tif]

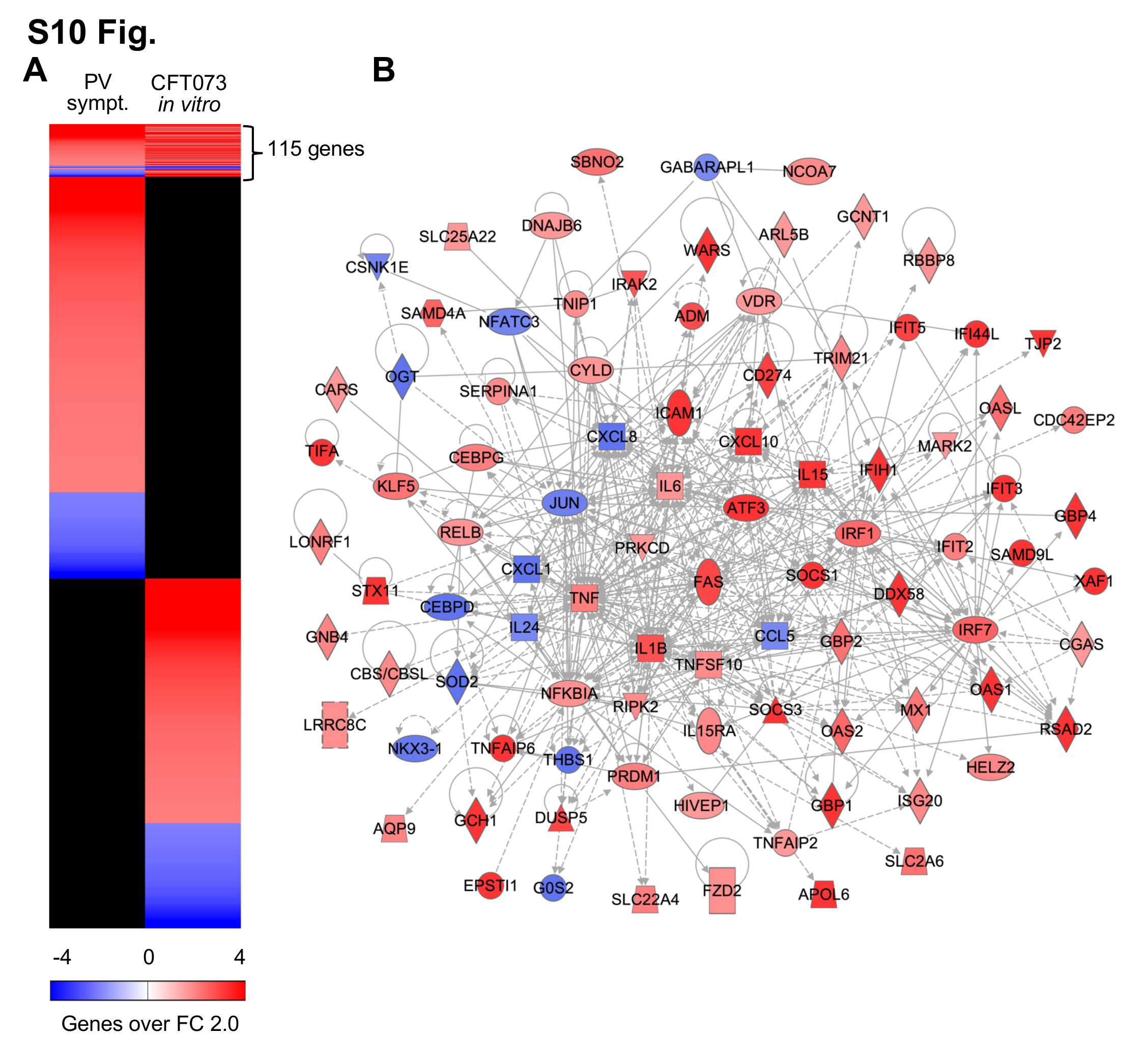

Supplement: S10 Fig — Gene expression in CFT073-infected human kidney epithelial cells (A498, microarray data GEO: GSE43790) was compared to the symptomatic episode in PV. A. Heatmap of significantly regulated genes in the two data sets. 115 genes were commonly regulated between PV at the time of symptoms and in vitro CFT073 (FC<2.0). In addition, 882 genes were specifically regulated in response to E. coli 83972pap and 768 genes were regulated in response to CFT073. B. The shared genes between the two data sets were used to construct the gene network shown connecting 89 genes. Gene network analysis revealed genes in the IRF7-dependent network, immune response and cytokine genes as well as type I interferon pathway genes. The most strongly regulated genes included IRF7, transcriptional regulators (IRF1, RELB, JUN, ATF3), cytokines/chemokines (IL6, CXCL8, TNF, IL1B, CCL5, CXCL1, IL24 and IL15), acute phase response mediators (CRP), interferon-induced genes (IFIH1, IFIT5, IFI44L, ISG20, IFIT2, IFIT3) and cell migration and adhesion genes. The results suggest that the transcriptional reprogramming in E. coli 83972pap creates a transcriptional response that resembles the one in epithelial cells infected with fully virulent strains. (TIF) [file ppat.1007671.s010.tif]

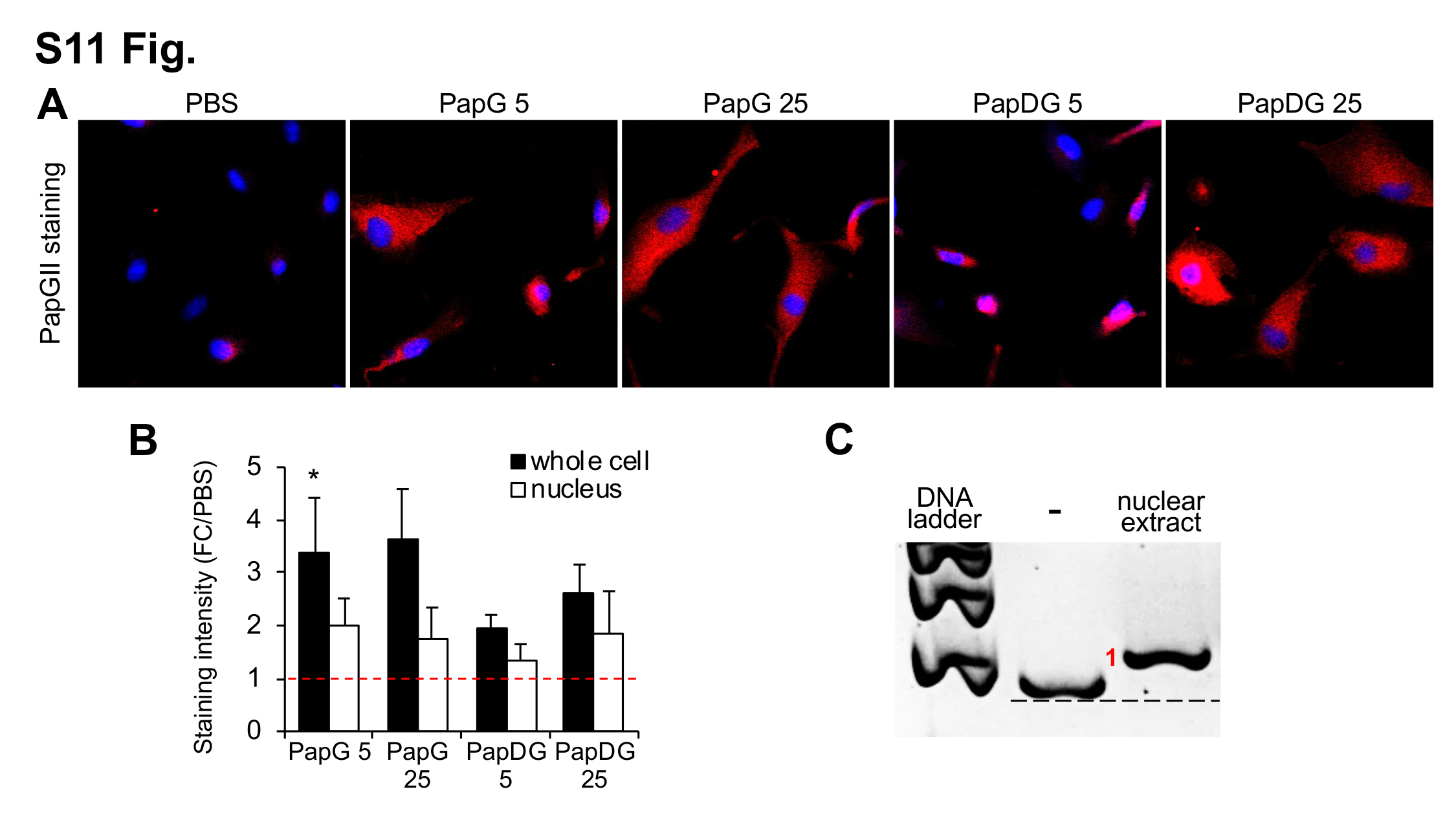

Supplement: S11 Fig — A. PapG internalization after stimulation of cells with purified PapG and PapDG proteins (5 or 25 μg/ml). B. quantification of PapGII staining represented in A. Mean ± s.e.m. of at least two experiments. One-way ANOVA with Tukey’s correction compared to PBS. P < 0.05 (*). C. Electrophoretic Mobility Shift Assay (EMSA), using an amplified IRF7 promoter fragment (1563bp, -1308 to +255) mixed with nuclear proteins extract from uninfected cells and resolved by agarose gel electrophoresis. DNA-protein complex was detected as a single band shift in the gel. (TIF) [file ppat.1007671.s011.tif]

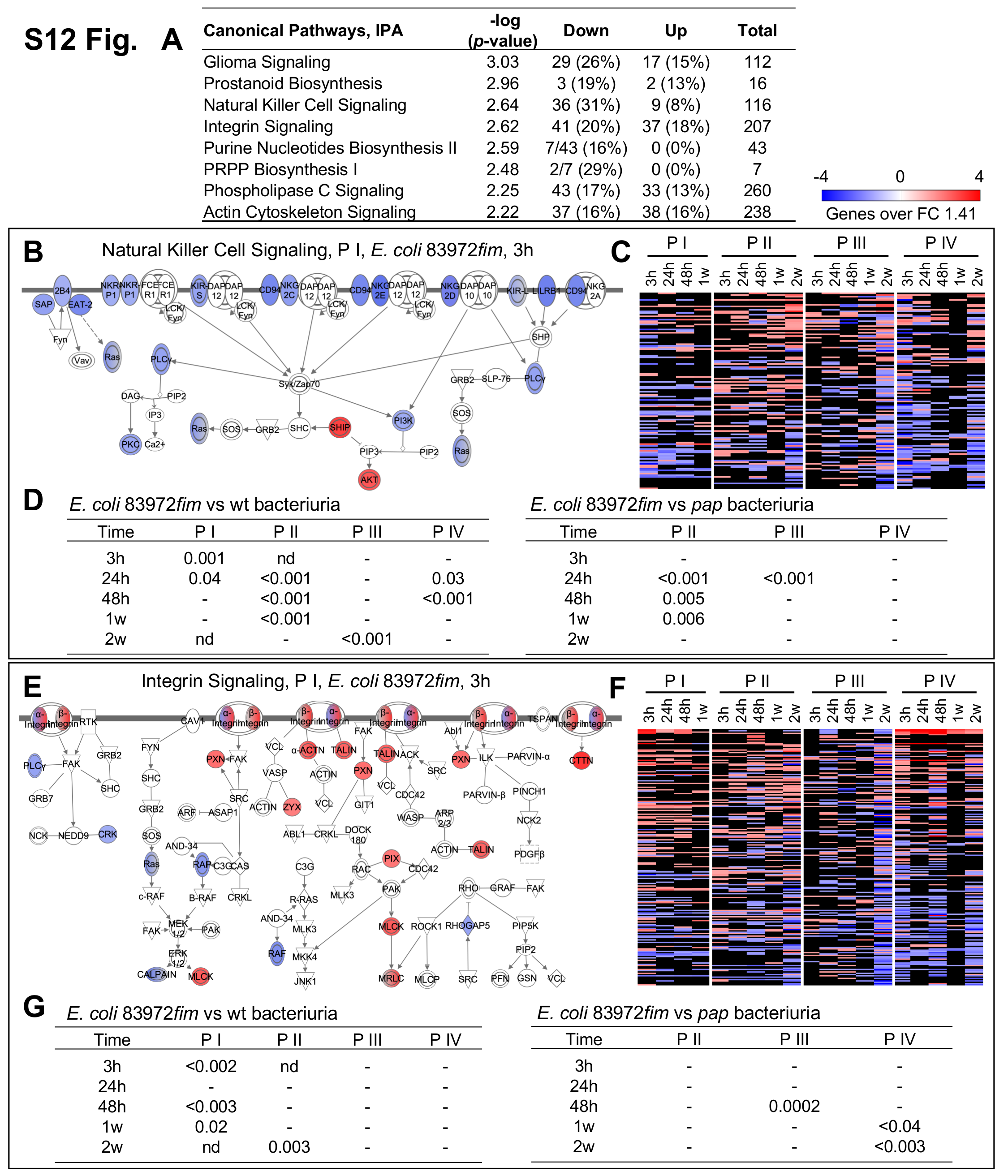

Supplement: S12 Fig — A. Canonical pathway analysis at the time of maximum response in P I with E. coli 83972fim (3 hours). B. The natural killer- (NK-) cell signaling was inhibited. C. Heatmaps of regulated NK-cell pathway genes following E. coli 83972fim inoculation in all patients. D. Statistical analysis of NK cell pathway genes, comparing patients carrying E. coli 83972fim to E. coli 83972 or E. coli 83972pap. E. The Integrin signaling pathway was moderately activated. F. Heatmap of Integrin signaling genes, showing similar expression patterns in the two high responders (P I and P IV). G. Statistical analysis, comparing patients carrying E. coli 83972fim to E. coli 83972 or E. coli 83972pap. Paired t-test for each patient and time point, two-tailed values. Red: ≥ 1.41 FC, Blue: ≤ -1.41 FC. (TIF) [file ppat.1007671.s012.tif]

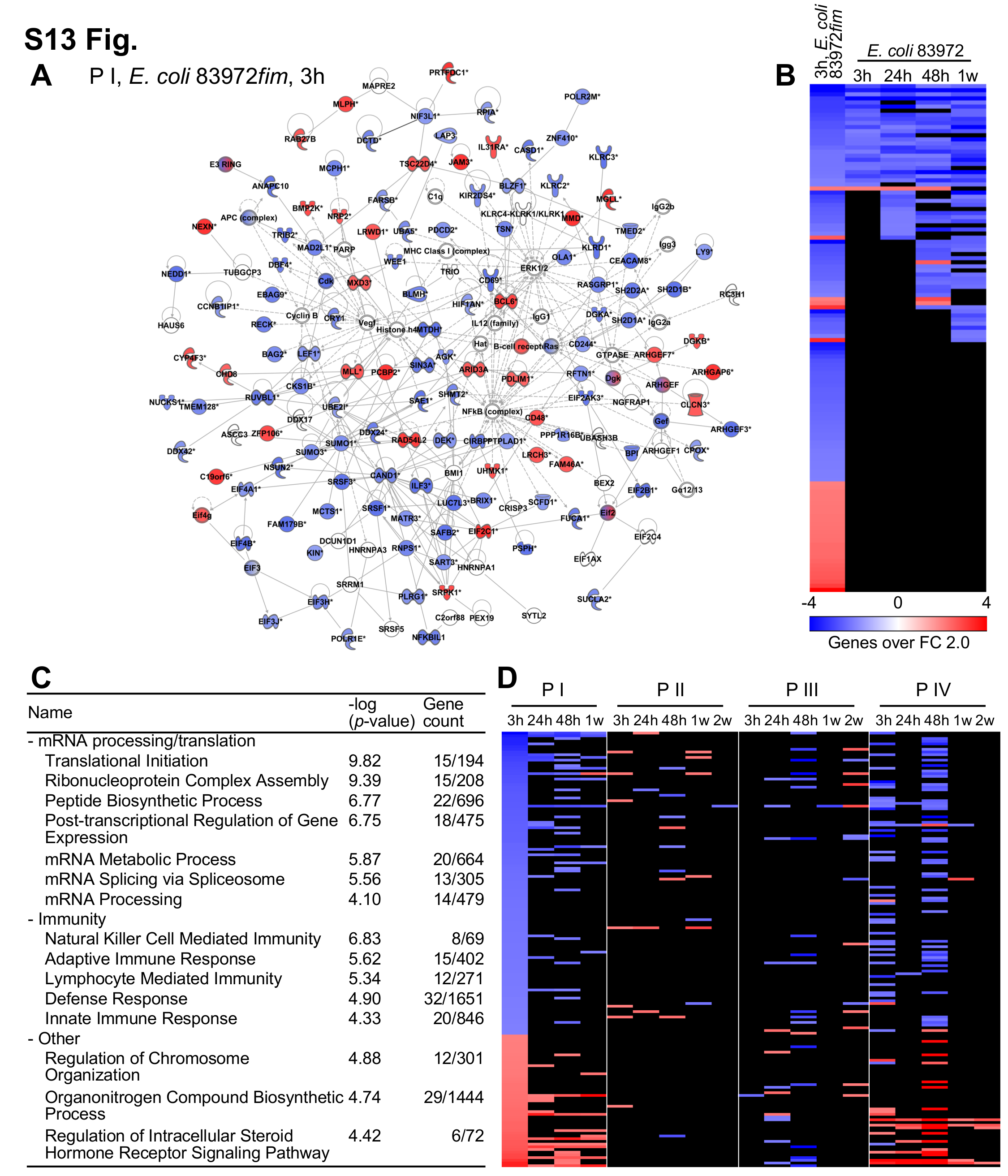

Supplement: S13 Fig — A. Rapid inhibition of host gene expression after inoculation with E. coli 83972fim in P I after 3 hours (61% of regulated genes). B. Heat map comparing the 3 hours response to E. coli 83972fim in P I to E. coli 83972 from 3h to 1w. Inhibited genes were shared, with more rapid kinetics for E. coli 83972fim than E. coli 83972. In addition, a set of genes was specific for E. coli 83972fim. C. Biological processes regulated by genes in the E. coli 83972fim “mega network”. Top regulated functions included RNA processing, RNA translation and immune related functions. D. Inhibitory profile especially in P I and P IV, which lasted for at least 48 hours but was lost thereafter. Red = FC ≥ 2.0 and blue = FC ≤ -2.0. (TIF) [file ppat.1007671.s013.tif]

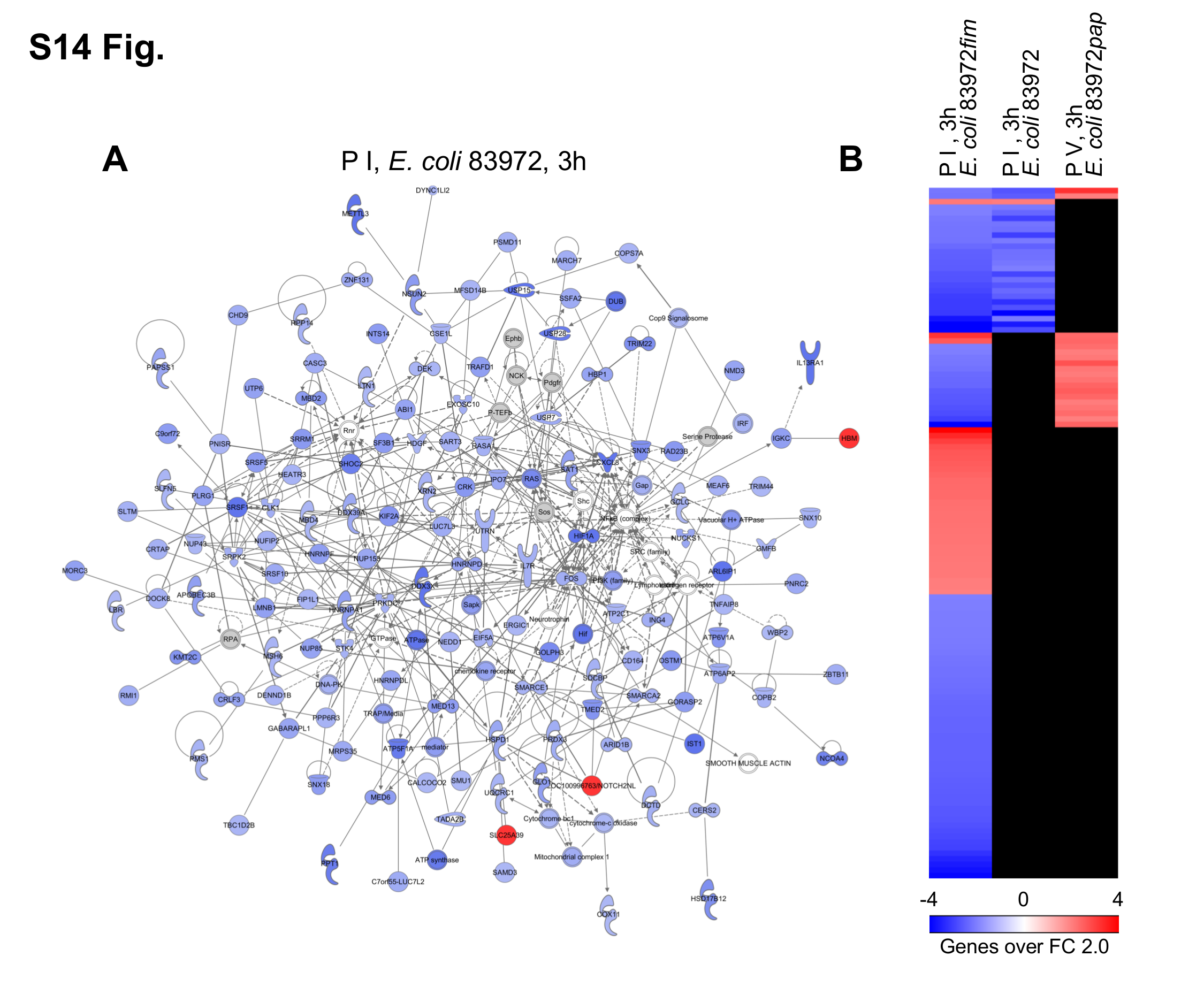

Supplement: S14 Fig — A. Rapid inhibition of gene expression, after inoculation with E. coli 83972 (P I, 3 hours). A “mega-network” was generated by merging the five top-scoring expression networks detected by IPA. B. Heatmap illustrating the differential effect of E. coli 83972fim and E. coli 83972pap on host gene expression. The regulation of genes in the P I, 3 hours, E. coli 83972fim network is shown (see Fig 6A). Red = FC ≥ 2.0 and blue = FC ≤ -2.0. (TIF) [file ppat.1007671.s014.tif]
