# Supplementary material for: Fimbriae reprogram host gene expression – Divergent effects of P and type 1 fimbriae
Source: PLoS Pathog. 2019 Jun 10;15(6):e1007671. doi: 10.1371/journal.ppat.1007671 (PMC6557620; doi:10.1371/journal.ppat.1007671)

S15A Fig.

E. coli 83972, 3h, all patients

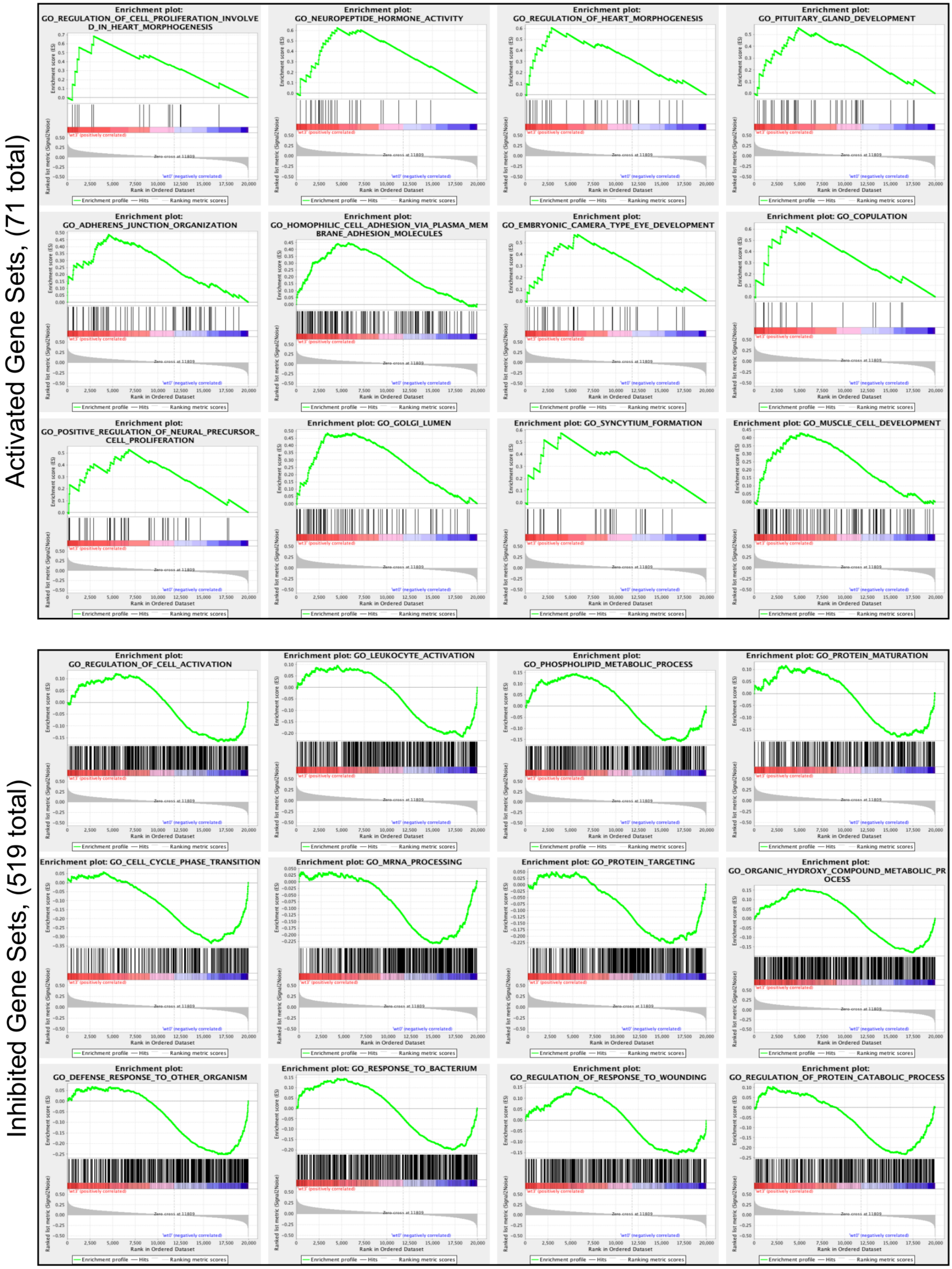

S15B Fig.

E. coli 83972, 24h, all patients

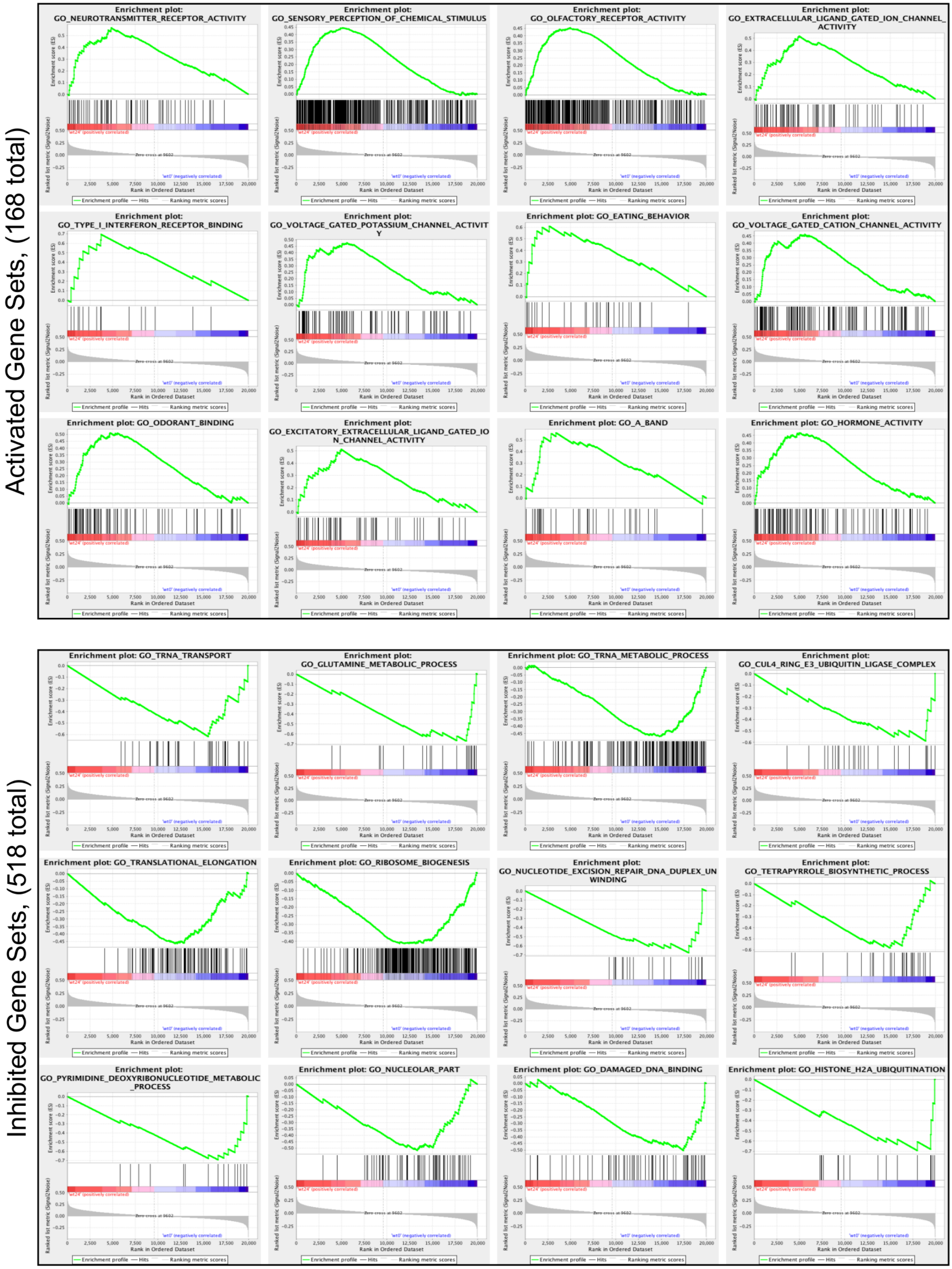

S15C Fig.

E. coli 83972, 48h, all patients

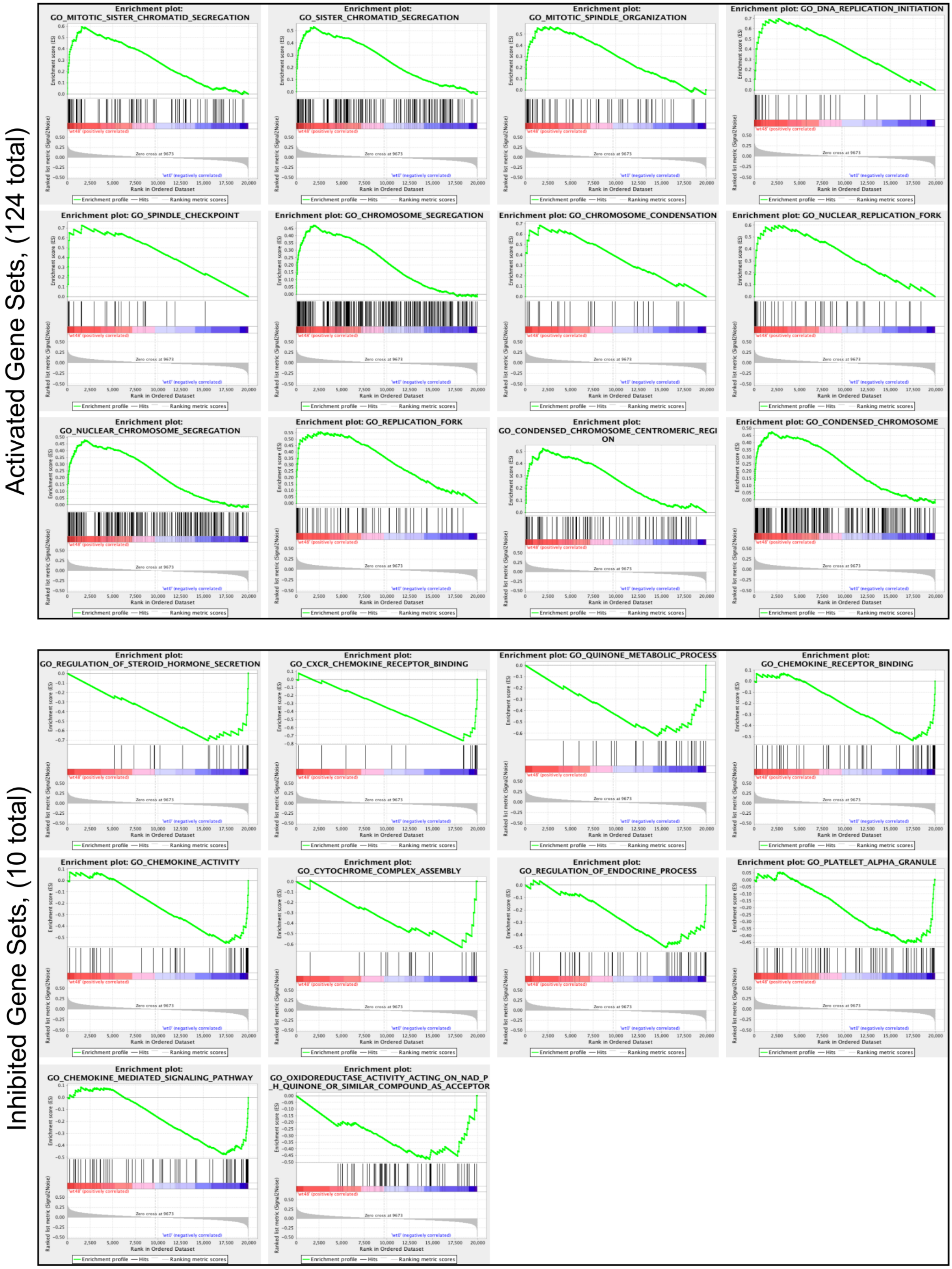

S15D Fig. *E. coli* 83972, 1w, all patients

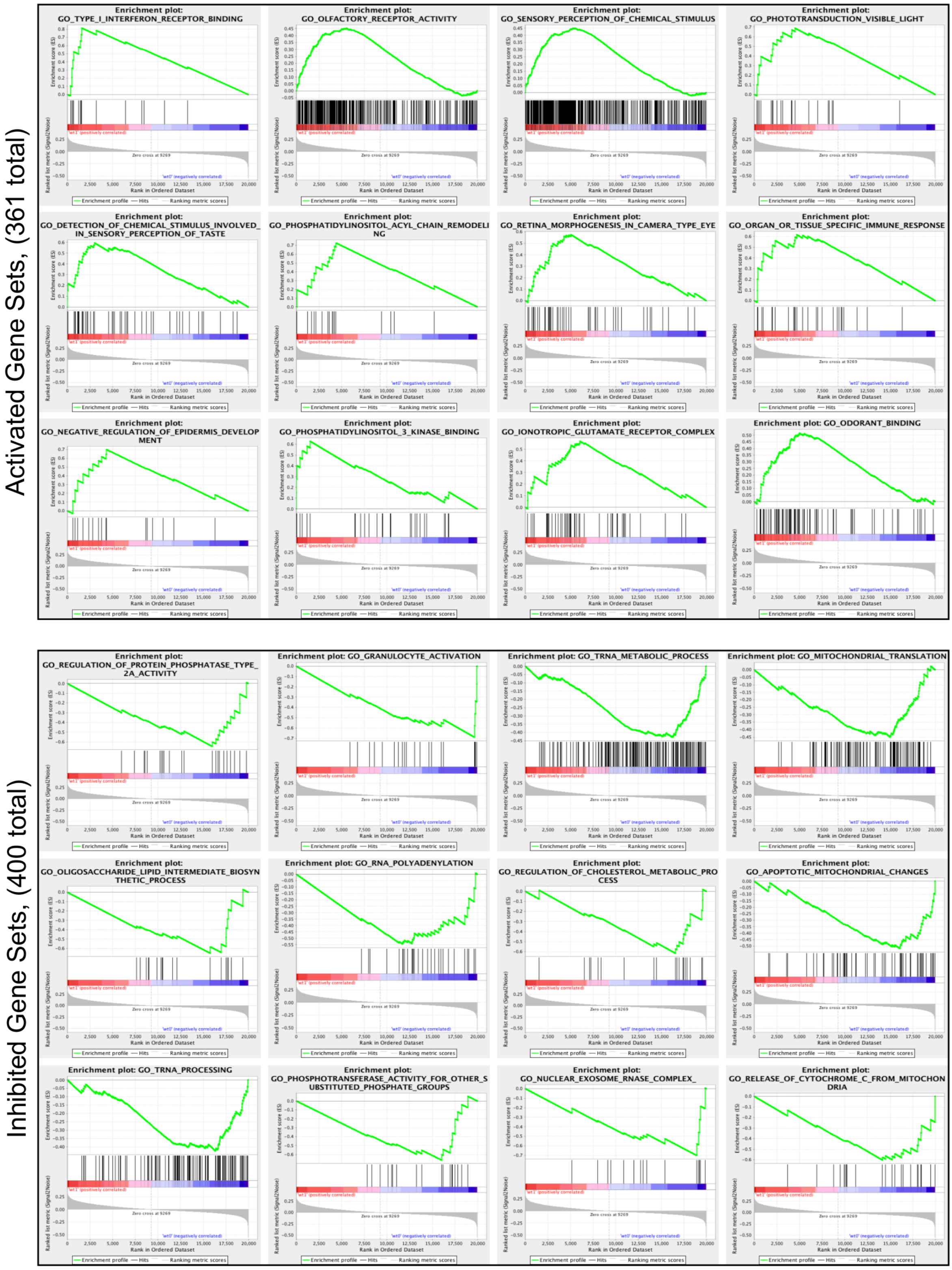

S15E Fig.

E. coli 83972*fim*, 3h, all patients

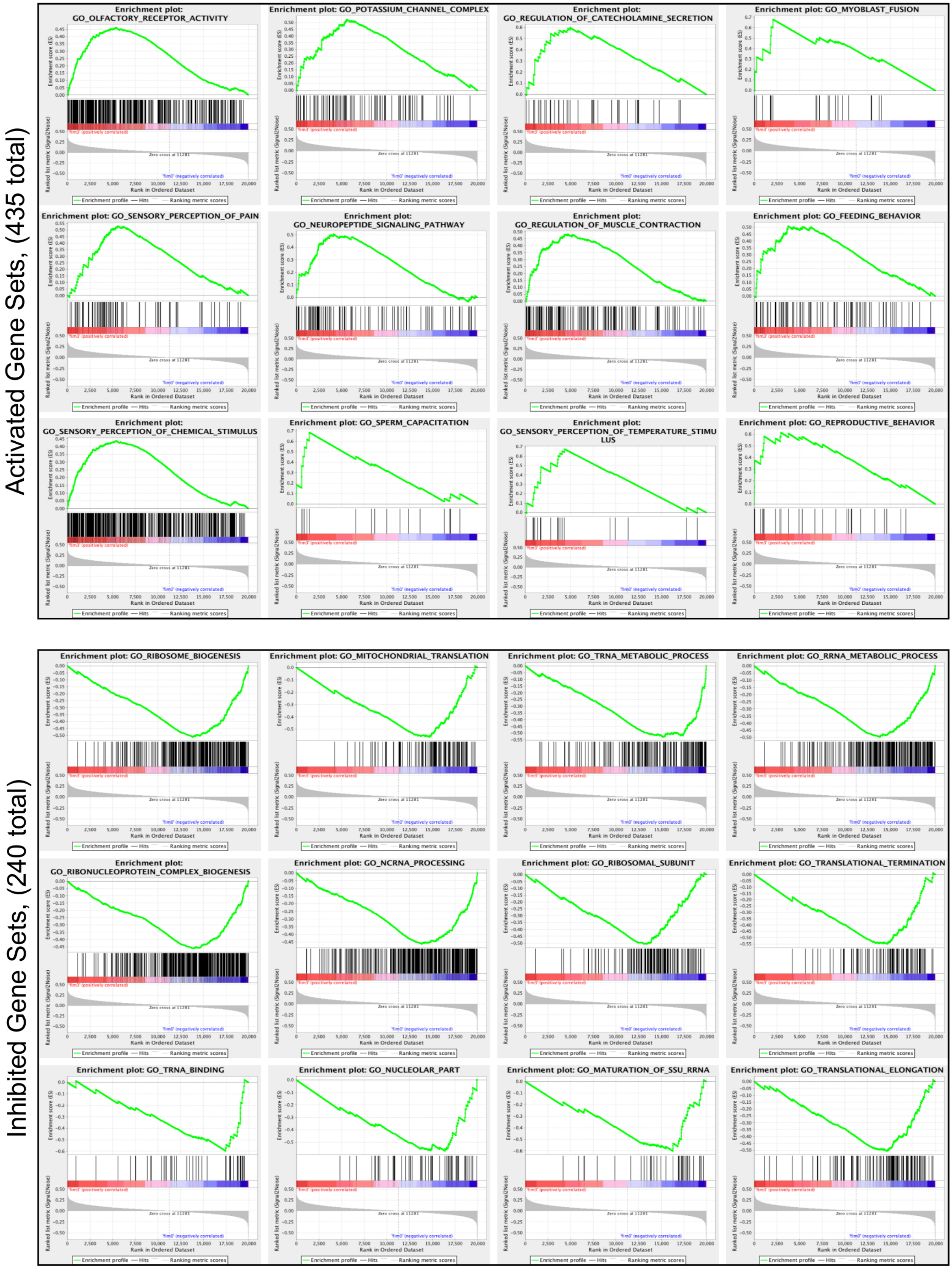

S15F Fig.

E. coli 83972*fim*, 24h, all patients

Activated Gene Sets, (290 total)

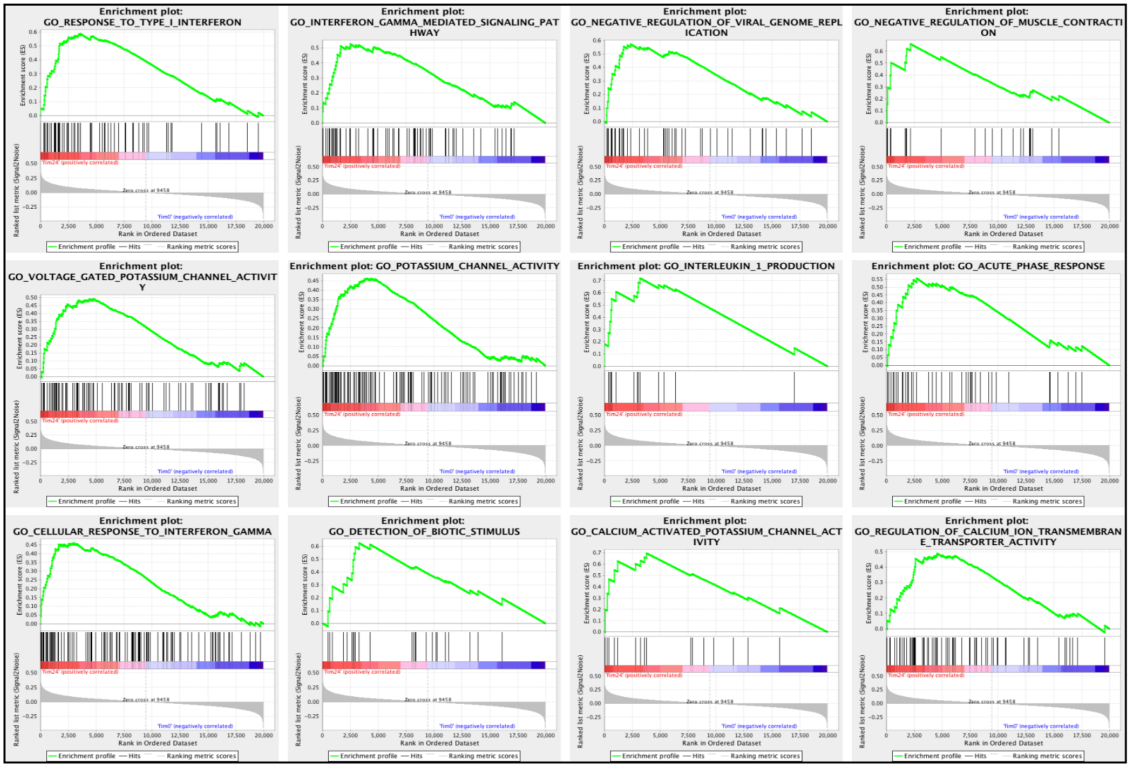

Inhibited Gene Sets, (53 total)

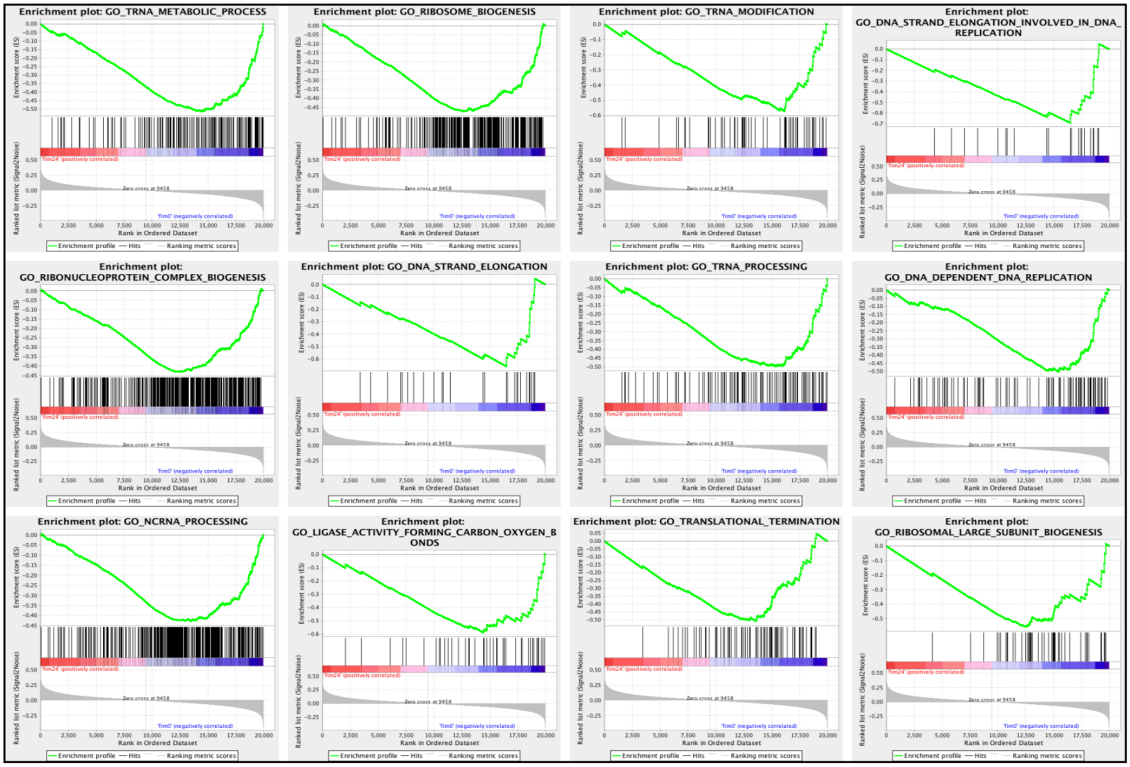

S15G Fig. *E. coli* 83972*fim*, 48h, all patients

Activated Gene Sets, (912 total)

Inhibited Gene Sets, (408 total)

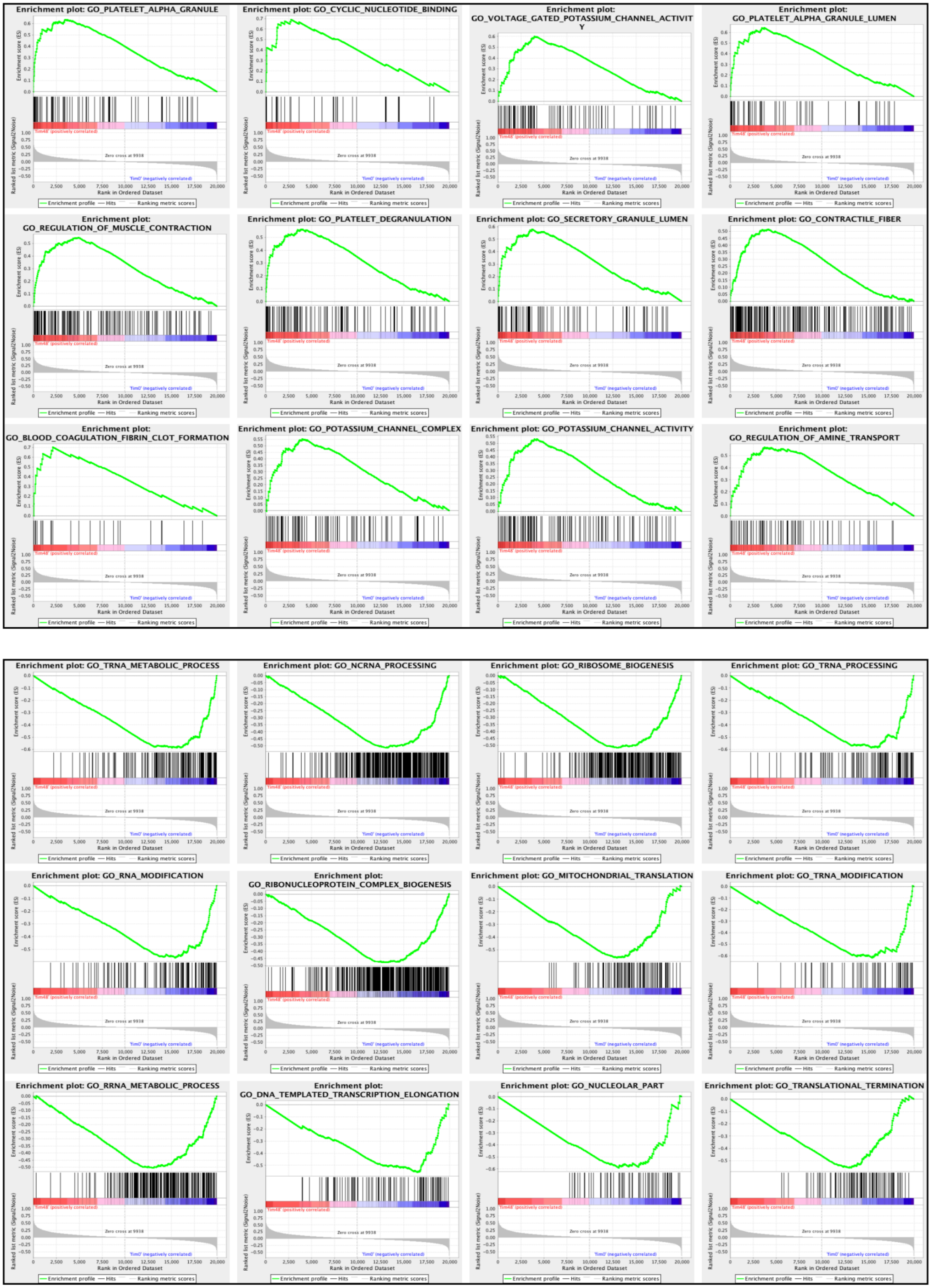

S15 Fig.

H *E. coli* 83972*fim*, 1w, all patients

Activated Gene Sets, (1 total)

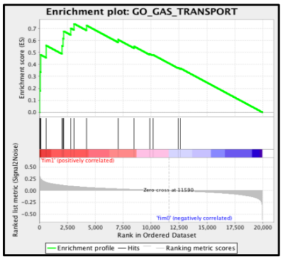

Inhibited Gene Sets, (3 total)

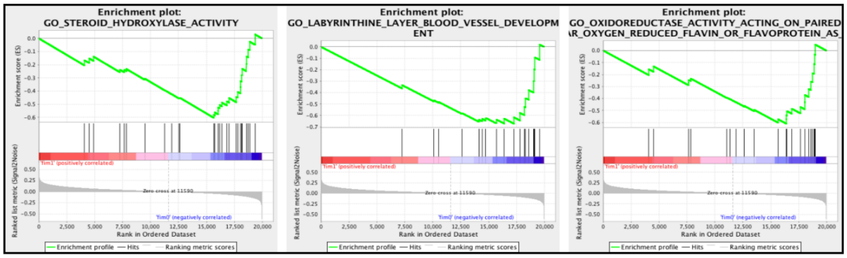

I *E. coli* 83972*pap*, 3h, all patients

Activated Gene Sets, (0 total)

Inhibited Gene Sets, (3 total)

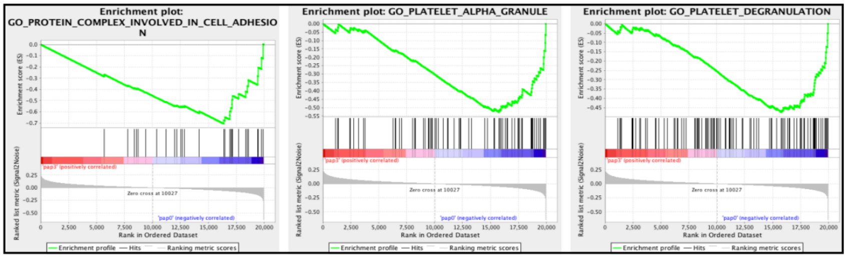

J *E. coli* 83972*pap*, 24h, all patients

Activated Gene Sets, (5 total)

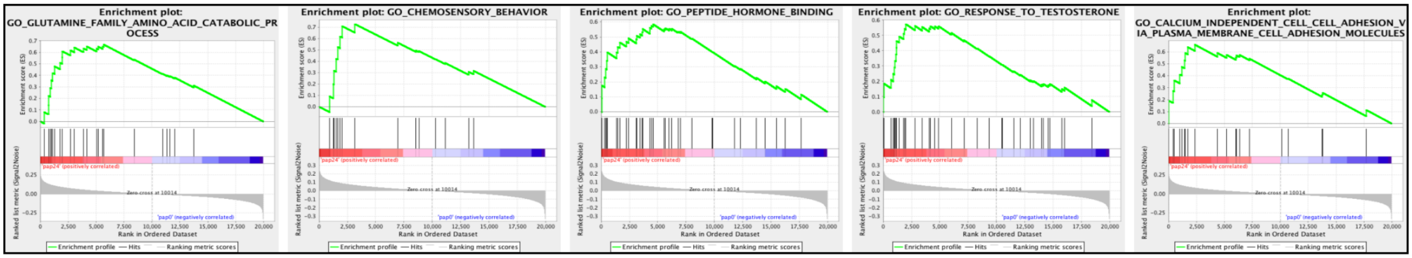

Inhibited Gene Sets, (0 total)

K *E. coli* 83972*pap*, 48h, all patients

Activated Gene Sets, (0 total)

Inhibited Gene Sets, (2 total)

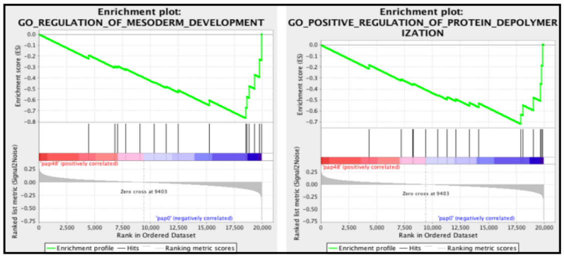

S15L Fig. *E. coli* 83972*pap*, 1w, all patients

Activated Gene Sets, (264 total)

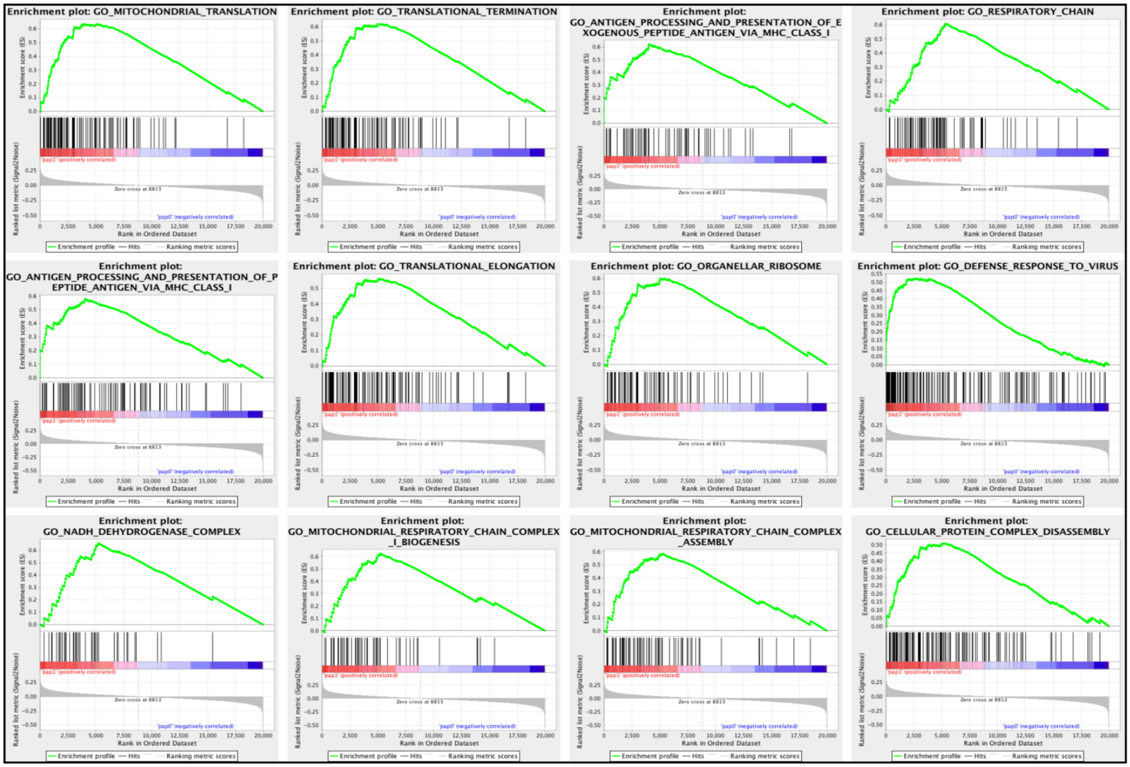

Inhibited Gene Sets, (2 total)

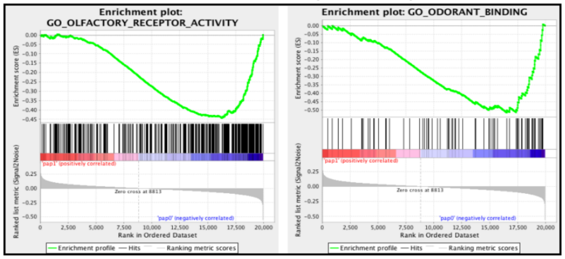

Supplement: S15 Fig — Gene Set Enrichment Analysis was performed by analyzing gene expression compared to pre-inoculation samples of all patients and after inoculation with E. coli 83972 (A-D), E. coli 83972fim (E-H) or E. coli 83972pap (I-L). Enrichment plots of the 12 most strongly regulated gene sets are shown. Gene sets with False Discovery Rate <25% are considered significantly enriched. (PDF) [file ppat.1007671.s015.pdf]
