# Supplementary material for: Fimbriae reprogram host gene expression – Divergent effects of P and type 1 fimbriae
Source: PLoS Pathog. 2019 Jun 10;15(6):e1007671. doi: 10.1371/journal.ppat.1007671 (PMC6557620; doi:10.1371/journal.ppat.1007671)
Supplement: S1 Table — (PDF) [file ppat.1007671.s016.pdf]

S1 Table. Patient characteristics and inoculation schedule.

Patient ID	Sex	Year of birth	CIC	*Inoculations with E. coli 83972*[1]			*IRF3* GP[2]	*TLR4* GP[3]	Diagnosis
				wt	*fim*	*pap*			
I	M	1926	+	*b*	*c*		CCAA	IV	Idiopathic detrusor insufficiency
II	M	1965	+	*a*	*b*	*c*	CTAG	IV	Paraplegia; spinal cord injury
III	F	1971	-	*a*	*b*	*c*	CCAA	IV	Idiopathic detrusor insufficiency
IV	M	1931	+	*a*	*b*	*c*	CCAA	XIX	Detrusor insufficiency; history of encephalomyelitis
V	F	1939	-	*a*		*b*	CCAA	VII	Idiopathic detrusor insufficiency

All patients had incomplete voiding (residual urine between 50-300 ml; if > 100 ml treated with Clean Intermittent Catheterization, CIC), and had recurrent UTI. Inoculations were performed during a four-year period (Oct 2007 - June 2011). Time between inoculations ranged between 4-13 months (median 6 months). For genotyping, patient DNA was extracted from heparinized peripheral blood using QIAmp DNA Blood Midi Kit (Qiagen). Genotyping was performed using a Pyrosequencer (PSQ 96, Biotage) after PCR amplification of patient DNA and a second biotinylated PCR for each SNP (for primers and clinical relevance see (Akahoshi et al., 2008, Fischer et al., 2010, Ragnarsdottir et al., 2010).

1) *a, b, c* means the time order for the first (*a*), second (*b*) or third *(c)* successful inoculation performed in the patient.

2) The *IRF3* promoter polymorphisms (-925 and -776) genotype pattern (GP) C/C, A/A is predominant in patients with recurrent pyelonephritis and has been reported to reduce promoter efficacy (Fischer et al., 2010). The GP C/T, A/G is predominant in patients with ABU (Fischer et al., 2010) and is associated with a lower host response in patients with *E. coli* 83972 (Gronberg-Hernandez et al., 2011).

3) The *TLR4* promoter GP IV is the predominant GP in the Swedish population. GP VII reduces *TLR4* promoter activity. GP XIX is rarely present in the Swedish population, and its effect has not been determined (Ragnarsdottir et al., 2010).
