# Supplementary material for: Fimbriae reprogram host gene expression – Divergent effects of P and type 1 fimbriae
Source: PLoS Pathog. 2019 Jun 10;15(6):e1007671. doi: 10.1371/journal.ppat.1007671 (PMC6557620; doi:10.1371/journal.ppat.1007671)
Supplement: S2 Table — (PDF) [file ppat.1007671.s017.pdf]

S2 Table. Top 20 up- and down-regulated Gene ontology gene sets regulated by *E. coli* 83972*fim* after 3 hours.

Gene set name (Gene Ontology)	Size	NES	Nom. p-val
- Ribosome related gene sets:			
Ribosome Biogenesis	292	-2.63	<0.001
rRNA Metabolic Process	243	-2.58	<0.001
Ribonucleoprotein Complex Biogenesis	417	-2.50	<0.001
Ribosomal Subunit	153	-2.48	<0.001
Nucleolar Part	58	-2.35	<0.001
Maturation of SSU rRNA	39	-2.34	<0.001
Preribosome	56	-2.31	<0.001
Ribosome	211	-2.31	<0.001
Small Ribosomal Subunit	62	-2.29	<0.001
Large Ribosomal Subunit	91	-2.28	<0.001
Cytosolic Ribosome	104	-2.25	<0.001
- Translation related gene sets:			
Mitochondrial Translation	104	-2.60	<0.001
tRNA Metabolic Process	169	-2.59	<0.001
Translational Termination	90	-2.45	<0.001
tRNA Binding	44	-2.36	<0.001
Translational Elongation	108	-2.34	<0.001
tRNA Processing	108	-2.29	<0.001
- Other gene sets:			
ncRNAProcessing	369	-2.49	<0.001
Nucleotide Excision Repair DNA Incision	38	-2.26	<0.001
Amino Acid Activation	52	-2.26	<0.001
- Ion Channels related gene sets:			
Potassium Channel Complex	90	1.99	<0.001
Ion Antiporter Activity	44	1.89	<0.001
Antiporter Activity	70	1.88	<0.001
- Neurotransmitter and Sensing related gene sets:			
Olfactory Receptor Activity	336	1.99	<0.001
Regulation of Catecholamine Secretion	43	1.96	<0.001
Sensory Perception of Pain	75	1.93	<0.001
Neuropeptide Signaling Pathway	97	1.92	<0.001
Sensory Perception of Temperature Stimulus	21	1.92	0.001
Sensory Perception of Chemical Stimulus	430	1.91	<0.001
Regulation Of AMPA Selective Glutamate Receptor Activity	19	1.90	<0.001
Neuron Neuron Synaptic Transmission	55	1.87	<0.001
- Muscle related gene sets:			
Myoblast Fusion	20	1.96	<0.001
Regulation of Muscle Contraction	147	1.95	<0.001
Regulation of Smooth Muscle Contraction	60	1.89	<0.001
- Immune response related gene sets:			
Complement Activation	47	1.91	0.001
Humoral Immune Response Mediated By Circulating Immunoglobulin	41	1.87	<0.001
- Other gene sets:			
Feeding Behavior	91	1.92	<0.001
Membrane Invagination	31	1.91	<0.001
Sperm Capacitation	18	1.91	0.002
Golgi Lumen	84	1.88	<0.001
