# Supplementary material for: Fimbriae reprogram host gene expression – Divergent effects of P and type 1 fimbriae
Source: PLoS Pathog. 2019 Jun 10;15(6):e1007671. doi: 10.1371/journal.ppat.1007671 (PMC6557620; doi:10.1371/journal.ppat.1007671)
Supplement: S3 Table — (PDF) [file ppat.1007671.s018.pdf]

S3 Table. Primers used in the study.

SNP	Forward	Reverse	Sequencing
frr amplification	CTTGTTCTGCTTCACCACGA	GGCAAGCGTAACGGTAGAAG	
fimA amplification	TAGGACAGGTTCGTACCGCATCG	TGTCCAGGATCTGCACACCAACG	
papA amplification	TGAAACGCAGTCTGCAAGACAG	CGCCAACTGTTTGCAGCATATC	
IRF3 promoter amplification	CTCAATTTGCATGTGACGCTCCCAG	CTTGCTCCACTTTCCCCAGAGTACAC	
IRF3 -925 genotyping	GCATAGGGGCGGGAACAG	CGGGTAGCTCTCAAACTCGAGG-BIO	GGTTTTATTTCAAGAAGTCG
IRF3 -776 genotyping	TCGTAGGTAACGCGGAAGA	CACTTTCCCCAGAGTACACAGC-BIO	TAACGCGGAAGAGCC
TLR4 -4038 genotyping	CCTGCCAAATAAAAGCAAACAC	CAGTCAATGGAGGAATCCCATAT-BIO	TTCTAGGTCCCTGGC
TLR4 -3612 genotyping	TTTGTATTTGACACATGGTCTGC	TGGGAAATGAATGGGATTAACAC-BIO	CATTACTATTGAACATATCC
TLR4 -3002 genotyping	BIO-AATCAATTGGAAGAGCTGGTACA	CAGTCGCCATTTCTACTACCATTA	CATTACTATTGAACATATCC
TLR4 -2604 genotyping	BIO-TCAGTGGGCTCTGGGGTAG	CAGCCCTAATCATCACAGGTC	CATCACAGGTCCAGGTA
TLR4 -2570 genotyping	BIO-TGGTACCTGGACCTGTGATGAT	CCTCCTCTACCTGGCTTTTACA	GCTTTTACACCCAAGTAGAC
TLR4 -2081 genotyping	TACAAGAGTTTGTGCCCAGTCCA	GCAAGTGCAATGTAAGTTTCTGTT-BIO	CCCTCACAGCTTGGTT
TLR4 -2026 genotyping	TTGGAAGTGCTTGGAGGATATTA	TTAGGACAGTGTCTGGAAAGTAGC-BIO	AGAACTATCTAGGACTTAGC
TLR4 -1607 genotyping	BIO-AAATGCAAGCTTCTGCTATGATTA	TCAGAAGTGAGATTGCTGGATCAT	TTTCACATCTTCACCAAC
IRF7 promoter amplification	TTCCTCTCCCGCTCTAACCA	GTGGACTGAGGGCTTGTAGC	
